# Using IR vibrations to quantitatively describe and predict site-selectivity in multivariate Rh-catalyzed C–H functionalization†
†Electronic supplementary information (ESI) available: Experimental procedures, tabulated descriptors, and model development MATLAB commands. See DOI: 10.1039/c5sc00357a


**DOI:** 10.1039/c5sc00357a

**Published:** 2015-03-18

**Authors:** Elizabeth N. Bess, David M. Guptill, Huw M. L. Davies, Matthew S. Sigman

**Affiliations:** a Department of Chemistry , University of Utah , 315 South 1400 East , Salt Lake City , UT 84112 , USA . Email: sigman@chem.utah.edu; b Department of Chemistry , Emory University , 1515 Dickey Drive , Atlanta , GA 30322 , USA . Email: hmdavie@emory.edu

## Abstract

Achieving selective C–H functionalization is a significant challenge that requires discrimination between many similar C–H bonds. Yet, reaction systems employing Rh_2_(DOSP)_4_ and Rh_2_(BPCP)_4_ were recently demonstrated to afford high levels of selectivity in the C–H insertion of carbenes into toluene-derived substrates. Herein, we explore the origin of this selectivity through a systematic analysis of substrate and reagent features that alter levels of selectivity from 20 : 1 to 1 : 610 for secondary (or tertiary)-to-primary benzylic C–H functionalization of toluene derivatives. Describing this variation using infrared vibrations and point charges, we have developed a mathematical model from which are identified features of the systems that determine levels of site-selectivity and are applied as predictive factors to describe the selectivity behavior of new substrate/reagent combinations.

## Abstract 2

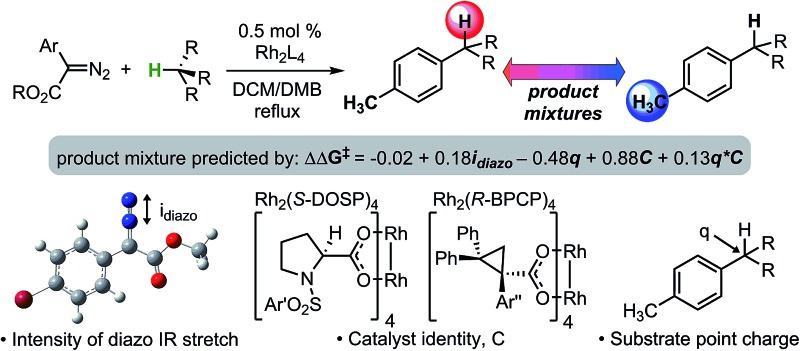
Achieving selective C–H functionalization is a significant challenge that requires discrimination between many similar C–H bonds.

## Introduction

The abundance of C–H bonds in any given molecule presents a major challenge to the development of site-selective C–H functionalization methods.^1^ Out of this challenge arises an objective to identify and understand the chemical precepts and interactions that govern site-selectivity in such reactions.^2^ With increasingly precise knowledge of these outcome-defining molecular features, a methodology can be better refined and optimized, with the ultimate goal of developing predictive models and tailoring systems to yield prescribed reaction outcomes.

**Fig. 1:**
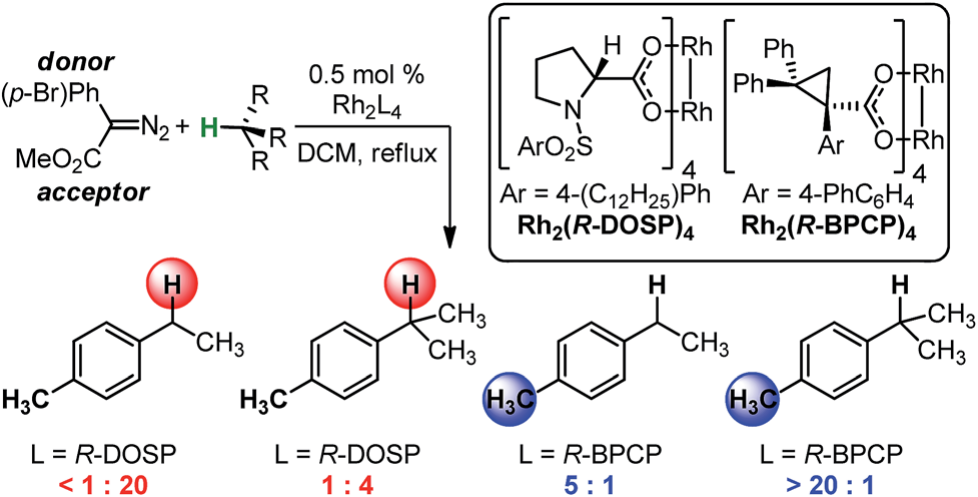
Davies and colleagues' Rh-catalyzed C–H functionalization reaction, demonstrating site-selectivity sensitivity. Preferred C–H bond for functionalization under given conditions is highlighted.

An impressive example of site-selective C–H functionalization is the C–H insertion reaction of donor/acceptor rhodium-carbenes.^3^,^4^ This process is proposed to proceed through a hydride transfer-like event from the substrate to the carbene, with subsequent C–C bond formation.^5^ It is generally observed in these systems that more substituted (or activated) C–H bonds are electronically favored and primary C–H bonds are sterically preferred. With the most established catalyst, Rh_2_(*S*-DOSP)_4_, these effects are balanced so as to favor functionalization of secondary C–H bonds (Fig. 1).^3^ This tendency can be overcome to favor functionalization of primary C–H bonds when using a more sterically demanding catalyst, Rh_2_(*R*-BPCP)_4_ (Fig. 1).^6^,^7^ Nevertheless, the effect of the ester substituent^7^ and functionality on the donor group^8^ remain relatively unexplored as elements of control over site-selectivity. Understanding the origin of selectivity is thus a major goal in the design and development of new reagents and catalysts.^6^,^7^

While there are many diverse means of exploring the molecular features at the origin of selectivity,^9^ the Sigman group has focused on a method that derives from linear free-energy relationship analysis. In short, mathematical relationships are developed that equate the differential transition state free energy between pathways affording isomeric products (ΔΔ*G*^‡^) to numeric depictions of molecular features.^10^,^11^ Of the various parameters available, computationally measured infrared (IR) bond vibrations have recently emerged as particularly apt descriptors of the molecular characteristics that impact selectivity.^10^ The intrinsic ability of IR vibrations to accurately represent a molecule's multifaceted, fundamental structure has enabled the mathematical description of complex systems.^12^ However, the density of information manifested in an IR vibration can also make this descriptor difficult to interpret, clouding the origin of a vibration's importance for mathematically describing trends in selectivity.

Herein, we investigate a method of decoding the controlling elements of rhodium-catalyzed C–H insertion of donor/acceptor carbenes *via* free-energy mathematical models (Fig. 1).^6^ Using IR vibrations, we have developed quantitative relationships that describe a substantial range (20 : 1 to 1 : 610) in ratios of secondary (or tertiary)-to-primary benzylic C–H functionalization of toluene derivatives. These relationships begin to inform the design of the optimum reagent/catalyst combination for reaction at a specific C–H bond and for the prediction of site-selective outcomes.

## Methodology

### Experimental design

**Fig. 2:**
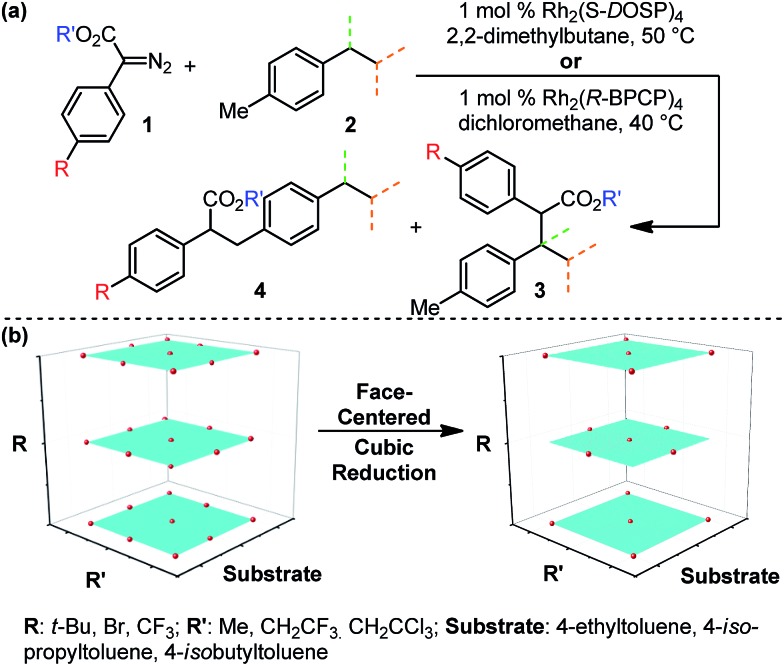
(a) Identification of three reaction aspects for each Rh-catalyzed reaction that can be systematically varied to determine their influence on site selectivity. (b) Full DoE matrix and face-centered cubic reduction matrix, which defines a simplified approach to the systematic evaluation of each Rh-catalyzed system.

Evaluation of the Rh_2_(*R*-BPCP)_4_- and Rh_2_(*S*-DOSP)_4_-catalyzed C–H insertion reactions of toluene derivatives commenced with construction of a design of experiments (DoE)-founded matrix to systematically evaluate diazo ester features that impact site selection (Fig. 2).^13^,^14^ This matrix was built upon the hypothesis that electron-donating and -withdrawing arene substituent effects (R) will influence reaction outcomes in tandem with steric and/or electronic contributions from the ester moiety (R′). Thus, the arene of **1** was assessed according to a Hammett series of electronically varied substituents, and the ester was evaluated *via* a set of sterically and electronically diverse groups. Finally, the substrate was considered to be an additional dimension. As such, the catalysts' abilities to discriminate between primary and secondary or tertiary C–H bonds, adjacent to different steric environments, could be gauged by installation of ethyl, isopropyl, and isobutyl substituents at the *para*-position in toluene substrates.

To systematically and efficiently assess combinations of these three variables, a face-centered cubic design matrix was implemented to define experimental evaluation of the reaction (Fig. 2b).^14^ The resulting suite of arene, ester, and substrate combinations was assessed in the Rh_2_(*S*-DOSP)_4_ and Rh_2_(*R*-BPCP)_4_ catalytic systems, affording an array of secondary (or tertiary)-to-primary benzylic C–H functionalization ratios that range from 20 : 1 to 1 : 610, respectively (Tables 1 and 2). In the Rh_2_(*S*-DOSP)_4_-catalyzed reactions (Table 1), insertion into a secondary C–H bond preferentially occurs, as previously observed, but there is a considerable difference in the selectivity depending on the nature of the aryl substituent and the ester group. In the Rh_2_(*R*-BPCP)_4_-catalyzed reactions (Table 2), insertion into a primary C–H bond is strongly favored, although a range is also measured.

**Table 1:** Results of Rh_2_(*S*-DOSP)_4_-catalyzed carbene insertion reaction used to develop the descriptive model in Fig. 5a

Entry^*a*^	R	R′	Toluene Substrate	Meas. 3 : 4	Meas. ΔΔ*G*^‡^ (kcal mol^–1^)	Pred. ΔΔ*G*^‡^ (kcal mol^–1^)
1	*t*-Bu	Me	4-Ethyl	20.0 : 1.0	1.92	1.90
2	CF_3_	Me	4-Ethyl	10.0 : 1.0	1.48	1.64
3	Br	Me	4-Isopropyl	1.9 : 1.0	0.41	0.68
4	Br	CH_2_CF_3_	4-Ethyl	11.0 : 1.0	1.54	1.50
5	*t*-Bu	CH_2_CF_3_	4-Isopropyl	4.5 : 1.0	0.97	0.34
6	Br	CH_2_CF_3_	4-Isopropyl	1.8 : 1.0	0.38	0.31
7	CF_3_	CH_2_CF_3_	4-Isopropyl	1.4 : 1.0	0.22	0.10
8	*t*-Bu	CH_2_CCl_3_	4-Ethyl	9.0 : 1.0	1.41	1.28
9	CF_3_	CH_2_CCl_3_	4-Ethyl	4.8 : 1.0	1.01	1.05
10	Br	CH_2_CCl_3_	4-Isopropyl	1.0 : 2.1	–0.48	0.07
11	Cl	Me	4-Ethyl	13.0 : 1.0	1.65	1.80
12	Br	Et	4-Ethyl	12.3 : 1.0	1.61	1.80
13	Br	CH_2_CBr_3_	4-Ethyl	5.8 : 1.0	1.13	1.15
14	OMe	Me	4-Isopropyl	4.7 : 1.0	0.99	0.73
15	OMe	CH_2_CCl_3_	4-Isopropyl	1.3 : 1.0	0.17	–0.03
16	Br	CH_2_CCl_3_	4-Ethyl	6.6 : 1.0	1.21	1.26

^*a*^Entries 1–10, training set; entries 11–16, external validation set.

**Table 2:** Results of Rh_2_(*R*-BPCP)_4_-catalyzed carbene insertion reaction used to develop the descriptive model in Fig. 5b

Entry^*a*^	R	R′	Toluene Substrate	Meas. 3 : 4	Meas. ΔΔ*G*^‡^ (kcal mol^–1^)	Pred. ΔΔ*G*^‡^ (kcal mol^–1^)
1	*t*-Bu	Me	4-Ethyl	1.0 : 3.7	–0.81	–0.68
2	CF_3_	Me	4-Ethyl	1.0 : 5.2	–1.03	–0.99
3	Br	Me	4-Isopropyl	1.0 : 95.0	–2.83	–2.80
4	Br	CH_2_CF_3_	4-Ethyl	1.0 : 5.1	–1.01	–1.15
5	*t*-Bu	CH_2_CF_3_	4-Isopropyl	1.0 : 101.0	–2.87	–3.21
6	Br	CH_2_CF_3_	4-Isopropyl	1.0 : 182.0	–3.24	–3.24
7	CF_3_	CH_2_CF_3_	4-Isopropyl	1.0 : 213.0	–3.33	–3.49
8	*t*-Bu	CH_2_CCl_3_	4-Ethyl	1.0 : 10.0	–1.43	–1.41
9	CF_3_	CH_2_CCl_3_	4-Ethyl	1.0 : 14.0	–1.64	–1.69
10	Br	CH_2_CCl_3_	4-Isopropyl	1.0 : 610.0	–3.99	–3.52
11	Cl	Me	4-Ethyl	1.0 : 3.8	–0.83	–0.80
12	Br	Et	4-Ethyl	1.0 : 4.4	–0.92	–0.79
13	Br	CH_2_CBr_3_	4-Ethyl	1.0 : 14.0	–1.64	–1.57
14	OMe	CH_2_CCl_3_	4-Isopropyl	1.0 : 55.0	–2.49	–3.65
15	F	CH_2_CCl_3_	4-Isopropyl	1.0 : 437.0	–3.78	–3.76
16	Br	CH_2_*t*-Bu	4-Ethyl	1.0 : 7.4	–1.24	–1.05
17	Br	CH_2_CCl_3_	4-Ethyl	1.0 : 11.1	–1.50	–1.44
18	H	Me	4-Ethyl	1.0 : 3.0	–0.68	–0.99
19	Br	Me	4-Ethyl	1.0 : 4.1	–0.88	–0.71

^*a*^Entries 1–10, training set; entries 11–19, external validation set.

Reactions performed on 4-isobutyltoluene with Rh_2_(*R*-BPCP)_4_ lead to the detection of only primary C–H insertion products. Changing to Rh_2_(*S*-DOSP)_4_, insertion was favored at the primary site, but tertiary (rather than secondary, benzylic) insertion products were also observed. These results preclude the use of 4-isobutyltoluene in the remainder of these studies.

### Correlation of IR parameters to classical measurements

To initiate mathematical modelling of the selectivity ratios as a function of molecular features of the diazo ester and toluene substrate, potentially relevant mathematical descriptors were gathered. While *σ* may describe the appropriate changes to the arene ring (*vide infra*), it was anticipated that simultaneous variation of both the ester and arene would influence the reactivity and selectivity of the resultant carbene, which could not be described by Hammett *σ* or *σ*^+^ values alone. Consequently, it was of particular interest to identify an analogous parameter that would simultaneously embody both dimensions of modulation. Thus, the N≡N diazo IR stretching frequency and intensity were considered, as this functional group is positioned at the fusion of the two modulated moieties and the site of the eventual reactive carbene. It was hypothesized that the IR vibrational properties of the diazo might appropriately describe the electronic and, perhaps, steric influences that coalesce at this position.

**Fig. 3:**
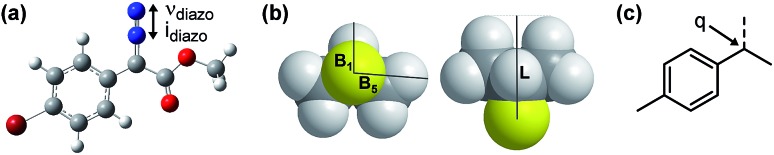
(a) Representation of computationally measured diazo IR stretch in energy-minimized structures. (b) Depiction of Sterimol values used to quantitate sterics of an isopropyl substituent. *B*_1_ is a minimum and *B*_5_ is a maximum radial bulk; *L* is substituent length. (c) NBO point charge used to describe electronic variation in the toluene-derived substrates investigated.

Due to the ease and fidelity with which computational IR measurements can be made and identical vibrational modes can be identified, IR frequencies and intensities were computed from energy-minimized diazo esters (M06-2X/TZVP, Fig. 3a).^15^ Although the diazo ester is not the active species that engages in C–H insertion, it likely shares significant structural similarities to the implicated rhodium-carbene. In analogy to the classical application of Hammett *σ*-parameters, we hypothesized that the rhodium-carbene's *relative* steric and electronic features would be conserved in its closely related precursor, a diazo ester. Additionally, performing computations on this simple, ground-state organic molecule is a facile exercise that obviates the greater computational load required for transition metal computation, especially when there is only one characterized example for this complex.^16^

As the goal of this study was not only to develop comprehensive models for prediction purposes but also to better understand the origin of site-selectivity, it was of interest to investigate what the N≡N diazo IR stretching frequency and intensity may represent in terms of classical physical organic parameters. Therefore, a MATLAB stepwise linear regression algorithm was used to find the optimal combinations of more simple parameters that describe the observed changes in *ν*_diazo_ and *i*_diazo_.^17^ These parameters include Sterimol measures of substituents' steric effects (*B*_1_, minimum radial bulk; *B*_5_, maximum radial bulk; *L*, substituent length—Fig. 3b),^18^ molecular weight of R′, p*K*_a_ of the corresponding R′ alcohol (R′–OH), and arene *σ* and *σ*^+^.

**Fig. 4:**
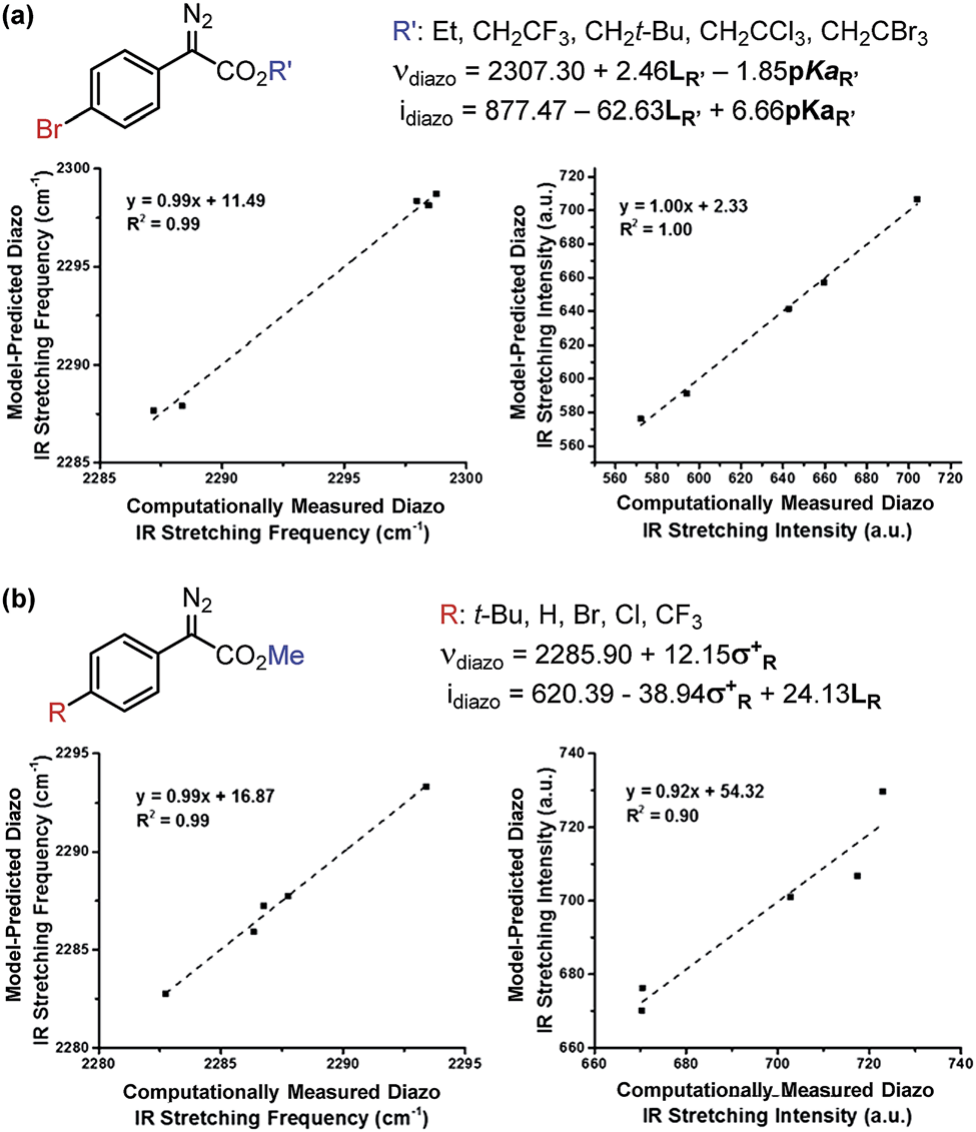
Determination of which factors contribute to diazo IR stretching frequencies and intensities upon independent variation of the (a) ester and (b) arene moieties.

The results of these analyses are given in Fig. 4a. The effect of the ester substituent R′ on both *ν*_diazo_ and *i*_diazo_ is best related by the Sterimol length (*L*_R′_) and R′–OH p*K*_a_, which combine to effectively describe the observed trends in both IR measurements, although with different weights of importance. Of specific interest, groups with enhanced length increase the frequency whereas groups that are less able to stabilize negative charge result in decreased *ν*_diazo_. The opposite scenario is described by the *i*_diazo_ model: vibrational intensity is decreased as the length increases but raised when R′ substituents bear higher p*K*_a_ values. Arene influences on the diazo IR vibration were similarly assessed (Fig. 4b). A positive, nearly one-to-one correlation between *ν*_diazo_ and *σ*^+^ is demonstrated, depicting a solely electronic contribution of *para*-substituted arenes on this IR frequency (Fig. 4b).^19^ Alternatively, an inverse correlation exists between *i*_diazo_ and *σ*^+^, which is attenuated by mass at R, as described by Sterimol *L*.

### Model development

**Fig. 5:**
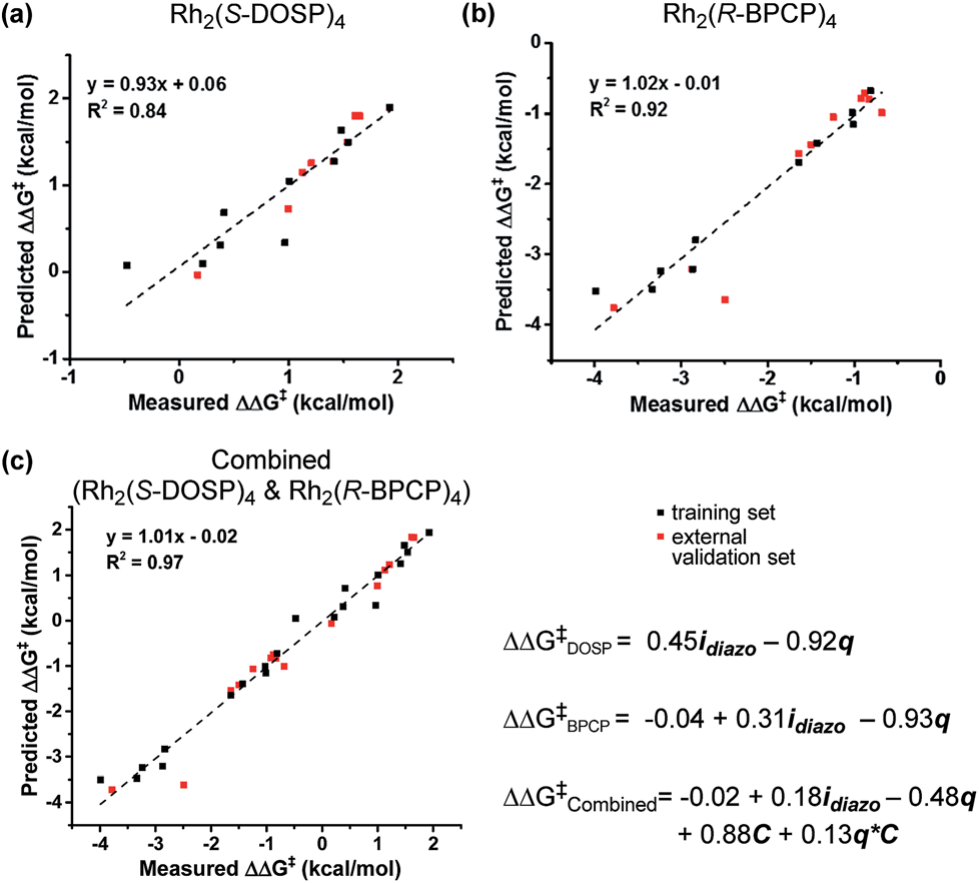
Normalized descriptive mathematical models and associated model-predicted *versus* experimentally measured ΔΔ*G*^‡^ values for the (a) Rh_2_(*S*-DOSP)_4_ and (b) Rh_2_(*R*-BPCP)_4_ systems, and (c) the combination thereof.

With a more detailed understanding of the factors that contribute to *ν*_diazo_ and *i*_diazo_ variation, linear regression was performed using the training sets presented in Tables 1 and 2 (Rh_2_(*S*-DOSP)_4_ and Rh_2_(*R*-BPCP)_4_ systems, respectively). From the parameters *ν*_diazo_ and *i*_diazo_, describing the diazo ester, and an NBO point charge (*q*, Fig. 3c), describing electronic differences between 4-ethyl *versus* 4-isopropyl toluene substrates, stepwise linear regression was performed to identify the relationships given in Fig. 5. For both systems, *i*_diazo_ and the substrate point charge, *q*, combine to effectively describe the selectivity trends observed. The high degree of similarity between these models suggested that it might be possible to unite them into one mathematical equation. Thus, stepwise linear regression was performed on the 20 data points that result from the combination of both catalytic systems' DoE-defined training sets and the descriptive parameters *i*_diazo_, *q*, and a binary catalyst designation, *C* (Rh_2_(*S*-DOSP)_4_ = 1; Rh_2_(*R*–BPCP)_4_ = –1). The resulting comprehensive model and external validation-assessed (Tables 1, entries 11–16; 2, entries 11–19) robustness measure is given in Fig. 5c.^20^ This combined model effectively describes the system for both catalysts.

## Analysis

**Fig. 6:**
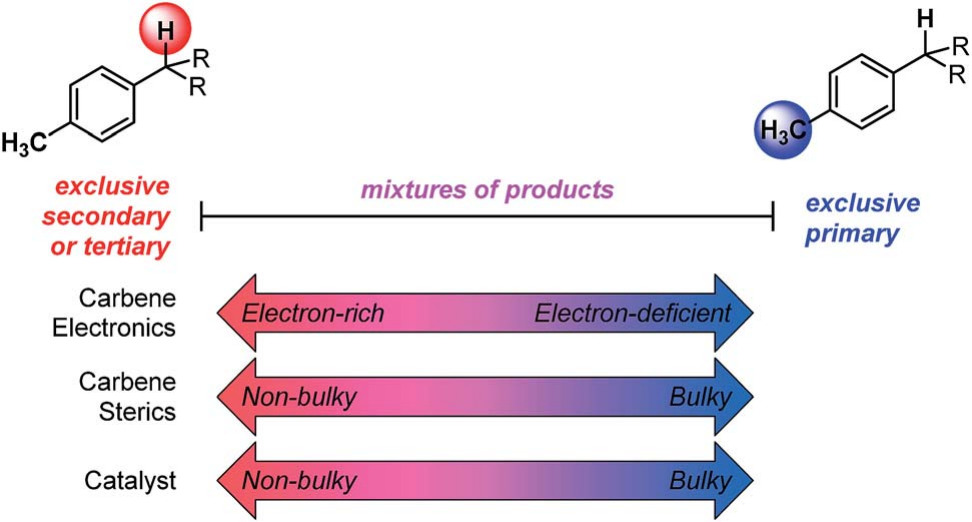
Spectrum of site-selectivity for primary *vs.* secondary/tertiary C–H functionalization. The effects of various features, such as steric and electronic influences of the carbene and the influence of the catalyst, are shown.

The developed combined model bears information that quantitatively illustrates the origin of selectivity in Rh(ii)-carbene C–H insertion reactions. The fact that both the Rh_2_(*R*-BPCP)_4_ (favoring primary) and Rh_2_(*S*-DOSP)_4_ (disfavoring primary) systems can be combined into one model demonstrates that both catalytic systems lie on a continuum with the same molecular features impacting selectivity (Fig. 6). While catalyst bias is the overriding selectivity determinant (which we surmise may be broadly attributable to steric differences), site selection is highly sensitive to the diazo reagent and substrate. Intuitively and irrespective of the catalyst, larger ester groups favor insertion at smaller primary C–H bonds. This is numerically evidenced in the interpretation of the *i*_diazo_ parameter. Greater substituent bulk, as defined with *L*_R′_, diminishes the intensity of the diazo stretch (Fig. 4a). As all three models in Fig. 5 indicate, lower values of *i*_diazo_ coincide with increased incidence of the primary insertion product. An explanation for these effects is offered: larger ester groups result in a bulkier carbene, which can better distinguish the more accessible primary C–H bonds from those at more sterically demanding secondary and tertiary positions.

Also represented in the *i*_diazo_ term is the electronic influence of the diazo, which originates in both the ester (R′) and aryl (R) substituents. From the model in Fig. 4a, it is demonstrated that more electron-deficient esters (lower p*K*_a_) diminish *i*_diazo_ and, correspondingly, erode the secondary (or tertiary)-to-primary product ratio (Fig. 5). Similarly, in the arene dimension (Fig. 4b), electron-withdrawing R substituents reduce *i*_diazo_ (*e.g.*, for R = CF_3_, *σ*^+^ = 0.61) and yield product ratios favoring insertion at primary C–H bonds. Taken together, these electronic effects can be explained by considering that electron-deficient groups will destabilize the electrophilic carbene, rendering it more reactive. As a result of its augmented reactivity, it proceeds with C–H insertion *via* the pathway leading to the kinetic product, *i.e.*, the more easily accessible, albeit stronger, primary C–H bond.

## Conclusions

In summary, the concept of having a toolbox of reagents/catalysts to control site selectivity by adjusting the carbene is exemplified in the ability to alter selectivity from a primary/tertiary selectivity ratio of 610 : 1 (Table 2, entry 10) to a primary/secondary ratio of 1 : 20 (Table 1, entry 1). The models developed demonstrate how these changes in selectivity ratios can be predictively afforded through a quantitative understanding of the systems' aspects that were investigated. Additionally, the use of correlative analysis to understand the origin of such disparate observations provides a platform for both prediction and design. Understanding such trends will be foundational in developing effective catalysts and reagents to control selective C–H functionalization, without relying on inherent substrate bias or directing groups to influence the site selectivity. Future experimental efforts are aimed at controlling the site-selectivity of C–H functionalization of unactivated hydrocarbons, where the concepts revealed within this study will play an important role in the design of effective systems.

## Supplementary Material

Supplementary information
