# Supplementary material for: Using IR vibrations to quantitatively describe and predict site-selectivity in multivariate Rh-catalyzed C–H functionalization
Source: Chem Sci. 2015 Mar 18;6(5):3057–62. doi: 10.1039/c5sc00357a (PMC5763982; doi:10.1039/c5sc00357a)
Supplement: Supplementary file 1 [file SC-006-C5SC00357A-s001.pdf]

Electronic Supplementary Material (ESI) for Chemical Science.
This journal is © The Royal Society of Chemistry 2015

Supporting Information for

Using IR Vibrations to Quantitatively Describe and Predict Site-Selectivity in Multivariate Rh-Catalyzed C–H Functionalization

Elizabeth N. Bess[a,‡], David M. Guptill[b,‡], Huw M. L. Davies[b,*], Matthew S. Sigman[a,*]

[a]Department of Chemistry, University of Utah, 315 South 1400 East, Salt Lake City, UT 84112, United States [b]Department of Chemistry, Emory University, 1515 Dickey Drive, Atlanta, Georgia 30322, United States

Contents

1 – General Considerations 2
2 – C–H Functionalization Reactions 3
2.1 General Procedure for C–H Functionalization Reactions 3
2.2 Procedure for Determining Product Ratios 4
3 – Preparation of Diazo Compounds 5
4 – Characterization Data for C–H Functionalization Products 16
4.1 Preparation and Characterization of Products from Functionalization of 4-ethyltoluene .. 16
Representative procedure for DCC coupling 19
4.2 Characterization of Products from Functionalization of 4-isopropyltoluene 32
5 – Supporting Information for Computational and Modeling Studies 39
6 – NMR Spectra 52

1 – General Considerations

All solvents were purified and dried by a *Glass Contour Solvent System* unless otherwise stated. The dichloromethane used for the carbenoid transformations was dried at reflux over 4Å molecular sieves for 3 hours under argon, and then distilled under argon before use. ^{1}H and ^{13}C NMR spectra were recorded at either 400 MHz (^{13}C at 100 MHz) or 600 MHz (^{13}C at 150 MHz) on an INOVA-400, Varian-400, or INOVA-600 spectrometer, as indicated. NMR spectra were run in solutions of deuterated chloroform ($CDCl_3$) with tetramethylsilane taken as an internal standard (0.0 ppm) for ^{1}H, and residual chloroform (77.23 ppm) for ^{13}C, and were reported in parts per million (ppm). Abbreviations for signal multiplicity are as follows: s = singlet, d = doublet, t = triplet, q = quartet, p = pentet, m = multiplet, dd = doublet of doublet, app t = apparent triplet, etc. Coupling constants (*J* values) were calculated directly from the spectra. IR spectra were collected on a Nicolet iS10 FT-IR spectrometer. Mass spectra were taken on a Thermo Finnigan LTQ-FTMS spectrometer with APCI, ESI or NSI. Thin layer chromatographic analysis was performed with glass-backed silica gel plates, visualizing with UV light and/or staining with PMA or aqueous $KMnO_4$ stain.

2 – C–H Functionalization Reactions

2.1 General Procedure for C–H Functionalization Reactions

For reactions with Rh_2(*S*-DOSP)$_4$, the solvent was 2,2-dimethylbutane. For reactions with Rh_2(*R*-BPCP)$_4$, the solvent was DCM.

A 10 mL, oven-dried round-bottomed flask, equipped with an egg shaped magnetic stir bar and a reflux condenser, was allowed to cool to room temperature under a stream of argon. The flask was charged with the catalyst (For DOSP – 1 mol %, 3.7 mg; For BPCP – 0.5 mol %, 1.8 mg), the 4-substituted toluene substrate (0.6 mmol, 3.0 equiv.) and 1 mL of the appropriate solvent. This mixture was heated to reflux. Then the diazo (0.2 mmol, 1.0 equiv.) was dissolved in 2.5 mL of DCM and added slowly over 1.5 hours to the solution of catalyst and substrate at reflux. The reaction mixture was allowed to stir for 20-30 minutes at reflux before it was cooled to room temperature. The starting material was removed according to the following procedures:

For reactions conducted in 2,2-DMB with DOSP: once the reaction mixture had cooled to room temperature, it was directly transferred by pipet to a short (4-5 cm) silica gel column packed using hexanes. The column was flushed with hexanes (30-40 mL) to remove the excess substrate, and then the product collected by flushing the column with DCM (20 mL). The solvent was removed to give the crude material, free of the starting material.

For reactions conducted in DCM with BPCP: when the reaction mixture had cooled, the DCM was removed by rotary evaporation. The crude green oil was transferred using hexanes to a short (4-5 cm) silica gel column packed using hexanes. The column was flushed with hexanes (30-40 mL) to remove the excess substrate, and then the product was collected by flushing the

column with DCM (20 mL). The solvent was removed to give the crude material, free from the excess starting material.

Note: *Though yields were not calculated, the C–H functionalization products were the major products, and it is expected that yields would be good (70-85%) for the majority of these reactions.*

2.2 Procedure for Determining Product Ratios

To measure the product ratios, the mixtures obtained as described in section 2.1 were analyzed by ^{1}H NMR using the following settings:

- Instrument: 600 MHz INOVA equipped with an ID probe with sensitivity of 1000:1
- Number of scans: 32
- Relaxation time: 3 seconds (essentially no difference in the measured ratio was observed with relaxation times up to 10 seconds)

The data was processed using MestReNova software, applying an auto-phase correction as well as a Whittaker Smoother baseline correction. The baseline was manually inspected before integration. The ratios were measured by integration of the NMR peaks resulting from the indicated hydrogens below.

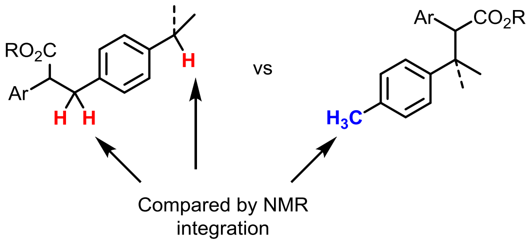

3 – Preparation of Diazo Compounds

Diazo compounds were prepared by direct diazo transfer to the methylene compounds as per the following scheme.

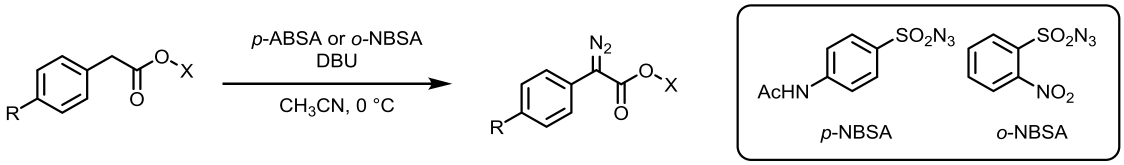

Methyl 4-*tert*-butylphenyldiazoacetate, methyl 4-bromophenyldiazoacetate, methyl 4-(trifluoromethyl)phenyldiazoacetate, methyl 4-chlorophenyldiazoacetate, and methyl 4-methoxyphenyldiazoacetate were prepared using *p*-ABSA, according to a literature procedure.[1] The reagent *o*-NBSA was prepared according to a literature protocol.[2] Other diazo compounds were prepared as described below.

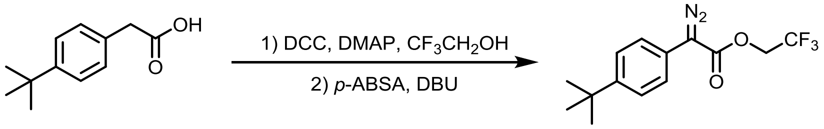

2,2,2-trifluoroethyl 2-(4-(*tert*-butyl)phenyl)-2-diazoacetate: 4-*tert*-butylphenylacetic acid (7.0 g, 36.4 mmol, 1.0 equiv.) was dissolved in 75 mL of 2,2,2-trifluoroethanol and 10 drops of concentrated sulfuric acid were added. The mixture was heated to reflux for 5 hours and then allowed to cool to room temperature. The reaction was quenched with saturated aqueous $NaHCO_3$ (75 mL) and Et_2O (100 mL). The organic layer was washed with saturated aqueous $NaHCO_3$, H_2O, and brine, and dried over $MgSO_4$. The solution was concentrated to give a colorless oil that was purified by column chromatography (5% Et_2O in pentane). The ester product (5.8 g, 21.1 mmol, 1.0 equiv.) was dissolved in 100 mL acetonitrile with *p*-ABSA (31.7

(1) Davies, H. M. L.; Hansen, T.; Churchill, M. R. *J. Am. Chem. Soc.* **2000**, *122*, 3063–3070.
(2) Brodsky, B. H.; Bois, J. Du. *Org. Lett.* **2004**, *6*, 2619–2621

mmol, 1.5 equiv.) and the solution cooled to 0 °C. Then DBU (6.3 mL, 42.2 mmol, 2.0 equiv.) was added drop-wise and the reaction stirred for 6 hours. The reaction mixture was quenched with saturated aqueous NH_4Cl (100 mL) and H_2O (25 mL) and extracted with Et_2O (150 mL). The organic layer was washed with brine and dried over $MgSO_4$ and concentrated. The crude material was purified by column chromatography (2% Et_2O in pentane) to give the diazo as a red oil that solidified in the freezer (-25 °C) (585 mg, 9% yield). *Note: The yield of this diazo is low presumably because p-ABSA was used as the diazo transfer reagent. It is expected the yield would be significantly improved by the use of o-NBSA.* ^{1}H NMR (600 MHz; $CDCl_3$) δ 7.44 (d, 2H, *J* = 8.5 Hz), 7.39 (d, 2H, *J* = 8.5 Hz), 4.65 (q, 2H, *J* = 8.4 Hz), 1.32 (s, 9H); ^{13}C NMR (150 MHz, $CDCl_3$) δ 163.5, 149.8, 126.2, 124.2, 123.0 (q, *J* = 277.9 Hz), 121.1, 60.3 (q, *J* = 36.8 Hz), 34.6, 31.3 (the resonance resulting from the diazo carbon was not detected); IR (film): 2966, 2906, 2872, 2091, 1716 cm^{-1}; HRMS (NSI) *m/z*: $[M+Na]^+$ calcd for $C_{14}H_{15}O_2N_2F_3Na$ 323.0978; found 323.0979;

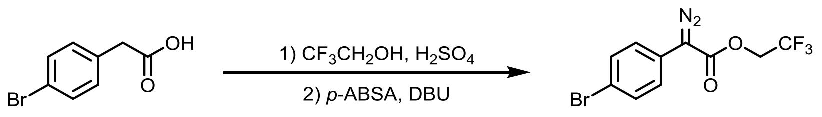

2,2,2-trifluoroethyl 2-(4-bromophenyl)-2-diazoacetate: The carboxylic acid (10.0 g, 46.5 mmol, 1.0 equiv.) was dissolved in 2,2,2-trifluoroethanol (100 mL) and several drops of concentrated sulfuric acid were added. The mixture was heated to reflux for 4 hours and then cooled to room temperature. Anhydrous potassium carbonate was added and the mixture stirred for 10 minutes. Solution filtered and concentrated, dissolved in Et_2O (100 mL), dried over $MgSO_4$, and concentrated, giving the product as a colorless oil (10.2 g, 74% yield). This was used immediately without further purification:

The ester from the previous step (2.0 g, 6.7 mmol, 1.0 equiv.) and *p*-acetamidobenzenesulfonyl azide (*p*-ABSA) (2.4 g, 10.1 mmol, 1.5 equiv.) were dissolved in acetonitrile (15 mL) and cooled to 0 °C. DBU (2.0 mL, 13.4 mmol, 2.0 equiv.) was added dropwise and the mixture was stirred overnight. The solution was quenched by addition of saturated aqueous ammonium chloride (20 mL) and the mixture was extracted with Et_2O (25 mL). The organic layer was dried over $MgSO_4$ and concentrated. The crude residue was purified by column chromatography to give the product as a fluffy yellow solid. (2.0 g, 91% yield). ^{1}H NMR (400 MHz; $CDCl_3$) δ 7.56-7.51 (m, 2H), 7.38-7.33 (m, 2H), 4.66 (q, 2H, J = 8.3 Hz); ^{13}C NMR (100 MHz; $CDCl_3$) δ IR (neat): 2973, 2092, 1698, 1140 cm^{-1}; HRMS (NSI) *m/z*: $[M]^+$ calcd for $C_{10}H_6O_2N_2F_3Br$ 321.9559, found 321.9563;

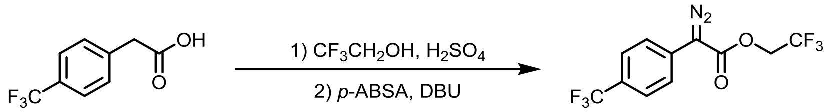

2,2,2-trifluoroethyl 2-diazo-2-(4-(trifluoromethyl)phenyl)acetate: 4-(trifluoromethyl)phenylacetic acid (8.0 g, 39.2 mmol, 1.0 equiv.) was dissolved in 75 mL of 2,2,2-trifluoroethanol. 10 drops of concentrated sulfuric acid was added and the mixture heated to reflux for 5 hours. It was allowed to cool to room temperature and quenched by addition of saturated $NaHCO_3$ (150 mL). The solution was extracted with Et_2O (100 mL). The organic layer was washed with saturated aqueous $NaHCO_3$, H_2O, and brine, and dried over $MgSO_4$ and concentrated. This gave a crude, waxy white solid, which was purified by column chromatography (5% Et_2O in pentane). A portion of the purified material (2.2 g, 7.7 mmol, 1.0 equiv.) was dissolved in acetonitrile (50 mL) together with *p*-ABSA (2.8 g, 11.5 mmol, 1.5 equiv.) and the solution cooled to 0 °C. Then DBU (2.3 mL, 15.4 mmol, 2.0 equiv.) was added

drop-wise. The solution was stirred for 6 hours and quenched with saturated aqueous NH_4Cl (75 mL) and H_2O (50 mL). The solution was extracted with Et_2O (100 mL) and the organic layer separated, dried over $MgSO_4$, and concentrated. The crude material was purified by column chromatography (2% Et_2O in pentane) to give the diazo as a yellow solid (1.3 g, 54% yield). 1H NMR (600 MHz; $CDCl_3$) δ 7.64 (d, 2H, J = 8.5 Hz), 7.59 (d, 2H, J = 8.5 Hz), 4.67 (d, 2H, J = 8.3 Hz); ^{13}C NMR (150 MHz, $CDCl_3$) δ 162.5, 129.1, 128.2 (q, J = 32.9 Hz), 126.0 (q, J = 3.5 Hz), 124.0 (q, J = 271.7 Hz), 123.5, 122.9 (q, J = 277.4 Hz), 60.5 (q, J = 37.1 Hz) (Note: the resonance resulting from the diazo carbon was not detected); IR (neat): 2978, 2097, 1716, 1075 cm^{-1}; HRMS (NSI) *m/z*: $[M+H]^+$ calcd for $C_{11}H_7F_6N_2O_2$ 313.0406; found 313.0409;

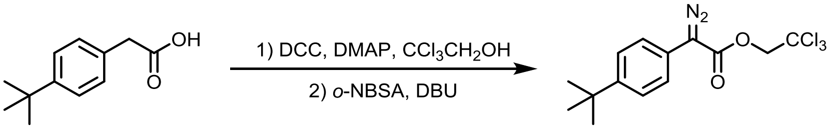

2,2,2-trichloroethyl 2-(4-(*tert*-butyl)phenyl)-2-diazoacetate: A solution of 4-*tert*-butylphenylacetic acid (5.0 g, 26.0 mmol, 1.0 equiv.), 2,2,2-trichloroethanol (4.6 g, 3.0 mL, 31.2 mmol, 1.2 equiv.) and DMAP (317 mg, 2.6 mmol, 0.1 equiv.), in 50 mL CH_2Cl_2 was cooled to 0 °C in an ice/water bath. Then DCC (5.9 g, 28.6 mmol, 1.1 equiv.), in 15 mL CH_2Cl_2 was poured into the cold reaction mixture. The solution was allowed to stir overnight, at which point it had reached ambient temperature. The precipitate was removed by vacuum filtration, washing once with Et_2O (20 mL). The filtrate was concentrated to give a crude oil. This was dissolved in pentane and added to a column loaded with 100 mL silica gel, packed with 1% Et_2O in pentane, and eluted with the same. The product was isolated as a crystalline white solid. (8.1 g, 96% yield). This was used immediately in the next step:

The ester (5.0 g, 15.5. mmol, 1.0 equiv.) and *o*-NBSA (5.3 g, 23.2 mmol, 1.5 equiv.) were dissolved in acetonitrile (100 mL) and the solution cooled to 0 °C. DBU (5.2 g, 5.1 mL, 34.1 mmol, 2.2 equiv.) was added dropwise. The solution was stirred 4 hours and quenched by addition of saturate aqueous NH_4Cl (50 mL) and water (20 mL). The mixture was extracted with Et_2O (200 mL). The mixture was washed with water (100 mL) and brine (100 mL), and dried over $MgSO_4$. The solution was concentrated to give a crude red oil. This was purified by a short column, using 2% and then 4% Et_2O in pentane. The product was isolated as an orange solid. ^1H NMR (600 MHz; $CDCl_3$) δ 7.47-7.40 (m, 4H), 4.91 (s, 2H), 1.32 (s, 9H); ^{13}C NMR (150 MHz, $CDCl_3$ with 2-3 mg $Cr(acac)_3$) δ 163.7, 149.7, 126.2, 124.2, 121.4, 95.2, 73.9, 34.6, 31.4 (the resonance resulting from the diazo carbon was not detected); IR (film): 2961, 2904, 2868, 2088, 1712 cm^{-1}; HRMS (APCI) *m/z*: $[M+H-N_2]^+$ calcd for $C_{14}H_{16}O_2Cl_3$ 321.0210; found 321.0206;

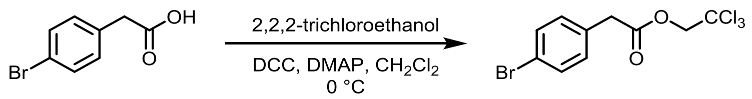

2,2,2-trichloroethyl 2-(4-bromophenyl)acetate: 4-bromophenylacetic acid (30.0 g, 140 mmol, 1.0 equiv.), DMAP (1.7 g, 14 mmol, 0.1 equiv.), and 2,2,2-trichloroethanol (25.1 g, 16 mL, 168 mmol, 1.2 equiv.) were dissolved in 300 mL CH_2Cl_2 and the solution cooled to 0 °C. A solution of DCC (31.7 g, 153 mmol, 1.1 equiv.) in CH_2Cl_2 (150 mL) was poured slowly into the cold reaction mixture. The reaction mixture was allowed to stir overnight, at which point it had warmed to room temperature. The solids were removed by suction filtration, and washed with Et_2O (100 mL). The filtrate was concentrated to give a yellow oil that solidified under high vacuum. The solid mass was broken up with a spatula and 150 mL hexanes was added to the flask. The mixture was heated until all but a small amount of white powder remained undissolved. The hot solution was filtered and cooled to room temperature before it was placed

in a freezer (-25 °C) overnight. The crystals that had formed were collected by vacuum filtration, washing once with hexanes (100 mL). The product was dried by suction on the frit used for filtration to give a white crystalline solid (38.8 g, 80% yield). This was used without further purification in the diazo transfer reaction.

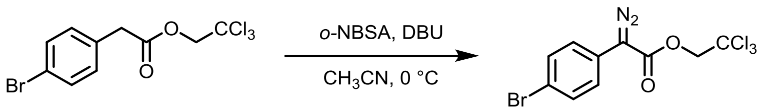

2,2,2-trichloroethyl 2-(4-bromophenyl)-2-diazoacetate: The ester from the previous step (10.0 g, 28.9 mmol, 1.0 equiv.), together with *o*-NBSA (9.9 g, 43.4 mmol, 1.5 equiv.), were dissolved in acetonitrile (100 mL) and the solution cooled to 0 °C. DBU (9.5 mL, 63.6 mmol, 2.2 equiv.) was added dropwise via syringe. The solution was stirred for 1 hour at 0 °C before it was quenched with saturated aqueous NH_4Cl (100 mL). The mixture was extracted with Et_2O (200 mL). The organic layer was washed with H_2O (100 mL) and brine (100 mL), dried over $MgSO_4$ and filtered. To the filtrate was added 50 g of silica gel and the solvent removed *in vacuo*. The dry silica was transferred to a column (containing 500 mL silica gel, packed with 2% Et_2O in pentane) using the same solvent. The column was eluted with 2% Et_2O in pentane, and the orange fractions were collected and combined. The solvent was removed on a rotovap below room temperature (keeping the flask out of the water bath), giving the product as an orange crystalline solid (9.3 g, 86% yield). ^{1}H NMR (400 MHz; $CDCl_3$) δ 7.57-7.51 (m, 2H), 7.42-7.36 (m, 2H), 4.92 (s, 2H); ^{13}C NMR (150 MHz, $CDCl_3$) δ 163.1, 132.4, 125.6, 124, 120.1, 95.1, 74.1 (t, *J* = 16.4 Hz) (note: the resonance resulting from the diazo carbon was not observed); IR (film): 2953, 2089, 1709, 1490 cm^{-1};

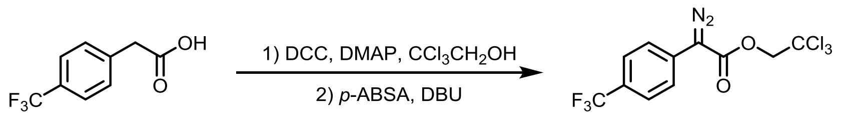

2,2,2-trichloroethyl 2-diazo-2-(4-(trifluoromethyl)phenyl)acetate: A solution of 4-(trifluoromethyl)phenylacetic acid (10.0 g, 49 mmol, 1.0 equiv.), 2,2,2-trichloroethanol (8.8 g, 5.6 mL, 58.8 mmol, 1.2 equiv.) and DMAP (599 mg, 4.9 mmol, 0.1 equiv.) in CH_2Cl_2 (100 mL) was cooled to 0 °C in an ice/water bath. A solution of DCC (11.1 g, 53.9 mmol, 1.1 equiv.) in CH_2Cl_2 (25 mL) was poured into the cold reaction mixture. The solution was stirred overnight, at which point it had reached ambient temperature. The precipitate was filtered and washed with Et_2O. The filtrate was concentrated to give a crude oil, which was purified by column chromatography (2% Et_2O in pentane), to give the product as a colorless oil (14.0 g, 85% yield). This was used immediately in the next step:

The ester from the previous step (5.0 g, 14.9 mmol, 1.0 equiv.) and *p*-ABSA (5.4 g, 22.4 mmol, 1.5 equiv.) were dissolved in acetonitrile (100 mL) and cooled to 0 °C. Then DBU (4.5 g, 4.4 mL, 29.8 mmol, 2.0 equiv.) was added dropwise. The solution was stirred 1.5 hours and quenched with saturated aqueous NH_4Cl (75 mL) and water (20 mL) and extracted with Et_2O (100 mL). The organic layer was washed with water (50 mL) and brine (50 mL) and dried over $MgSO_4$. The solution was concentrated to give a crude oil. This was purified by column chromatography (2% Et_2O in pentane) to give the product as a yellow oil that solidified upon standing (2.0 g, 37% yield). ^{1}H NMR (600 MHz; $CDCl_3$) δ 7.65 (d, 2H, J = 8.9 Hz), 7.63 (d, 2H, J = 8.9 Hz), 4.93 (s, 2H); ^{13}C NMR (100 MHz, $CDCl_3$ with 2-3 mg Cr(acac)$_3$) δ 162.5, 129.2, 128 (q, J = 32.8 Hz), 125.9 (q, J = 3.5 Hz), 123.9 (q, J = 271.9), 123.5, 94.8, 73.9, 64.1; IR (neat): 2962, 2091, 1716, 1325 cm^{-1}; HRMS (ESI) *m/z*: $[2M+H-N_2]^+$ calcd for $C_{22}H_{13}O_4N_2Cl_6F_6$ 692.8905, found 692.8898;

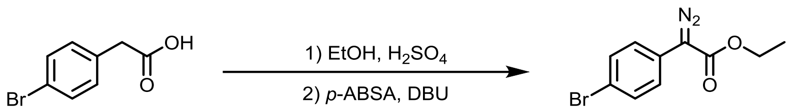

ethyl 2-(4-bromophenyl)-2-diazoacetate: The ethyl ester was prepared by stirring 4-bromophenylacetic acid with catalytic sulfuric acid in ethanol for 24 hours. The ester prepared in this manner (10.8 g, 44.4 mmol, 1.0 equiv.) and *p*-ABSA (16 g, 66.6 mmol, 1.5 equiv.) were dissolved in CH_3CN (100 mL) and the solution cooled to room temperature. DBU (13.3 mL, 88.8 mmol, 2.0 equiv.) was added drop-wise. The reaction was stirred for 5 hours and quenched with aqueous NH_4Cl (75 mL) and H_2O (25 ml). The mixture was extracted with Et_2O (150 mL). The organics were washed with brine, dried over $MgSO_4$ and concentrated. The crude material was purified by column chromatography (5% Et_2O in pentane) to give the diazo as an orange crystalline solid (11.2 g, 94% yield). The spectral data were consistent with those reported in the literature.[3]

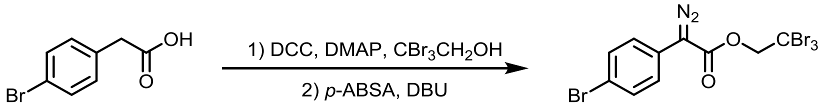

2,2,2-tribromoethyl 2-(4-bromophenyl)-2-diazoacetate: 4-bromophenylacetic acid (10 g, 46.5 mmol, 1.0 equiv.), DMAP (567 mg, 4.65 mmol, 0.1 equiv.) and 2,2,2-tribromoethanol (15.8 g, 55.8 mmol, 1.2 equiv.) were dissolved in DCM (75 mL) and the solution cooled to 0 °C. A solution of DCC (10.5 g, 51.2 mmol, 1.1 equiv.) in DCM (25 mL) was poured into the reaction. The mixture was stirred overnight, after which time it had warmed to room temperature. The precipitate was removed by vacuum filtration, washing the solids with Et_2O. The filtrate was concentrated to give a crude white solid. This was dissolved in approximately 600 mL of boiling hexanes, and the remaining undissolved solid removed by filtration while the solution was hot.

(3) Hahn, N. D.; Nieger, M.; Dötz, K. H. *J. Organomet. Chem.* **2004**, *689*, 2662–2673

The hot filtrate was placed in an ice bath for 20 minutes, and then a freezer (-25 °C) for 2 hours. The crystals were collected by vacuum filtration, washing once with cold hexanes, and then dried under vacuum. (16.6 g, 74% yield). A portion of this ester product (3.6 g, 7.5 mmol, 1.0 equiv.), together with *o*-NBSA (2.6 g, 11.3 mmol, 1.5 equiv.) was suspended in acetonitrile (100 mL) and cooled to 0 °C. DBU (2.5 mL, 16.5 mmol, 2.2 equiv.) was added and the solution stirred for 1 hour. Reaction was quenched with saturated aqueous NH_4Cl (100 ml) and extracted with Et_2O (75 mL). The organic layer was washed with water and brine, dried over $MgSO_4$, and concentrated. The crude mixture was purified by column chromatography (1.5% Et_2O in pentane), giving the diazo as an orange crystalline solid (1.9 g, 50% yield). ^{1}H NMR (600 MHz; $CDCl_3$) δ 7.54-7.51 (m, 2H), 7.41-7.38 (m, 2H), 5.09 (s, 2H); ^{13}C NMR (150 MHz, $CDCl_3$) δ 162.8, 132.2, 125.5, 124.0, 119.9, 76.8, 35.8 (the resonance resulting from the diazo carbon was not detected); IR (neat): 2950, 2091, 1701, 1140 cm^{-1}; HRMS (NSI) *m/z*: $[M+H-N_2]^+$ calcd for $C_{10}H_7O_2Br_4$ 490.7123; found 490.7132;

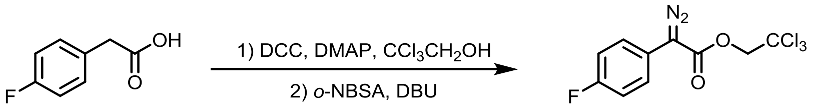

2,2,2-trichloroethyl 2-diazo-2-(4-fluorophenyl)acetate: A solution of 4-fluorophenylacetic acid (10.0 g, 64.9 mmol, 1.0 equiv.), 2,2,2-trichloroethanol (11.6 g, 7.5 mL, 77.9 mmol, 1.2 equiv.) and DMAP (793 mg, 6.5 mmol, 0.1 equiv.) in CH_2Cl_2 (150 mL) was cooled to 0 °C. A solution of DCC in CH_2Cl_2 (45 mL) was poured into the cold reaction mixture. The solution was stirred overnight, at which point it had reached ambient temperature. The precipitate was removed by filtration and washed with Et_2O. The filtrate was concentrated and purified by

column chromatography (short silica plug, 5% Et_2O in pentane). The product was isolated as a colorless oil (15.1 g, 82% yield). This was used immediately in the next step:

The ester from the previous step (5.0 g, 17.5 mmol, 1.0 equiv.) and *o*-NBSA (6.0 g, 26.3 mmol. 1.5 equiv.) were dissolved in acetonitrile (75 mL) and cooled to 0 °C. Then DBU (5.6 g, 5.5 mL, 38.5 mmol, 2.2 equiv.) was added dropwise. The solution was stirred for 2 hours and quenched with water (75 mL). The solution was extracted with pentane until the extracts were no longer yellow (required about 3 x 150 mL). These extracts were combined and poured directly onto a silica gel column and the pentane eluted with pressure until the solution reached the top of the silica. The column was then eluted with 1% Et_2O in pentane. The yellow fractions were collected and concentrated (below room temperature) to give the product as a crystalline orange solid (5.0 g, 91% yield). ^{1}H NMR (400 MHz; $CDCl_3$) δ 7.50-7.43 (m, 2H), 7.15-7.08 (m, 2H), 4.90 (s, 2H); ^{13}C NMR (150 MHz, $CDCl_3$, with 2-3 mg of $Cr(acac)_3$) δ 163.4, 162.2 (d, *J* = 247.1 Hz), 126.1, 120.4 (d, *J* = 2.7 Hz), 116.3 (d, *J* = 21.9 Hz), 95.1, 73.9 (The resonance resulting from the diazo carbon was not observed); IR (neat): 2954, 2093, 1690, 1507 cm^{-1}; HRMS (APCI) *m/z*: $[M+H-N_2]^+$ calcd for $C_{10}H_7O_2Cl_3F$ 282.9490; found 282.9487; Spectral data consistent with those previously reported.[4]

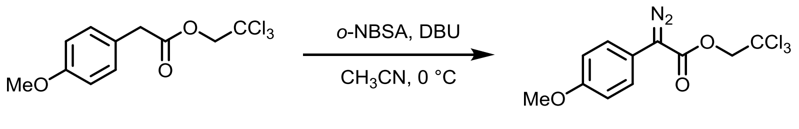

2,2,2-trichloroethyl 2-diazo-2-(4-methoxyphenyl)acetate: Prepared by using *o*-NBSA and DBU in a manner analogous to the previous example (2,2,2-trichloroethyl 2-diazo-2-(4-fluorophenyl)acetate). Characterization data: ^{1}H NMR (600 MHz, $CDCl_3$) d 7.38 (d, 2H, *J* = 8.7 Hz), 6.94 (d, 2H, *J* = 8.7 Hz), 4.88 (s, 2H), 3.08 (s, 3H); ^{13}C NMR (150 MHz, $CDCl_3$) 164.0,

(4) Guptill, D. M.; Davies, H. M. L. *J. Am. Chem. Soc.* **2014,** *accepted*, doi: 10.1021/ja5107404.

158.6, 126.3, 116.2, 114.9, 95.3, 74.0, 55.6; IR (neat): 2953, 2935, 2090, 1690, 1234 cm^{-1}; HRMS (APCI) *m/z*: $[M+H-N_2]^+$ calcd for $C_{11}H_{10}O_3Cl_3$ 294.9690, found 294.9688;

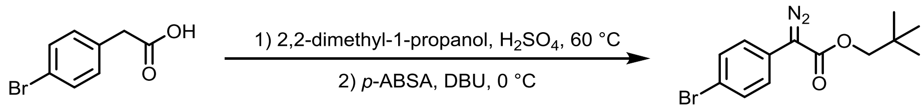

neopentyl 2-(4-bromophenyl)-2-diazoacetate: The carboxylic acid (5.0 g, 23.3 mmol, 1.0 equiv.) and neopentyl alcohol (20 mL) were added to a flask. The mixture was placed in a 60 °C oil bath until the solids melted to form a homogenous solution. Concentrated sulfuric acid (several drops) was added and the mixture stirred at 60 °C overnight. The reaction was quenched by addition of saturated aqueous sodium bicarbonate (50 mL). The biphasic mixture was extracted with Et_2O (50 mL). The ethereal layer was washed with saturated aqueous sodium bicarbonate, water and brine (50 mL each), dried over $MgSO_4$, and concentrated by rotary evaporation (bath set at 50 °C to remove excess alcohol). This gave the desired product as a colorless oil (6.4 g, 97% yield). This was used immediately in the next reaction without further purification:

The ester from the previous step (6.4 g, 22.5 mmol, 1.0 equiv.), and *p*-ABSA (8.1 g, 33.7 mmol, 1.5 equiv.) were dissolved in acetonitrile (100 mL) and the solution cooled to 0 °C. DBU (6.7 mL, 45 mmol, 2.0 equiv.) was added dropwise and the solution stirred for 3 days, and quenched with saturated aqueous ammonium chloride (75 mL) and water (25 mL). The mixture was extracted with Et_2O (100 mL) and the organic layer was separated, washed with brine (50 mL) and dried over $MgSO_4$ and concentrated. The crude orange powder was dissolved in pentane and loaded onto a short silica gel column, eluting with 5% Et_2O in pentane. The orange fractions were collected and concentrated to give the diazo as an orange solid (5.5 g, 79% yield). ^1H NMR

(600 MHz; $CDCl_3$) δ 7.53-7.48 (m, 2H), 7.40-7.36 (m, 2H), 3.97 (s, 2H), 0.98 (s, 9H); ^{13}C NMR (150 MHz, $CDCl_3$) δ 165.0, 132.2, 125.5, 125.0, 119.4, 74.5, 31.7, 26.6 (the resonance resulting from the diazo carbon was not detected); IR (neat): 2962, 2867, 2083, 1694, 1161 cm^{-1}; HRMS (NSI) *m/z*: $[M+Na]^+$ calcd for $C_{13}H_{15}O_2N_2BrNa$ 333.0209, found 333.021;

4 – Characterization Data for C–H Functionalization Products

4.1 Preparation and Characterization of Products from Functionalization of 4-ethyltoluene

The mixtures of products formed from reaction with 4-ethyltoluene were generally inseparable by chromatography. Therefore, authentic samples of the products from functionalization at the primary and secondary positions were prepared independently by an alternative method for characterization. Products from functionalization at the primary position were prepared as follows:

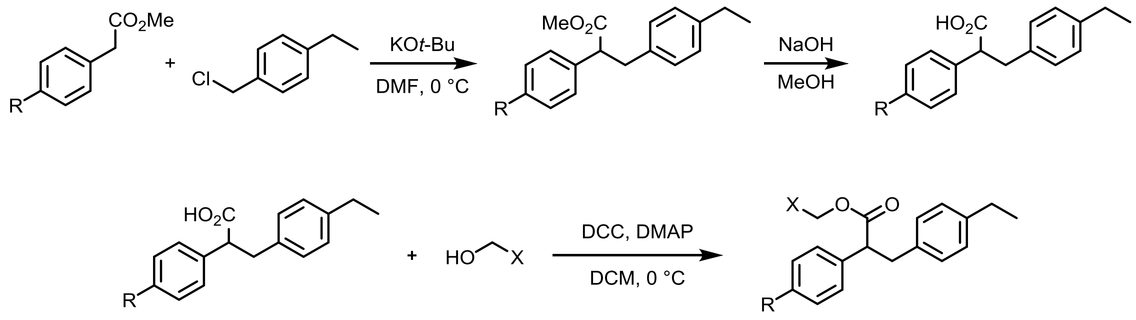

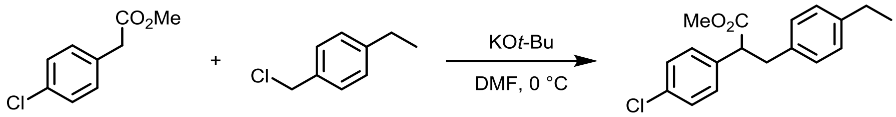

methyl 2-(4-chlorophenyl)-3-(4-ethylphenyl)propanoate: The ester compound (2.4 g, 12.8 mmol, 4.0 equiv.) and 4-ethylbenzyl chloride (500 mg, 3.2 mmol, 1.0 equiv.) were dissolved in DMF (10 mL) and cooled to 0 °C. Solid potassium *tert*-butoxide (393 mg, 3.5 mmol, 1.1 equiv.) was added in one portion. The solution was stirred for 10 minutes and then quenched with saturated aqueous NH_4Cl (5 mL) and diluted with H_2O (25mL). The mixture was extracted with hexanes (25 mL). The organics were dried over $MgSO_4$ and concentrated. Purified by column chromatography (0.75% Et_2O in pentane). The purest 2-3 fractions from the column were combined and concentrated for characterization. ^1H NMR (600 MHz; $CDCl_3$) δ 7.28-7.24 (m, 2H), 7.24-7.20 (m, 2H), 7.06 (d, 2H, *J* = 8.2 Hz), 7.00 (d, 2H, *J* = 8.2 Hz), 3.81 (dd, 1H, *J* = 8.4, 7.2 Hz), 3.60 (s, 3H), 3.35 (dd, 1H, *J* = 13.8, 8.4 Hz), 2.96 (dd, 1H, *J* = 13.8, 7.2 Hz), 2.58 (q, 2H, *J* = 7.6 Hz), 1.19 (t, 3H, *J* = 7.6 Hz); ^{13}C NMR (150 MHz, $CDCl_3$) δ 173.7, 142.6, 137.3, 135.9, 133.4, 129.6, 129.0, 128.9, 128.1, 53.2, 52.3, 39.5, 28.6, 15.7; IR (film): 3008, 2962, 2930, 1734 cm^{-1}; HRMS (NSI) *m/z*: $[M+Na]^+$ calcd for $C_{18}H_{19}O_2ClNa$ 325.0966; found 325.0975;

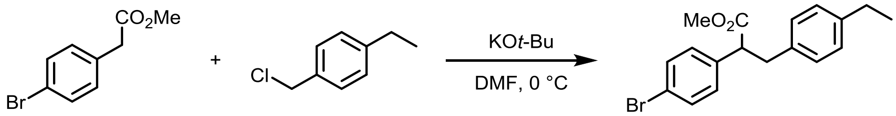

methyl 2-(4-bromophenyl)-3-(4-ethylphenyl)propanoate: 4-ethylbenzyl chloride (4.1g, 26.2 mmol, 1.2 equiv.) and methyl 4-bromophenylacetate (5.0g, 21.8 mmol, 1.0 equiv.) were dissolved in 50 mL DMF and cooled to 0 °C. Solid potassium *tert*-butoxide (2.9 g, 26.2 mmol, 1.2 equiv.) was added in one portion. The solution was stirred overnight, after which time it had

warmed to room temperature. The reaction was quenched by the addition of H_2O (50 mL) and was extracted with Et_2O (75 mL). The organic layer was washed with H_2O (50 mL) and dried over $MgSO_4$, and concentrated. The crude mixture was purified by column chromatography (1% →2% Et_2O in pentane). The product was isolated as a colorless oil, but was not completely pure (4.4g, ~58% yield). This was used in the next step without further purification.

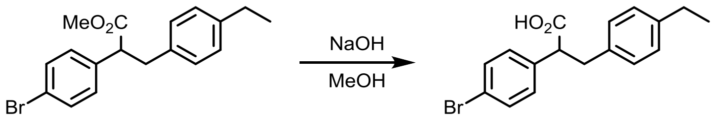

2-(4-bromophenyl)-3-(4-ethylphenyl)propanoic acid: The ester (4.4g, 12.7 mmol, 1.0 equiv.) was dissolved in MeOH (200 mL) and a solution of NaOH (19 mL, 1 M in H_2O, 10 mmol, 1.5 equiv.) was added. The solution was stirred at room temperature overnight. The mixture was diluted with H_2O (200 mL) and extracted with Et_2O (100 mL). The aqueous phase was acidified to pH = 1.0 with concentrated HCl. The solution was then extracted with Et_2O (2 x 100 mL). The organics were dried ($MgSO_4$) and concentrated. The crude material was purified by column chromatography (4:1 hexanes:EtOAc) to give the product as an off-white solid (2.5 g, 60% yield). ^{1}H NMR (600 MHz; $CDCl_3$) δ 7.44 (d, 2H, *J* = 8.4 Hz), 7.18 (d, 2H, *J* = 8.4 Hz), 7.06 (d, 2H, *J* = 8.0 Hz), 7.00 (d, 2H, *J* = 8.0 Hz), 3.81 (app t, 1H, *J* = 7.7 Hz), 3.36 (dd, 1H, *J* = 13.9, 8.0 Hz), 2.98 (dd, 1H, *J* = 13.9, 7.3 Hz), 2.60 (q, 2H, *J* = 7.6 Hz), 1.21 (t, 3H, *J* = 7.6 Hz); ^{13}C NMR (150 MHz, $CDCl_3$) δ 179.1, 142.6, 136.9, 135.3, 131.8, 129.9, 128.8, 128.0, 121.7, 52.9, 38.7, 28.5, 15.5; IR (neat): 3030, 2977, 2931, 2904, 1706 cm^{-1}; HRMS (NSI) *m/z*: $[M+H]^+$ calcd for $C_{17}H_{18}O_2Br$ 333.0485; found 333.0487;

Representative procedure for DCC coupling

The following procedure for DCC coupling is representative:

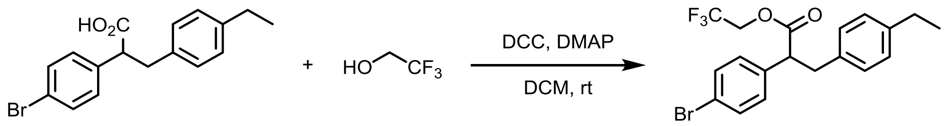

2,2,2-trifluoroethyl 2-(4-bromophenyl)-3-(4-ethylphenyl)propanoate: The carboxylic acid (100 mg, 0.3 mmol, 1.0 equiv.), DMAP (4 mg, 0.03 mmol, 0.10 equiv.) and 2,2,2-trifluoroethanol (36 mg, 0.36 mmol, 1.2 equiv.) were added to a 4 mL glass screw-cap vial equipped with a micro stir bar and dissolved in 1 mL DCM. The solution was stirred until the solution became homogenous. Then a solution of DCC (68 mg, 0.33 mmol, 1.1 equiv.) in DCM (1 mL) was added in one portion by pipet. The solution was stirred at room temperature overnight. The mixture was filtered through a pad of celite in a pipet, and the filter cake washed with pentane. The filtrate was concentrated to give a crude oil that was purified by column chromatography (1% Et_2O in pentane). The purest, first two fractions, containing the product were combined and concentrated to give a pure sample for characterization. ^{1}H NMR (600 MHz; $CDCl_3$) δ 7.46 (d, 2H, *J* = 8.4 Hz), 7.20 (d, 2H, *J* = 8.4 Hz), 7.09 (d, 2H, *J* = 8.0 Hz), 7.03 (d, 2H, *J* = 8.0 Hz), 4.45-4.33 (m, 2H), 3.93 (dd, 1H, *J* = 8.7, 7.0 Hz), 3.38 (dd, 1H, *J* = 13.9, 8.7 Hz), 3.03 (dd, 1H, *J* = 13.9, 7.0 Hz), 2.61 (q, 2H, *J* = 7.6 Hz), 1.22 (t, 3H, *J* = 7.6 Hz); ^{13}C NMR (150 MHz, $CDCl_3$) δ 171.5, 142.7, 136.5, 135.0, 131.9, 129.7, 128.8, 128.0, 122.8 (q, *J* = 277.2 Hz), 121.8, 60.6 (q, *J* = 36.7 Hz), 52.7, 39.1, 28.5, 15.6; IR (neat): 2966, 2931, 2873, 1754 cm^{-1}; HRMS (NSI) *m/z*: $[M+Na]^+$ calcd for $C_{19}H_{18}BrF_3O_2$ 437.0335; found 437.0334;

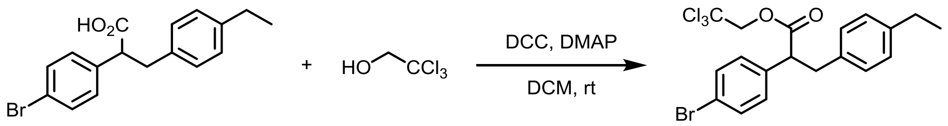

2,2,2-trichloroethyl 2-(4-bromophenyl)-3-(4-ethylphenyl)propanoate: Prepared according to the representative procedure for DCC coupling with the acid (100 mg), DMAP (4 mg), and DCC (68 mg), as well as 2,2,2-trichloroethanol (54 mg, 0.36 mmol, 1.2 equiv.). The product was purified by column chromatography (1% Et_2O in pentane), taking the purest few fractions for characterization of the product. ^{1}H NMR (600 MHz; $CDCl_3$) δ 7.46-7.43 (m, 2H), 7.25-7.22 (m, 2H), 7.07 (d, 2H, *J* = 8.3 Hz), 7.05 (d, 2H, *J* = 8.3 Hz), 4.67 (d, 1H, *J* = 12.0 Hz), 4.62 (d, 1H, *J* = 12.0 Hz), 3.96 (dd, 1H, *J* = 8.9, 6.8 Hz), 3.40 (dd, 1H, *J* = 13.9, 8.9 Hz), 3.04 (dd, 1H, *J* = 13.9, 6.8 Hz), 2.58 (q, 2H, *J* = 7.6 Hz), 1.19 (t, 3H, *J* = 7.6 Hz); ^{13}C NMR (150 MHz, $CDCl_3$) δ 171.5, 142.8, 136.8, 135.3, 132.0, 130.1, 129.0, 128.2, 121.9, 94.8, 74.3, 53.1, 39.1, 28.6, 15.8; IR (neat): 2962, 2929, 1749, 1133 cm^{-1}; HRMS (NSI) *m/z*: $[M+Na]^+$ calcd for $C_{19}H_{18}O_2BrCl_3Na$ 484.9448; found 484.9461; Data matched those previously reported.[5]

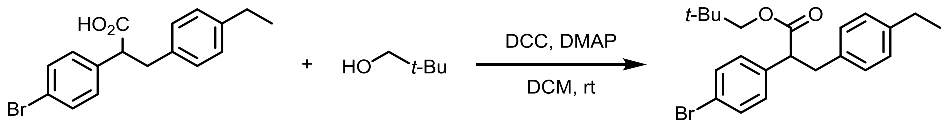

neopentyl 2-(4-bromophenyl)-3-(4-ethylphenyl)propanoate: Prepared according to the representative procedure for DCC coupling with the acid (100 mg), DMAP (4 mg), and DCC (68 mg), as well as neopentyl alcohol (26 mg, 0.36 mmol, 1.2 equiv.). The product was purified by column chromatography (1% Et_2O in pentane), taking the purest few fractions for characterization of the product. ^{1}H NMR (600 MHz; $CDCl_3$) δ 7.46-7.42 (m, 2H), 7.25-7.21 (m, 2H), 7.08 (d, 2H, *J* = 8.3 Hz), 7.05 (d, 2H, *J* = 8.3 Hz), 3.85 (dd, 1H, *J* = 8.7, 7.0 Hz), 3.72 (s,

(5) Guptill, D. M.; Davies, H. M. L. *J. Am. Chem. Soc.* **2014,** *accepted*, doi: 10.1021/ja5107404.

2H), 3.38 (dd, 1H, J = 13.8, 8.7 Hz), 3.01 (dd, 1H, J = 13.8, 7.0 Hz), 2.60 (q, 2H, J = 7.6 Hz), 1.21 (t, 3H, J = 7.6 Hz), 0.79 (s, 9H); ^{13}C NMR (150 MHz, $CDCl_3$) δ 173.0, 142.4, 137.8, 135.7, 131.6, 129.8, 128.8, 127.9, 121.2, 74.1, 53.3, 39.1, 31.3, 28.4, 26.3, 15.6; IR (film): 2959, 2869, 1732, 1151 cm^{-1}; HRMS (NSI) *m/z*: $[M+H]^+$ calcd for $C_{22}H_{28}O_2Br$ 403.1267; found 403.1271;

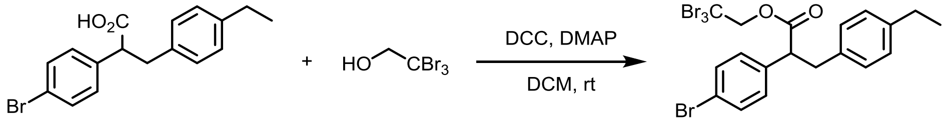

2,2,2-tribromoethyl 2-(4-bromophenyl)-3-(4-ethylphenyl)propanoate: Prepared according to the representative procedure for DCC coupling with the acid (100 mg), DMAP (4 mg), and DCC (68 mg), as well as 2,2,2-tribromoethanol (102 mg, 0.36 mmol, 1.2 equiv.). The product was purified by column chromatography (1% Et_2O in pentane), taking the purest few fractions for characterization of the product. ^{1}H NMR (600 MHz; $CDCl_3$) δ 7.46-7.43 (m, 2H), 7.29-7.25 (m, 2H), 7.07 (app s, 4H,), 4.87 (d, 1H, J = 12.2 Hz), 4.80 (d, 1H, J = 12.2 Hz), 3.98 (dd, 1H, J = 8.8, 6.8 Hz), 3.44 (dd, 1H, J = 13.9, 8.8 Hz), 3.06 (dd, 1H, J = 13.9, 6.8 Hz), 2.58 (q, 2H, J = 7.6 Hz), 1.19 (t, 3H, J = 7.6 Hz); ^{13}C NMR (150 MHz, $CDCl_3$) δ 171.3, 142.8, 136.9, 135.4, 132.0, 130.2, 129.1, 128.2, 121.9, 77.2, 53.3, 39.1, 35.4, 28.6, 15.8; IR (film): 3008, 2961, 2928, 1747 cm^{-1}; HRMS (NSI) *m/z*: $[M+H]^+$ calcd for $C_{19}H_{19}O_2Br_4$ 594.8113; found 594.8130;

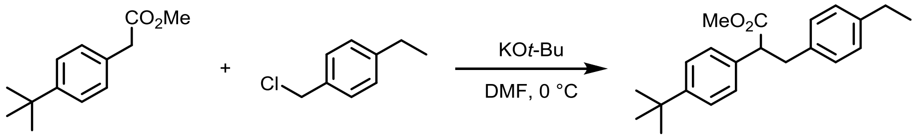

methyl 2-(4-(tert-butyl)phenyl)-3-(4-ethylphenyl)propanoate: The ester (2.6 g, 12.8 mmol, 4.0 equiv.) and 4-ethylbenzyl chloride (500 mg, 3.2 mmol, 1.0 equiv.) were dissolved in 10 mL DMF and cooled to 0 °C. Solid potassium *tert*-butoxide (393 mg, 3.5 mmol, 1.1 equiv.) was

added, and the mixture stirred for 10 minutes. The solution was quenched with saturated aqueous NH_4Cl (5 mL) and diluted with H_2O (25 mL). Mixture extracted with hexanes (25 mL), and the organics dried over $MgSO_4$ and concentrated. Purified by column chromatography (0.75% Et_2O in pentane). The purest two fractions were combined and concentrated to give a pure sample for characterization. ^1H NMR (600 MHz; $CDCl_3$) δ 7.35-7.31 (m, 2H), 7.28-7.25 (m, 2H), 7.07 (app s, 4H,), 3.83 (dd, 1H, *J* = 9.8, 5.6 Hz), 3.56 (s, 3H), 3.39 (dd, 1H, *J* = 13.8, 9.8 Hz), 2.96 (dd, 1H, *J* = 13.8, 5.6 Hz), 2.59 (q, 2H, *J* = 7.6 Hz), 1.30 (s, 9H), 1.20 (t, 3H, *J* = 7.6 Hz); ^{13}C NMR (150 MHz, $CDCl_3$) δ 174.2, 150.3, 142.3, 136.7, 136.1, 129.0, 128.0, 127.6, 125.7, 53.4, 52.0, 39.6, 34.6, 31.5, 28.6, 15.8; IR (film): 2961, 2904, 2869, 1735 cm^{-1}; HRMS (NSI) *m/z*: $[M+H]^+$ calcd for $C_{22}H_{29}O_2$ 325.2162; found 325.2161;

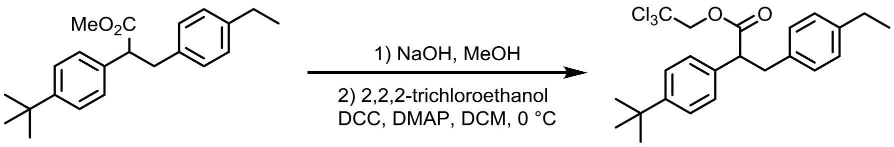

2,2,2-trichloroethyl 2-(4-(*tert*-butyl)phenyl)-3-(4-ethylphenyl)propanoate: Hydrolyzed by a procedure analogous to that for 2-(4-bromophenyl)-3-(4-ethylphenyl)propanoic acid, and esterified using the representative procedure for DCC coupling, described above. The product was purified by column chromatography (1% Et_2O in pentane) and the purest fractions combined and concentrated. ^1H NMR (600 MHz; $CDCl_3$) δ 7.37-7.33 (m, 4H), 7.13 (d, 2H, *J* = 8.1 Hz), 7.08 (d, 2H, *J* = 8.1 Hz), 4.70 (d, 1H, *J* = 12.0 Hz), 4.53 (d, 1H, *J* = 12.0 Hz), 4.00 (dd, 1H, *J* = 10.2, 5.4 Hz), 3.44 (dd, 1H, *J* = 13.9, 10.2 Hz), 3.06 (dd, 1H, *J* = 13.9, 5.4 Hz), 2.59 (q, 2H, *J* = 7.6 Hz), 1.31 (s, 9H), 1.20 (t, 3H, *J* = 7.6 Hz); ^{13}C NMR (150 MHz, $CDCl_3$) δ 172.1, 150.8, 142.7, 136.1, 135.0, 129.1, 128.1, 127.9, 125.8, 94.9, 74.3, 53.3, 39.3, 34.7, 31.5, 28.7, 15.9; IR

(film): 2962, 2904, 2869, 1751 cm^{-1}; HRMS (NSI) *m/z*: $[M+H]^+$ calcd for $C_{23}H_{28}O_2Cl_3$ 441.1149; found 441.1154;

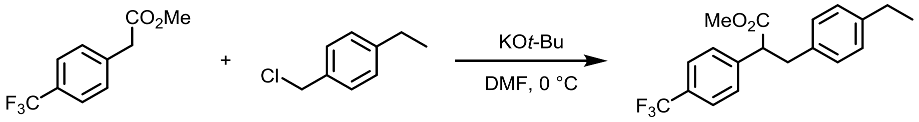

methyl 3-(4-ethylphenyl)-2-(4-(trifluoromethyl)phenyl)propanoate: The ester (2.8 g, 12.8 mmol, 4.0 equiv.) and 4-ethylbenzyl chloride (500 mg, 3.2 mmol, 1.0 equiv.) were dissolved in 10 mL DMF and cooled to 0 °C. Solid potassium *tert*-butoxide (393 mg, 3.5 mmol, 1.1 equiv.) was added, and the mixture stirred for 10 minutes. The solution was quenched with saturated aqueous NH_4Cl (5 mL) and diluted with H_2O (25 mL). Mixture extracted with hexanes (25 mL), and the organics dried over $MgSO_4$ and concentrated. Purified by column chromatography (0.75% Et_2O in pentane). The purest three fractions were combined and concentrated to give a pure sample for characterization. ^{1}H NMR (600 MHz; $CDCl_3$) δ 7.55 (d, 2H, *J* = 8.2 Hz), 7.41 (d, 2H, *J* = 8.2 Hz), 7.07 (d, 2H, *J* = 7.8 Hz), 7.01 (d, 2H, *J* = 7.8 Hz), 3.90 (dd, 1H, *J* = 8.5, 7.0 Hz), 3.61 (s, 3H), 3.40 (dd, 1H, *J* = 13.8, 8.5 Hz), 2.99 (dd, 1H, *J* = 13.8, 7.0 Hz), 2.59 (q, 2H, *J* = 7.6 Hz), 1.19 (t, 3H, *J* = 7.6 Hz); ^{13}C NMR (150 MHz, $CDCl_3$) δ 173.4, 142.8, 142.7, 135.7, 129.8 (q, *J* = 32.6 Hz), 129.0, 128.6, 128.1, 125.7 (q, *J* = 3.8 Hz), 124.3 (q, *J* = 271.9 Hz), 53.7, 52.4, 39.5, 28.6, 15.7; IR (film): 2964, 2933, 2874, 1736 cm^{-1}; HRMS (NSI) *m/z*: $[M+Na]^+$ calcd for $C_{19}H_{19}O_2F_3Na$ 359.1229; found 359.1231;

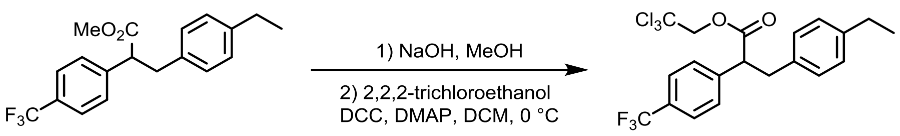

2,2,2-trichloroethyl 3-(4-ethylphenyl)-2-(4-(trifluoromethyl)phenyl)propanoate: Hydrolyzed by a procedure analogous to that for 2-(4-bromophenyl)-3-(4-ethylphenyl)propanoic acid, and esterified using the representative procedure for DCC coupling, described above. The product was purified by column chromatography (1% Et_2O in pentane) and the purest fractions combined and concentrated.

Products from functionalization at the secondary position of 4-ethyltoluene were prepared according to the following scheme. The products were generally isolated as approximately 1:1 mixtures of diastereomers. NMR peaks for ^{1}H and ^{13}C NMR are reported as the mixtures. Where major and minor diasteromer designations appear, these refer to the major and minor diastereomers resulting from the S_N2 reaction, as shown below.

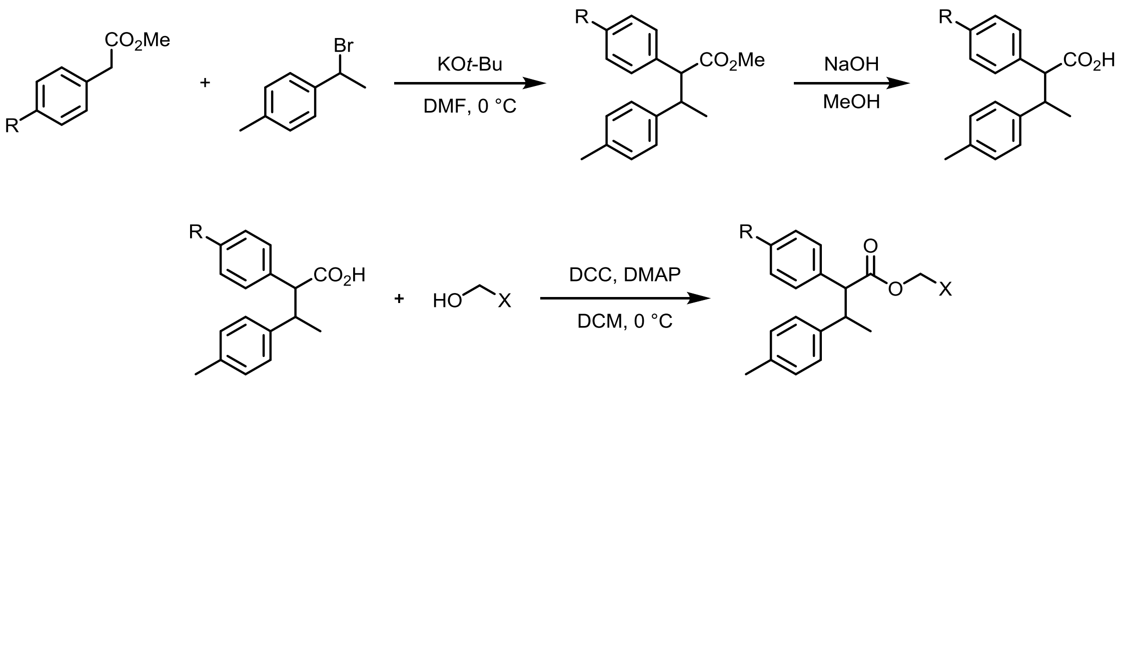

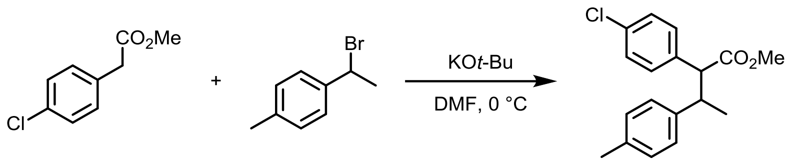

methyl 2-(4-chlorophenyl)-3-(*p*-tolyl)butanoate: The ester (111 mg, 0.6 mmol, 1.2 equiv.) and the benzyl bromide (100 mg, 0.5 mmol, 1.0 equiv.) were dissolved in 2 mL DMF, and the solution cooled to 0 °C. Solid potassium *tert*-butoxide (62 mg, 0.55 mmol, 1.1 equiv.) was added in one portion, and the solution stirred for 10 minutes. It was quenched with saturated aqueous NH_4Cl (2 mL), diluted with H_2O (10 mL) and extracted with hexanes (15 mL). The organics were dried over $MgSO_4$ and concentrated. Purified by column chromatography (1% Et_2O in pentane). ^1H NMR (600 MHz; $CDCl_3$) δ 7.39-7.36 (m, 2H), 7.33-7.30 (m, 2H), 7.18-7.14 (m, 2H), 7.13-7.09 (m, 2H), 7.09-7.05 (m, 4H), 6.94-6.90 (m, 2H), 6.84 (d, 2H, *J* = 8.1 Hz), 3.69 (s, 3H), 3.67 (m, 2H, overlap), 3.41-3.34 (m, 5H, overlap), 2.31 (s, 3H), 2.20 (s, 3H), 1.34 (d, 3H, *J* = 6.8 Hz), 0.99 (d, 3H, *J* = 6.8 Hz); ^{13}C NMR (150 MHz, $CDCl_3$) δ 174.0, 173.3, 141.5, 140.3, 136.4, 136.3, 136.3, 136.0, 133.6, 133.0, 130.1, 130.0, 129.4, 129.1, 129.0, 128.5, 127.5, 127.3, 59.1, 58.7, 52.3, 51.9, 43.6, 43.2, 21.4, 21.3, 21.2, 20.1; IR (film): 3022, 2950, 2873, 1733 cm^{-1}; HRMS (NSI) *m/z*: $[M+Na]^+$ calcd for $C_{18}H_{19}O_2ClNa$ 325.0966; found 325.0966;

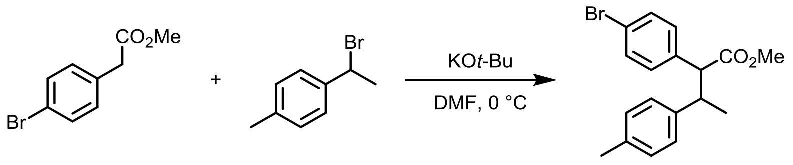

methyl 2-(4-bromophenyl)-3-(*p*-tolyl)butanoate: The ester (1.3 g, 5.5 mmol, 1.1 equiv.) and the benzyl bromide (1.0 g, 5.0 mmol, 1.0 equiv.) were dissolved in 20 mL DMF, and the solution cooled to 0 °C. Solid potassium *tert*-butoxide (617 mg, 5.5 mmol, 1.1 equiv.) was added in one portion, and the solution stirred for 10 minutes. It was quenched with saturated aqueous NH_4Cl

(5 mL), diluted with H_2O (25 mL) and extracted with hexanes (35 mL). The organics were dried over $MgSO_4$ and concentrated. Purified by column chromatography (1% Et_2O in pentane). This was used immediately in the next step:

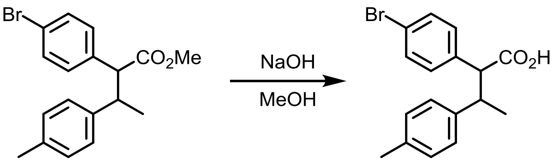

2-(4-bromophenyl)-3-(*p*-tolyl)butanoic acid: The ester (1.1 g, 3.2 mmol, 1.0 equiv.) was dissolved in 25 mL MeOH, and a 1.0 M solution of NaOH in water (6.3 mL, 6.3 mmol, 2.0 equiv.) was added. The solution was heated to reflux, and MeOH was dded in 1 mL portions until the solution became homogenous. The reaction was stirred at reflux overnight, cooled to room temperature, and diluted with 1M NaOH (10 mL) and water (50 mL). The solution was washed with Et_2O (25 mL) and then acidified to pH = 1.0. The acidic solution was extracted with Et_2O (45 mL). The organics were dried over $MgSO_4$ and concentrated. Crude product isolated as a while solid, and used without further purification.

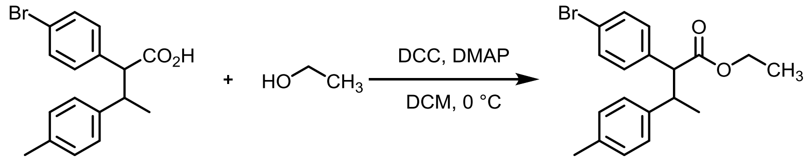

ethyl 2-(4-bromophenyl)-3-(*p*-tolyl)butanoate: Prepared by the representative DCC coupling procedure, described previously. ^{1}H NMR (600 MHz; $CDCl_3$) δ 7.50-7.44 (m, 2H, major), 7.37-7.31 (m, 2H, major), 7.26-7.21 (m, 2H, minor), 7.17 (d, 2H, *J* = 8.1 Hz, major), 7.10 (d, 2H, *J* = 7.8 Hz, major), 7.06-7.00 (m, 2H, minor), 6.92 (d, 2H, *J* = 7.8 Hz, minor), 6.85 (d, 2H, *J* = 8.1 Hz, minor), 4.24-4.16 (m, 1H, minor), 4.15-4.07 (m, 1H, minor), 3.90-3.74 (m, 2H, major), 3.67-

3.60 (m, 2H, overlap), 3.43-3.31 (m, 2H, overlap), 2.31 (s, 3H, major), 2.21 (s, 3H, minor), 1.35 (d, 3H, *J* = 6.8 Hz, minor), 1.24 (t, 3H, *J* = 7.1 Hz, minor), 1.00 (d, 3H, *J* = 7.0 Hz, major), 0.91 (t, 3H, *J* = 7.1 Hz, major); ^{13}C NMR (150 MHz, $CDCl_3$) δ 173.4, 172.7, 141.5, 140.4, 137.1, 137.0, 136.3, 135.9, 131.9, 131.4, 130.5, 130.4, 129.2, 129.1, 127.5, 127.4, 121.6, 121.1, 61.1, 60.7, 59.3, 58.9, 43.5, 43.4, 21.3, 21.2, 21.1, 20.3, 14.3, 14.0; IR (film): 2974, 2932, 2872, 1729 cm^{-1}; HRMS (NSI) *m/z*: $[M+Na]^+$ calcd for $C_{19}H_{21}O_2BrNa$ 383.0617; found 383.0620;

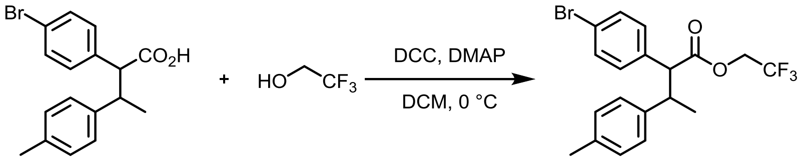

2,2,2-trifluoroethyl 2-(4-bromophenyl)-3-(*p*-tolyl)butanoate: Prepared by the representative DCC coupling procedure, described previously. ^{1}H NMR (600 MHz; $CDCl_3$) δ 7.52-7.48 (m, 2H, major), 7.35-7.31 (m, 2H, major), 7.30-7.25 (m, 2H, minor), 7.16 (d, 2H, *J* = 8.1 Hz, major), 7.12 (d, 2H, *J* = 7.8 Hz, major), 7.04-7.00 (m, 2H, minor), 6.94 (d, 2H, *J* = 7.8 Hz, minor), 6.86 (d, 2H, *J* = 8.1 Hz, minor), 4.65-4.56 (m, 1H, minor), 4.42-4.32 (m, 1H, minor), 4.18-4.05 (m, 2H, major), 3.79-3.74 (m, 2H, overlap), 3.44-3.34 (m, 2H, overlap), 2.31 (s, 3H, major), 2.22 (s, 3H, minor), 1.37 (d, 3H, *J* = 6.8 Hz, minor), 1.02 (d, 3H, *J* = 7.0 Hz, major); ^{13}C NMR (150 MHz, $CDCl_3$) δ 171.9, 171.2, 140.8, 139.7, 136.7, 136.3, 135.9, 135.8, 132.2, 131.6, 130.4, 130.4, 129.5, 129.3, 127.5, 127.2, 123.0 (q, *J* = 277.4 Hz), 122.9 (q, *J* = 277.1 Hz), 122.2, 121.6, 60.6 (overlap), 58.8, 58.4, 43.5, 43.4, 21.2, 21.2, 20.2 (overlap); IR (film): 3024, 2969, 2929, 1752 cm^{-1}; HRMS (NSI) *m/z*: $[M+Na]^+$ calcd for $C_{19}H_{18}F_3O_2BrNa$ 437.0335; found 437.0338;

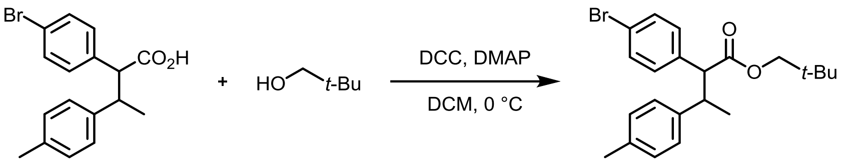

neopentyl 2-(4-bromophenyl)-3-(p-tolyl)butanoate: Prepared by the representative DCC coupling procedure, described previously. ^{1}H NMR (600 MHz; $CDCl_3$) δ 7.50-7.46 (m, 2H, major), 7.37-7.34 (m, 2H, major), 7.26-7.23 (m, 2H, minor), 7.17 (d, 2H, J = 8.1 Hz, major), 7.10 (d, 2H, J = 7.7 Hz, major), 7.06-7.04 (m, 2H, minor), 6.93 (d, 2H, J = 7.8 Hz, minor), 6.88 (d, 2H, J = 8.1 Hz, minor), 3.82 (d, 1H, J = 10.6 Hz, minor), 3.77 (d, 1H, J = 10.6 Hz, minor), 3.71-3.66 (m, 2H, overlap), 3.50 (d, 1H, J = 10.6 Hz, major), 3.43-3.35 (m, 3H, overlap), 2.30 (s, 3H, major), 2.21 (s, 3H, minor), 1.36 (d, 3H, J = 6.8 Hz, minor), 1.00 (d, 3H, J = 7.0 Hz, major), 0.90 (s, 9H, minor), 0.69 (s, 9H, major); ^{13}C NMR (150 MHz, $CDCl_3$) δ 173.4, 172.9, 131.5, 140.4, 137.2, 137.0, 136.4, 136.0, 131.9, 131.4, 130.5, 130.5, 129.4, 129.2, 127.5, 127.4, 121.6, 121.1, 74.4, 74.1, 59.5, 59.2, 43.2, 43.2, 31.6, 31.3, 26.6, 26.4, 21.6, 21.2, 21.2, 20.4; IR (film): 2959, 2869, 1731, 1152 cm^{-1}; HRMS (NSI) *m/z*: $[M+H]^+$ calcd for $C_{22}H_{28}O_2Br$ 403.1267; found 403.1274;

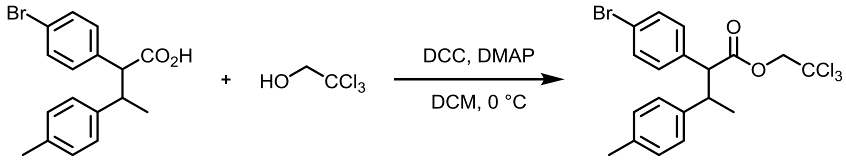

2,2,2-trichloroethyl 2-(4-bromophenyl)-3-(*p*-tolyl)butanoate: Prepared by the representative DCC coupling procedure, described previously. ^{1}H NMR (600 MHz; $CDCl_3$) δ 7.51-7.47 (m, 2H, major), 7.38-7.35 (m, 2H, major), 7.27-7.24 (m, 2H, minor), 7.19 (d, 2H, *J* = 8.0 Hz, major), 7.09 (d, 2H, *J* = 7.7 Hz, major), 7.08-7.04 (m, 2H, minor), 6.93 (d, 2H, *J* = 7.8 Hz, minor), 6.87 (d, 2H, *J* = 8.1 Hz, minor), 4.81 (d, 1H, *J* = 12.0 Hz, minor), 4.67 (d, 1H, *J* = 12.0 Hz, minor),

4.49 (d, 1H, *J* = 12.0 Hz, major), 4.32 (d, 1H, *J* = 12.0 Hz, major), 3.82-3.78 (m, 2H, overlap), 3.49-3.39 (m, 2H, overlap), 2.29 (s, 3H, major), 2.21 (s, 3H, minor), 1.40 (d, 3H, *J* = 6.8 Hz, minor), 1.02 (d, 3H, *J* = 7.0 Hz, major); ^{13}C NMR (150 MHz, $CDCl_3$) δ 171.8, 171.1, 141.0, 139.9, 136.7, 136.2, 136.0, 135.9, 132.1, 131.6, 130.6, 130.5, 129.5, 129.3, 127.5, 127.4, 122.1, 121.6, 94.9, 94.7, 74.5, 74.2, 59.0, 58.7, 43.2, 43.1, 21.5, 21.2, 21.2, 20.3; IR (film): 3023, 2964, 2873, 1749 cm^{-1}; HRMS (NSI) *m/z*: $[M+Na]^+$ calcd for $C_{19}H_{18}O_2Cl_3BrNa$ 484.9448; found 484.9455;

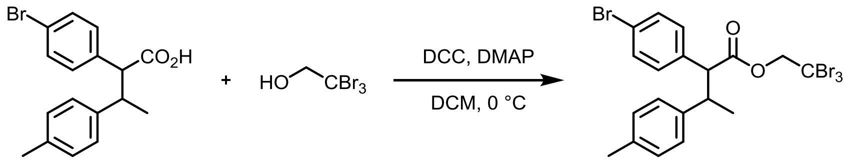

2,2,2-tribromoethyl 2-(4-bromophenyl)-3-(*p*-tolyl)butanoate: Prepared by the representative DCC coupling procedure, described previously. ^{1}H NMR (600 MHz; $CDCl_3$) δ 7.52-7.47 (m, 2H, major), 7.42-7.38 (m, 2H, major), 7.28-7.24 (m, 2H, minor), 7.24-7.21 (m, 2H, major), 7.12-7.08 (m, 4H, overlap), 6.49 (d, 2H, J = 7.9 Hz, minor), 6.89 (d, 2H, J = 7.9 Hz, minor), 4.98 (d, 1H, J = 12.2 Hz, minor), 4.88 (d, 1H, J = 12.2 Hz, minor), 4.70 (d, 1H, J = 12.2 Hz, major), 4.52 (d, 1H, J = 12.2 Hz, major), 3.86-3.81 (m, 2H, overlap), 3.53-3.43 (m, 2H, overlap), 2.29 (s, 3H, major), 2.21 (s, 3H, minor), 1.44 (d, 3H, J = 6.8 Hz, minor), 1.04 (d, 3H, J = 7.0 Hz, major); ^{13}C NMR (150 MHz, $CDCl_3$) δ 171.4, 170.6, 141.0, 139.8, 136.5, 136.0, 135.9, 135.8, 131.9, 131.4, 130.5, 130.5, 129.4, 129.1, 127.4, 127.4, 122.0, 121.4, 76.9, 76.9, 59.0, 58.7, 43.0, 42.8, 35.2, 35.1, 21.6, 21.1, 21.1, 20.2; IR (film): 3022, 2965, 2938, 2871, 1745 cm^{-1}; HRMS (NSI) *m/z*: $[M+H]^+$ calcd for $C_{19}H_{19}O_2Br_4$ 594.8113; found 594.8125;

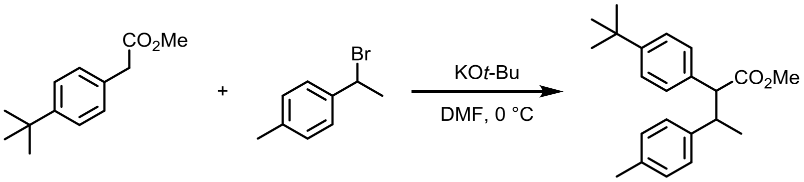

methyl 2-(4-(tert-butyl)phenyl)-3-(*p*-tolyl)butanoate: Prepared by a method analogous to methyl 2-(4-bromophenyl)-3-(*p*-tolyl)butanoate, described above. ^{1}H NMR (600 MHz; $CDCl_3$) δ 7.40-7.34 (m, 4H, major), 7.20 (d, 2H, J = 8.1 Hz, major), 7.16-7.13 (m, 2H, minor), 7.11 (d, 2H, J = 7.8 Hz, major), 7.09-7.06 (m, 2H, minor), 6.92 (d, 2H, J = 8.0 Hz, minor), 6.88 (d, 2H, J = 8.2 Hz, minor), 3.71-3.66 (m, 5H, overlap), 3.45-3.38 (m, 2H, overlap), 3.37 (s, 3H, major), 2.32 (s, 3H, major), 2.21 (s, 3H, minor), 1.35 (d, 3H, J = 6.8 Hz, minor), 1.33 (s, 9H, major), 1.22 (s, 9H, minor), 1.00 (d, 3H, J = 7.0 Hz, major); ^{13}C NMR (150 MHz, $CDCl_3$) δ 174.5, 173.8, 150.5, 149.8, 142.1, 140.9, 136.1, 135.6, 134.8, 134.6, 129.3, 128.9, 128.3, 128.3, 127.6, 127.3, 125.7, 125.2, 59.3, 58.8, 52.1, 51.8, 43.4, 43.2, 34.7, 34.5, 31.6, 31.5, 21.3, 21.2, 21.2, 20.5; IR (film): 2961, 2869, 1735, 1155 cm^{-1}; HRMS (NSI) *m/z*: $[M+Na]^+$ calcd for $C_{22}H_{28}O_2Na$ 347.1982; found 347.1977;

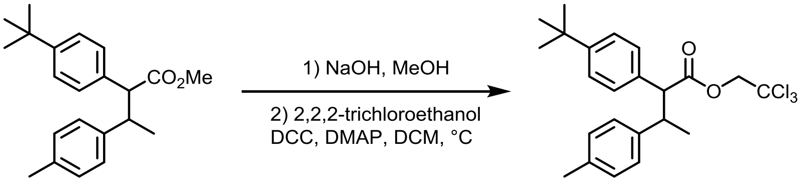

2,2,2-trichloroethyl 2-(4-(*tert*-butyl)phenyl)-3-(*p*-tolyl)butanoate: Hydrolyzed by a procedure analogous to that for 2-(4-bromophenyl)-3-(*p*-tolyl)butanoic acid, and esterified using the representative procedure for DCC coupling, described above. The product was purified by column chromatography (1% Et_2O in pentane) and the purest fractions combined and concentrated. ^{1}H NMR (600 MHz; $CDCl_3$) δ 7.45-7.41 (m, 2H, major), 7.41-7.37 (m, 2H, major), 7.26-7.22 (m, 2H, major), 7.18-7.09 (m, 6H, overlap), 6.96-6.90 (m, 4H, minor), 4.68 (d, 1H, *J* =

12.0 Hz, minor), 4.63 (d, 1H, J = 12.0 Hz, minor), 4.54 (d, 1H, J = 11.9 Hz, major), 4.28 (d, 1H, J = 11.0 Hz, major), 3.87-3.81 (m, 2H, overlap), 3.55-3.44 (m, 2H, overlap), 2.31 (s, 3H, major), 2.22 (s, 3H, minor), 1.41 (d, 3H, J = 6.8 Hz, minor), 1.32 (s, 9H, major), 1.21 (s, 9H, minor), 1.03 (d, 3H, J = 7.0 Hz, major); ^{13}C NMR (150 MHz, $CDCl_3$) δ 172.4, 171.7, 150.9, 150.2, 141.6, 140.5, 136.4, 135.8, 134.0, 133.7, 129.4, 129.0, 128.5, 128.4, 127.6, 127.5, 125.8, 125.3, 95.1, 94.9, 74.4, 74.1, 59.1, 58.8, 43.2, 43.0, 34.7, 34.5, 31.6, 31.5, 21.3, 21.2, 21.2, 20.6; IR (film): 2962, 2869, 1750, 1131 cm^{-1}; HRMS (NSI) *m/z*: $[M+H]^+$ calcd for $C_{23}H_{28}O_2Cl_3$ 441.1149; found 441.1152;

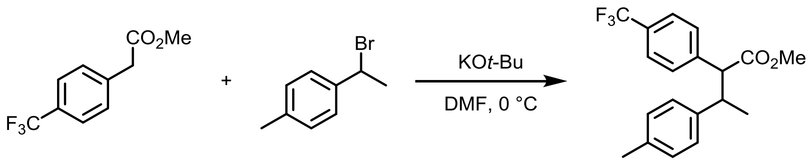

methyl 3-(*p*-tolyl)-2-(4-(trifluoromethyl)phenyl)butanoate: Prepared by a method analogous to methyl 2-(4-bromophenyl)-3-(*p*-tolyl)butanoate, described above. ^{1}H NMR (600 MHz; $CDCl_3$) δ 7.62 (d, 2H, J = 8.2 Hz, major), 7.58 (d, 2H, J = 8.2 Hz, major), 7.38 (d, 2H, J = 8.2 Hz, minor), 7.27 (d, 2H, J = 8.2 Hz, minor), 7.18 (d, 2H, J = 7.9 Hz, major), 7.12 (d, 2H, J = 7.9 Hz, major), 6.92 (d, 2H, J = 7.9 Hz, minor), 6.85 (d, 2H, J = 7.9 Hz, minor), 3.80-3.74 (m, 2H, overlap), 3.71 (s, 3H, minor), 3.47-3.40 (m, 2H, overlap), 3.39 (s, 3H, major), 2.32 (s, 3H, major), 2.20 (s, 3H, minor), 1.37 (d, 3H, J = 6.8 Hz, minor), 1.00 (d, 3H, J = 7.0 Hz, major); ^{13}C NMR (150 MHz, $CDCl_3$) δ 173.7, 173.0, 141.9, 141.8, 141.3, 140.0, 136.5, 136.1, 130.0 (q, J = 32.8 Hz), 129.4 (overlap), 129.2 (overlap), 129.1 (overlap), 127.5, 127.3, 125.8 (q, J = 3.8 Hz), 125.3 (q, J = 3.2 Hz), 59.6, 59.2, 52.4, 52.1, 43.7, 43.3, 21.4, 21.3, 21.1, 21.1 (the large C-F coupling was not observed); IR (film): 2954, 1735, 1322, 1067 cm^{-1}; HRMS (NSI) *m/z*: $[M+H]^+$ calcd for $C_{19}H_{20}F_3O_2$ 337.1410; found 337.1410;

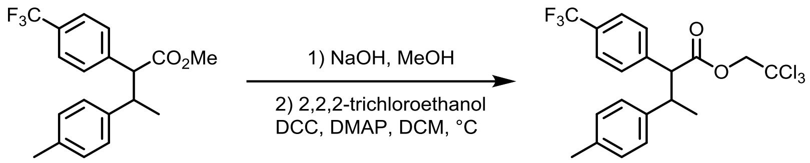

2,2,2-trichloroethyl 3-(*p*-tolyl)-2-(4-(trifluoromethyl)phenyl)butanoate: Hydrolyzed by a procedure analogous to that for 2-(4-bromophenyl)-3-(*p*-tolyl)butanoic acid, and esterified using the representative procedure for DCC coupling, described above. The product was purified by column chromatography (1% Et_2O in pentane) and the purest fractions combined and concentrated. ^1H NMR (600 MHz; $CDCl_3$) δ 7.67-7.62 (m, 4H, major), 7.41 (d, 2H, *J* = 8.1 Hz, minor), 7.33 (d, 2H, *J* = 8.1 Hz, minor), 7.23 (d, 2H, *J* = 7.8 Hz, major), 7.13 (d, 2H, *J* = 7.8 Hz, major), 6.94 (d, 2H, *J* = 7.9 Hz, minor), 6.90 (d, 2H, *J* = 7.9 Hz, minor), 4.85 (d, 1H, *J* = 12.0 Hz, minor), 4.69 (d, 1H, *J* = 12.0 Hz, minor), 4.52 (d, 1H, *J* = 12.0 Hz, major), 4.35 (d, 1H, *J* = 12.0 Hz, major), 3.96-3.91 (m, 2H, overlap), 3.57-3.47 (m, 2H, overlap), 2.31 (s, 3H, major), 2.21 (s, 3H, minor), 1.45 (d, 3H, *J* = 6.8 Hz, minor), 1.05 (d, 3H, *J* = 7.0 Hz, major); ^{13}C NMR (150 MHz, $CDCl_3$) δ 171.5, 170.8, 141.0, 140.9, 140.8, 139.6, 136.8, 136.3, 130.4 (q, *J* = 32.7 Hz), 129.5 (overlap), 129.3, 129.3, 129.3, 127.5 (overlap), 125.9 (q, *J* = 3.7 Hz), 125.4 (q, *J* = 3.7 Hz), 124.3 (q, *J* = 272.0 Hz), 124.2 (q, *J* = 271.8 Hz), 94.9, 94.7, 74.5, 74.3, 59.5, 59.2, 43.4, 43.2, 21.5, 21.2, 21.1, 20.3; IR (film): 3023, 2968, 2875, 1750 cm^{-1}; HRMS (NSI) *m/z*: $[M+H]^+$ calcd for $C_{20}H_{19}F_3O_2Cl_3$ 453.0397; found 453.0402;

4.2 Characterization of Products from Functionalization of 4-isopropyltoluene

The products arising from functionalization of 4-isopropyltoluene were slightly separable by TLC. Analytical samples of the tertiary functionalization products were obtained by direct separation of the mixtures obtained from the C–H functionalization reaction with Rh_2(*S*-DOSP)$_4$

using column chromatography or preparatory TLC (in all cases, the least polar spot in pentane/ether was the tertiary product). The primary functionalization products were isolated directly from the reactions with Rh_2(*R*-BPCP)$_4$, which gave >20:1 ratio in favor of this product for all diazo compounds investigated.

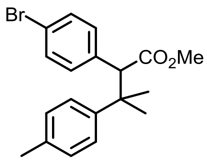

methyl 2-(4-bromophenyl)-3-methyl-3-(*p*-tolyl)butanoate: Characterization data for this product has already been reported. Spectral data matched those previously reported.[6]

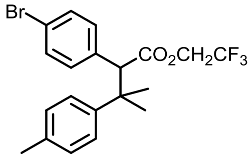

2,2,2-trifluoroethyl 2-(4-bromophenyl)-3-methyl-3-(*p*-tolyl)butanoate: ^{1}H NMR (600 MHz; $CDCl_3$) δ 7.36 (d, 2H, *J* = 8.2 Hz), 7.15 (d, 2H, *J* = 8.2 Hz), 7.08 (d, 2H, *J* = 8.4 Hz), 7.05 (d, 2H, *J* = 8.4 Hz), 4.33-4.25 (m, 1H), 4.22-4.14 (m, 1H), 3.93 (s, 1H), 2.31 (s, 3H), 1.46 (s, 3H), 1.32 (s, 3H); ^{13}C NMR (150 MHz, $CDCl_3$) δ 170.8, 143.2, 136.3, 133.7, 131.8, 131.1, 128.9, 126.4, 123.0 (q, *J* = 277.3 Hz), 122.0, 61.6, 60.3 (q, *J* = 36.6 Hz), 41.3, 26.3, 25.1, 21.1; IR (film): 3027, 2972, 2878, 1752 cm^{-1}; HRMS (NSI) *m/z*: [M+Na]$^+$ calcd for $C_{20}H_{20}F_3O_2BrNa$ 451.0491; found 451.0497;

(6) Qin, C.; Davies, H. M. L. *J. Am. Chem. Soc.* **2014**, *136*, 9792–9796.

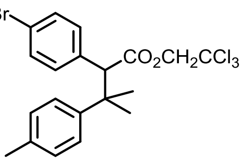

2,2,2-trichloroethyl 2-(4-bromophenyl)-3-methyl-3-(*p*-tolyl)butanoate: ^{1}H NMR (600 MHz; $CDCl_3$) δ 7.27 (d, 2H, *J* = 8.4 Hz), 7.18 (d, 2H, *J* = 8.2 Hz), 7.11-7.06 (m, 4H), 4.59 (d, 1H, *J* = 12.0 Hz), 4.45 (d, 1H, *J* = 12.0 Hz), 3.98 (s, 1H), 2.30 (s, 3H), 1.51 (s, 3H), 1.33 (s, 3H); 13C NMR (150 MHz, $CDCl_3$) δ 170.7, 143.4, 136.2, 133.9, 132.0, 131.1, 129.0, 126.5, 122.0, 94.8, 74.3, 61.9, 41.3, 26.7, 25.1, 21.1; IR (film): 3026, 2971, 2876, 1750 cm^{-1}; HRMS (NSI) *m/z*: $[M+H]^+$ calcd for $C_{20}H_{21}O_2Cl_3Br$ 476.9785; found 476.9793;

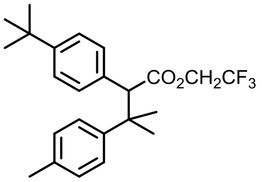

2,2,2-trifluoroethyl 2-(4-(*tert*-butyl)phenyl)-3-methyl-3-(*p*-tolyl)butanoate: ^{1}H NMR (600 MHz; $CDCl_3$) δ 7.28 (d, 2H, *J* = 8.4 Hz), 7.21 (d, 2H, *J* = 8.2 Hz), 7.18 (d, 2H, *J* = 8.4 Hz), 7.09 (d, 2H, *J* = 8.2 Hz), 4.22 (dq, 1H, *J* = 12.7, 8.6 Hz), 4.06 (dq, 1H, *J* = 12.7, 8.5 Hz), 3.96 (s, 1H), 2.32 (s, 3H), 1.48 (s, 3H), 1.30 (s, 9H), 1.29 (s, 3H); ^{13}C NMR (150 MHz, $CDCl_3$) δ 171.2, 150.6, 143.9, 136.0, 131.6, 129.9, 128.8, 126.4, 124.9, 61.9, 60.2 (q, *J* = 36.3 Hz), 41.3, 34.7, 31.5, 27.6, 24.1, 21.1 (The CF_3 carbon quartet could not be definitively distinguished); IR (film): 2965, 2871, 1754, 1166 cm^{-1}; HRMS (NSI) *m/z*: $[M+Na]^+$ calcd for $C_{24}H_{29}F_3O_2Na$ 429.2012; found 429.2012;

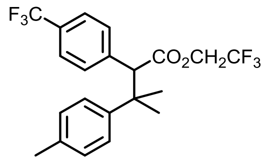

2,2,2-trifluoroethyl 3-methyl-3-(*p*-tolyl)-2-(4-(trifluoromethyl)phenyl)butanoate: ^{1}H NMR (600 MHz; $CDCl_3$) δ 7.50 (d, 2H, *J* = 8.2 Hz), 7.31 (d, 2H, *J* = 8.2 Hz), 7.15 (d, 2H, *J* = 8.2 Hz), 7.09 (d, 2H, *J* = 8.2 Hz), 4.30 (dq, 1H, *J* = 12.6, 8.5 Hz), 4.19 (dq, 1H, *J* = 12.6, 8.4 Hz), 4.03 (s, 1H), 2.32 (s, 3H), 1.49 (s, 3H), 1.34 (s, 3H); ^{13}C NMR (150 MHz, $CDCl_3$) δ 170.6, 143.0, 138.7, 136.4, 130.5, 130.0 (q, *J* = 32.5 Hz), 129.0, 126.4, 124.9 (q, *J* = 3.8 Hz), 124.3 (q, *J* = 272.1 Hz), 122.9 (q, *J* = 277.1 Hz), 62.0, 60.4 (q, *J* = 36.6 Hz), 41.6, 26.3, 25.2, 21.1; IR (film): 2974, 2885, 1755, 1326 cm^{-1}; HRMS (NSI) m/z: $[M+H]^+$ calcd for $C_{21}H_{21}F_6O_2$ 419.1440; found 419.1447;

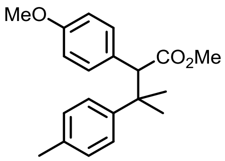

methyl 2-(4-methoxyphenyl)-3-methyl-3-(*p*-tolyl)butanoate: ^{1}H NMR (600 MHz; $CDCl_3$) δ 7.17 (d, 2H, *J* = 8.2 Hz), 7.13 (d, 2H, *J* = 8.8 Hz), 7.07 (d, 2H, *J* = 8.2 Hz), 6.77 (d, 2H, *J* = 8.8 Hz), 3.82 (s, 1H), 3.78 (s, 3H), 3.45 (s, 3H), 2.31 (s, 3H), 1.47 (s, 3H), 1.30 (s, 3H); ^{13}C NMR (150 MHz, $CDCl_3$) δ 173.4, 158.9, 144.5, 135.7, 131.3, 128.7, 127.8, 126.5, 113.2, 61.7, 55.4, 51.5, 41.1, 26.7, 25.0, 21.1; IR (film): 2950, 2836, 1734, 1511 cm^{-1}; HRMS (NSI) *m/z*: $[M+H]^+$ calcd for $C_{20}H_{25}O_3$ 313.1798; found 313.1798;

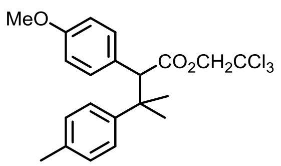

2,2,2-trichloroethyl 2-(4-methoxyphenyl)-3-methyl-3-(*p*-tolyl)butanoate: ^{1}H NMR (600 MHz; $CDCl_3$) δ 7.22 (d, 2H, *J* = 8.3 Hz), 7.18 (d, 2H, *J* = 8.7 Hz), 7.08 (d, 2H, *J* = 8.3 Hz), 6.80 (d, 2H, *J* = 8.7 Hz), 4.59 (d, 1H, *J* = 12.0 Hz), 4.39 (d, 1H, *J* = 12.0 Hz), 3.98 (s, 1H), 3.79 (s, 3H), 2.30 (s, 3H), 1.51 (s, 3H), 1.31 (s, 3H); ^{13}C NMR (150 MHz, $CDCl_3$) δ 171.3, 159.2, 144.0, 135.9, 131.4, 128.9, 127.0, 126.6, 113.4, 94.9, 74.2, 61.7, 55.4, 41.2, 27.3, 24.6, 21.1; IR (film): 2954, 2934, 2836, 1750, 1511 cm^{-1}; HRMS (NSI) *m/z*: $[M+H]^+$ calcd for $C_{21}H_{24}O_3Cl_3$ 429.0786; found 429.0790;

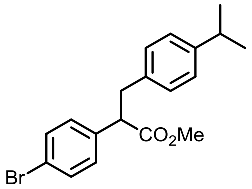

methyl 2-(4-bromophenyl)-3-(4-isopropylphenyl)propanoate: The spectral data were identical to those previously reported.[7]

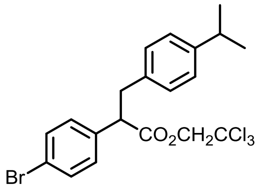

2,2,2-trichloroethyl 2-(4-bromophenyl)-3-(4-isopropylphenyl)propanoate: 1H NMR (600 MHz; $CDCl_3$) δ 7.46 (d, 2H, *J* = 8.2 Hz), 7.26 (d, 2H, *J* = 8.2 Hz), 7.11 (d, 2H, *J* = 8.1 Hz), 7.08 (d, 2H, *J* = 8.1 Hz), 4.70 (d, 1H, *J* = 12 Hz), 4.69 (d, 1H, *J* = 12 Hz), 3.97 (dd, 1H, *J* = 9.1, 6.6

(7) Qin, C.; Davies, H. M. L. *J. Am. Chem. Soc.* **2014**, *136*, 9792–9796.

Hz), 3.41 (dd, 1H, *J* = 13.9, 9.1 Hz), 3.05 (dd, 1H, *J* = 13.9, 6.6 Hz), 2.86 (sep, 1H, *J* = 6.9 Hz), 1.22 (d, 6H, *J* = 6.9 Hz); 13C NMR (100 MHz, $CDCl_3$) δ 171.6, 147.5, 136.9, 135.5, 132, 130.1, 129, 126.8, 121.9, 94.8, 74.3, 53.1, 39.2, 33.9, 24.2; IR (neat): 2958, 1741, 1373, 1138 cm^{-1}; HRMS (NSI) *m/z*: $[M-H]^-$ calcd for $C_{20}H_{19}O_2BrCl_3$ 474.964, found 474.9645;

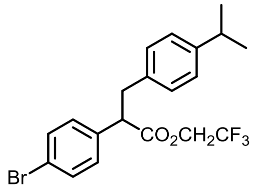

2,2,2-trifluoroethyl 2-(4-bromophenyl)-3-(4-isopropylphenyl)propanoate: ^{1}H NMR (600 MHz; $CDCl_3$) δ 7.46-7.43 (m, 2H), 7.21-7.17 (m, 2H), 7.10 (d, 2H, *J* = 8.0 Hz), 7.02 (d, 2H, *J* = 8.0 Hz), 4.44-4.31 (m, 2H), 3.91 (dd, 1H, *J* = 8.9, 6.7 Hz), 3.35 (dd, 1H, *J* = 13.9, 8.9 Hz), 3.01 (dd, 1H, *J* = 13.9, 6.7 Hz), 2.85 (hept, 1H, *J* = 6.9 Hz), 1.21 (d, 6H, *J* = 6.9 Hz); ^{13}C NMR (150 MHz, $CDCl_3$) δ 171.7, 147.6, 136.8, 135.3, 132.1, 129.9, 128.9, 126.8, 122.0, 60.8 (q, *J* = 36.7 Hz), 52.9, 39.3, 33.9, 24.2 (the CF_3 quartet could not be definitively distinguished); IR (film): 2961, 2929, 2871, 1754 cm^{-1}; HRMS (NSI) *m/z*: $[M+Na]^+$ calcd for $C_{20}H_{20}F_3O_2BrNa$ 451.0491; found 451.0496;

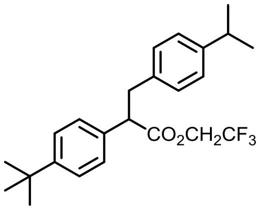

2,2,2-trifluoroethyl 2-(4-(tert-butyl)phenyl)-3-(4-isopropylphenyl)propanoate: ^{1}H NMR (600 MHz; $CDCl_3$) δ 7.38-7.35 (m, 2H), 7.32-7.28 (m, 2H), 7.13 (d, 2H, *J* = 8.1 Hz), 7.10 (d, 2H, *J* = 8.1 Hz), 4.40 (dq, 1H, *J* = 12.6, 8.5 Hz), 4.28 (dq, 1H, *J* = 12.6, 8.4 Hz), 3.96 (dd, 1H, *J* = 10.2, 5.4 Hz), 3.40 (dd, 1H, *J* = 13.9, 10.2 Hz), 3.04 (dd, 1H, *J* = 13.9, 5.4 Hz), 2.87 (hept, 1H,

J = 6.9 Hz), 1.32 (s, 9H), 1.23 (d, 6H, J = 6.9 Hz); [1]3C NMR (150 MHz, $CDCl_3$) δ 172.3, 150.9, 147.4, 136.1, 135.0, 128.9, 127.7, 126.7, 125.9, 123.0 (q, J = 277.5 Hz), 60.6 (q, J = 36.6 Hz), 53.1, 39.5, 34.7, 33.9, 31.5, 24.2; IR (film): 2961, 2871, 1755, 1135 cm^{-1}; HRMS (NSI) *m/z*: $[M+H]^+$ calcd for $C_{24}H_{30}F_3O_2$ 407.2192; found 407.2199;

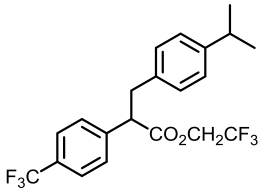

2,2,2-trifluoroethyl 3-(4-isopropylphenyl)-2-(4-(trifluoromethyl)phenyl)propanoate: ^{1}H NMR (600 MHz; $CDCl_3$) δ 7.58 (d, 2H, J = 8.1 Hz), 7.44 (d, 2H, J = 8.1 Hz), 7.11 (d, 2H, J = 8.1 Hz), 7.03 (d, 2H, J = 8.1 Hz), 4.45-4.31 (m, 2H), 4.02 (dd, 1H, J = 9.0, 6.7 Hz), 3.40 (dd, 1H, J = 13.9, 9.0 Hz), 3.05 (dd, 1H, J = 13.9, 6.7 Hz), 2.86 (hept, 1H, J = 6.9 Hz), 1.21 (d, 6H, J = 6.9 Hz); ^{13}C NMR (150 MHz, $CDCl_3$) δ 171.5, 147.7, 141.7, 135.1, 130.2 (q, J = 32.5 Hz), 128.9, 128.6, 126.8, 125.9 (q, J = 3.8 Hz), 124.2 (q, J = 272.1 Hz), 122.9 (q, J = 277.1 Hz), 60.8 (q, J = 36.7 Hz), 53.3, 39.4, 33.9, 24.2; IR (film): 2964, 2931, 2874, 1756 cm^{-1}; HRMS (NSI) *m/z*: $[M+H]^+$ calcd for $C_{21}H_{21}F_6O_2$ 419.1440; found 419.1446;

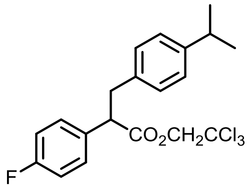

2,2,2-trichloroethyl 2-(4-fluorophenyl)-3-(4-isopropylphenyl)propanoate: ^{1}H NMR (600 MHz; $CDCl_3$) δ 7.35-7.31 (m, 2H), 7.11-7.04 (m, 4H), 7.02-6.98 (m, 2H), 4.67 (d, 1H, J = 12.0 Hz), 4.59 (d, 1H, J = 12.0 Hz), 3.97 (dd, 1H, J = 9.2, 6.5 Hz), 3.40 (dd, 1H, J = 13.9, 9.2 Hz),

3.04 (dd, 1H, *J* = 13.9, 6.5 Hz), 2.83 (hept, 1H, *J* = 6.9 Hz), 1.20 (d, 6H, *J* = 6.9 Hz); ^{13}C NMR (150 MHz, $CDCl_3$) δ 171.9, 162.5 (d, *J* = 246.1 Hz), 147.5, 135.7, 133.7 (d, *J* = 3.1 Hz), 129.9 (d, *J* = 8.0 Hz), 129.1, 126.7, 115.8 (d, *J* = 21.4 Hz), 94.9, 74.3, 52.9, 39.4, 33.9, 24.2; IR (film): 2959, 2914, 2871, 1747 cm^{-1}; HRMS (NSI) *m/z*: $[M+Na]^+$ calcd for $C_{20}H_{20}FO_2Cl_3Na$ 439.0405; found 439.0407;

5 – Supporting Information for Computational and Modeling Studies

DFT Calculations

Using Gaussian 09 software, diazo esters were energy-minimized and IR vibrations were computed according to the M06-2X functional and TZVP basis set, a combination that has been benchmarked for IR calculations.[8,9,10,11,12]

Model Development

Models were developed using the MATLAB® R2013a Stepwise Linear Regression algorithm of the Statistics Toolbox.[13] Four algorithms were typically used to inform the combinations of variables that represent descriptive, predictive models. Each algorithm begins the modeling process by starting from a prescribed set of starting terms. Linear (x1, x2, x3, etc.), interaction (x1:x2, x1:x3, etc.) and squared (x1^2, x2^2, x3^2, etc.) terms are added or removed from each model according to a p-value test. For a term to enter a model, its p-value is <0.05. For a term to exit a model, its p-value is >0.1. The four algorithms are as follows, with the nature of the starting model described.

LinearModel.stepwise(X, y)
Performs stepwise linear regression from a starting model of no variable terms.
LinearModel.stepwise(X, y, 'linear')
Performs stepwise linear regression from a starting model of linear terms.
LinearModel.stepwise(X, y, 'interactions')
Performs stepwise linear regression from a starting model of linear and interaction terms.
LinearModel.stepwise(X, y, 'purequadratic')
Performs stepwise linear regression from a starting model of linear terms and squared terms.

---

(8) Frisch, M. J.; Trucks, G. W.; Schlegel, H. B.; Scuseria, G. E.; Robb, M. A.; Cheeseman, J. R.; Scalmani, G.; Barone, V.; Mennucci, B.; Petersson, G. A., et al. *Gaussian 09, Revision C.01,* Gaussian, Inc.: Wallingford, CT, 2009.
(9)Zhao, Y.; Truhlar, D. G. *Theor. Chem. Acc.* **2007,** *120*, 215-241.
(10)Valero, R.; Gomes, J. R. B.; Truhlar, D. G.; Illas, F. *J. Chem. Phys.* **2008,** *129*, 124710.
(11)Schäfer, A.; Horn, H.; Ahlrichs, R. *J. Chem. Phys.* **1992,** *97,* 2571.
(12) Schäfer, A.; Huber, C.; Ahlrichs, R. *J. Chem. Phys.* **1994,** *100*, 5829.
(13)MATLAB, *Version 8.1.0.604 (R2013a)*. The MathWorks, Inc.: Natick, Massachusetts, 2013.

All R^2 values reported in graphs represent the adjusted R^2. R^2 values reported in MATLAB scripts are not adjusted. The models developed (using a training set of data) for the catalytic rhodium systems were evaluated for their robustness of predictive power using an external validation data set.[14] Normalization was performed on these combined data sets prior to model development.

Development of Models in Figure 4a (Variation of R')

ν_{diazo} Model with R' Variation

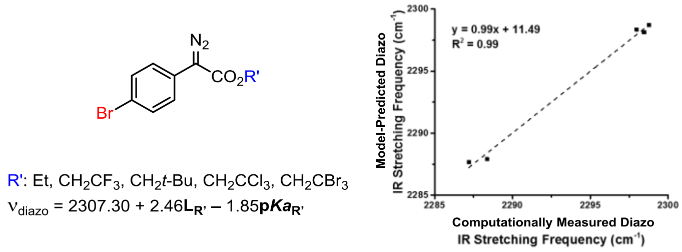

Table S1. Training Set for ν_{diazo} Model with R' Variation

Ester R'	*Diazo IR Frequency (y)*	*R' B_1 (x1)*	*R' B_5 (x2)*	*R' L (x3)*	*pK_a* (x4)*	*Molecular Weight, R' (x5)*
Et	2288.37	1.55	3.15	4.48	16.47	29.062
CH_2CF_3	2297.98	1.50	3.71	4.99	11.49	83.032
CH_2tBu	2287.20	1.54	4.42	5.42	17.85	71.143
CH_2CCl_3	2298.77	1.50	4.52	5.90	12.51	132.388
CH_2CBr_3	2298.45	1.50	4.80	6.22	13.25	265.75

*pK_a values calculated using chemicalize.org and ChemAxon.[15]

MATLAB script used for model development:

```
%
clear all;
close all;
clc;
data=[2288.37   1.55    3.15    4.48    16.47   29.062
2297.98 1.5 3.71    4.99    11.49   83.032
2287.2  1.54    4.42    5.42    17.85   71.143
2298.77 1.5 4.52    5.9 12.51   132.388
2298.45 1.5 4.8 6.22    13.25   265.75];
stdr = nanstd(data);
```

(14) Arlot, S.; Celisse, A. *Statist. Surv.* **2010,** *4*, 40-79.
(15) ChemAxon Chemicalize.Org. http://www.chemicalize.org/.

```
means = nanmean(data);
sr1 = data-repmat(means,size(data,1),1);
sr = sr1./repmat(stdr,size(data,1),1);
%sr = data./repmat(stdr,size(data,1),1);
Xz=sr(1:5,2:6);
Yz=sr(1:5,1);
X=data(1:5,2:6);
Y=data(1:5,1);

%% Afforded y ~ 1 + x1, R^2=0.95
FitForward = LinearModel.stepwise(Xz, Yz)

%% Afforded y ~ 1 + x2 + x3 + x4, R^2=1.00
FitBackL = LinearModel.stepwise(Xz, Yz, 'linear')

%% Afforded y ~ 1 + x4 + x5, R^2=0.99
FitBackI = LinearModel.stepwise(Xz, Yz, 'interactions')

%% Afforded y ~ 1 + x4 + x5, R^2=0.99
FitQuad = LinearModel.stepwise(Xz, Yz, 'purequadratic')

%% As inclusion of pKa (x4), which was identified as a necessary descriptor in three of the above four results, is intuitively congruent with describing IR frequency, various combinations of x4 and other parameters were evaluated as given below.
%% R^2=0.98
ManFit1 = LinearModel.fit (Xz, Yz, 'y ~ 1 + x1 + x4')
%% R^2=0.99
ManFit1 = LinearModel.fit (Xz, Yz, 'y ~ 1 + x2 + x4')
%% R^2=1.00
ManFit1 = LinearModel.fit (Xz, Yz, 'y ~ 1 + x3 + x4')
%% R^2=0.99
ManFit1 = LinearModel.fit (Xz, Yz, 'y ~ 1 + x4 + x5')
```

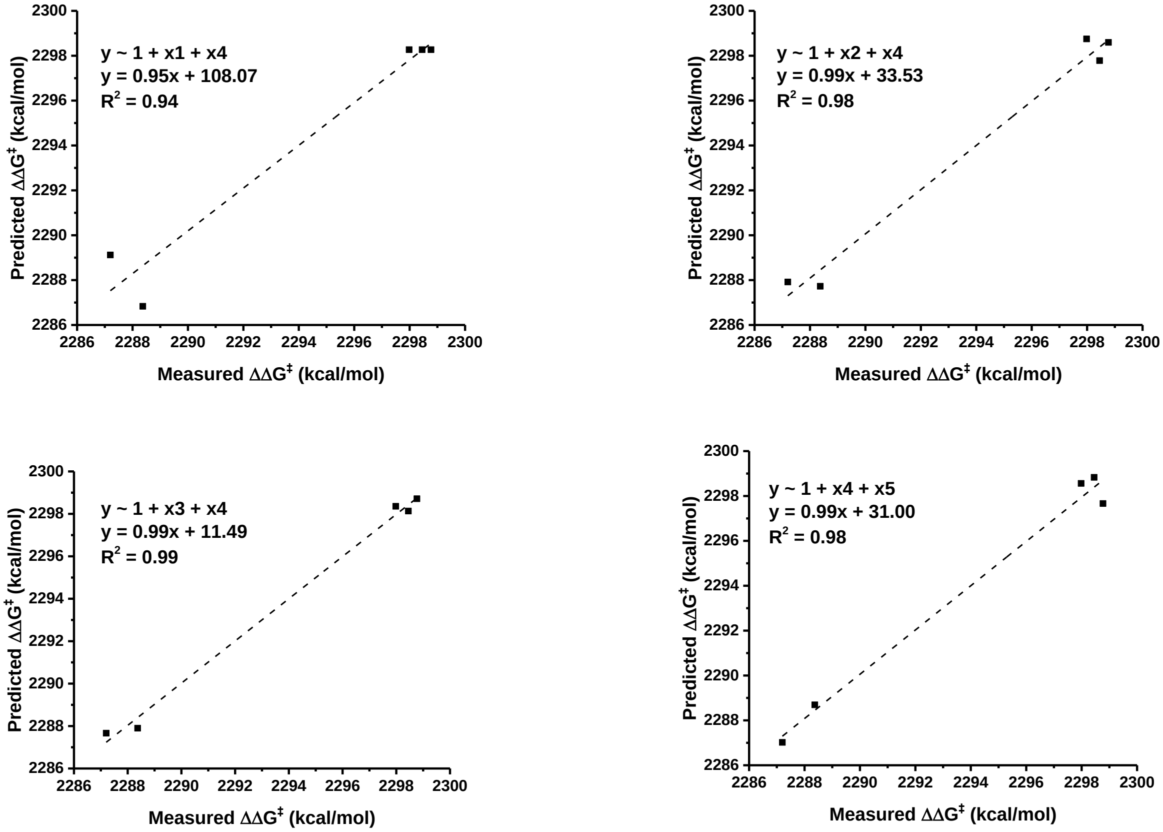

Figure S1. Graphical representation (predicted versus measured $\Delta\Delta G^{\ddagger}$ values) for the four potential models identified and noted in the above MATLAB script.

While good models are afforded for each of the linear combinations of the x4 term (pK_a) with x1, x2, x3, or x5, the best correlation was afforded with y ~ 1 + x3 + x4 (Non-normalized: $\boldsymbol{\nu}_{\mathbf{diazo}}$ = 2307.30 + 2.46$\mathbf{L}_{\mathbf{R'}}$ – 1.85$\mathbf{p}\boldsymbol{K}_{\boldsymbol{a}\mathbf{R'}}$; Normalized: $\boldsymbol{\nu}_{\mathbf{diazo}}$ = 0.29$\mathbf{L}_{\mathbf{R'}}$ – 0.86$\mathbf{p}\boldsymbol{K}_{\boldsymbol{a}\mathbf{R'}}$). In this compilation of terms, the y-intercept is minimized and the slope is maximized, two hallmarks of a model that accurately and precisely predicts the outcomes for which the model was developed (Figure 4a).

$\boldsymbol{i_{diazo}}$ *Model with R' Variation*

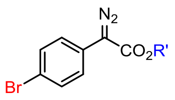

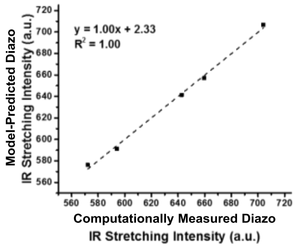

R': Et, CH_2CF_3, CH_2*t*-Bu, CH_2CCl_3, CH_2CBr_3

$i_{diazo} = 877.47 - 62.63\mathbf{L}_{\mathbf{R'}} + 6.66\mathbf{pKa}_{\mathbf{R'}}$

Table S2. Training Set for i_{diazo} Model with R' Variation

Ester R'	*Diazo IR Intensity (y)*	*R' B_1 (x1)*	*R' B_5 (x2)*	*R' L (x3)*	*pK_a** *(x4)*	*Molecular Weight, R' (x5)*
Et	704.0457	1.55	3.15	4.48	16.47	29.062
CH_2CF_3	642.6425	1.50	3.71	4.99	11.49	83.032
CH_2*t*Bu	659.6421	1.54	4.42	5.42	17.85	71.143
CH_2CCl_3	594.1926	1.50	4.52	5.90	12.51	132.388
CH_2CBr_3	572.0623	1.50	4.80	6.22	13.25	265.75

*pK_a values calculated using chemicalize.org and ChemAxon.[16]

MATLAB script used for model development:

```
%
clear all;
close all;
clc;
data=[704.0457  1.55    3.15    4.48    16.47   29.062
642.6425    1.5 3.71    4.99    11.49   83.032
659.6421    1.54    4.42    5.42    17.85   71.143
594.1926    1.5 4.52    5.9 12.51   132.388
572.0623    1.5 4.8 6.22    13.25   265.75];
stdr = nanstd(data);
means = nanmean(data);
sr1 = data-repmat(means,size(data,1),1);
sr = sr1./repmat(stdr,size(data,1),1);
%sr = data./repmat(stdr,size(data,1),1);
Xz=sr(1:5,2:6);
Yz=sr(1:5,1);
X=data(1:5,2:6);
Y=data(1:5,1);
%%
FitForward = LinearModel.stepwise(Xz, Yz)
FitBackL = LinearModel.stepwise(Xz, Yz, 'linear')
FitBackI = LinearModel.stepwise(Xz, Yz, 'interactions')
FitQuad = LinearModel.stepwise(Xz, Yz, 'purequadratic')

%% y ~ 1 + x3 + x4, R^2=1.00 was identfied for each of the above stepwise algorithms, except
FitQuad (y ~ 1 + x5 + x5^2, R^2=0.99).

%% R^2=1.00
ManFit1 = LinearModel.fit (Xz, Yz, 'y ~ 1 + x3 + x4')
```

(16) ChemAxon Chemicalize.Org. http://www.chemicalize.org/.

The model y ~ 1 + x3 + x4 (Non-normalized: $\mathbf{i_{diazo}}$ = 877.47 – 62.63$\mathbf{L_{R'}}$ + 6.66$\mathbf{p}\boldsymbol{K_{aR'}}$; Normalized: $\mathbf{i_{diazo}}$ = -0.83$\mathbf{L_{R'}}$ + 0.34$\mathbf{p}\boldsymbol{K_{aR'}}$) demonstrates accuracy and precision in its prediction of diazo IR stretching intensities (Figure 4a).

Development of Models in Figure 4b (Variation of R)

ν_{diazo} Model with R Variation

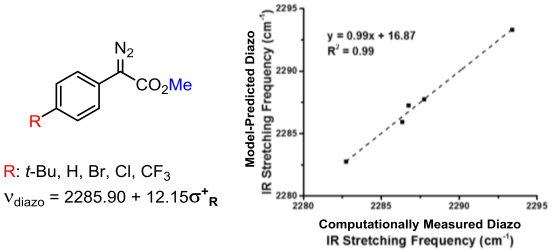

Table S3. Training Set for ν_{diazo} Model with R Variation

Aryl R	*Diazo IR Frequency (y)*	*Aryl σ (x1)*	*Aryl σ$^+$ (x2)*	*R B_5 (x3)*	*R L (x4)*	*Molecular Weight, Arene (x5)*
t-Bu	2282.73	-0.20	-0.26	3.17	4.11	133.214
H	2286.34	0.00	0.00	1.00	2.06	77.106
Br	2287.76	0.23	0.15	1.95	3.82	156.002
Cl	2286.75	0.23	0.11	1.80	3.52	111.548
CF_3	2293.40	0.54	0.61	2.61	3.30	145.103

As good correlations have been demonstrated between Hammett σ values and the carbonyl IR stretching frequency of aryl ketones, we hypothesized that σ or σ$^+$ values would effectively describe the electronic impact of arene substitution on the frequency of the diazo IR stretch.[17,18,19] Therefore, the correlations between x1 and ν_{diazo} and x2 and ν_{diazo} were assessed, as presented in the following script.

MATLAB script used for model development:

```
%
clear all;
close all;
clc;
data=[2282.73   -0.2    -0.26   3.17    4.11    133.214
2286.34 0   0   1   2.06    77.106
2287.76 0.23    0.15    1.95    3.82    156.002
2286.75 0.23    0.11    1.8 3.52    111.548
2293.4  0.54    0.61    2.61    3.3 145.103];
```

(17) Jones, R. N.; Forbes, W. F.; Mueller, W. A. *Can. J. Chem.* **1957,** *35*, 504-514.
(18) Bess, E. N.; Bischoff, A. J.; Sigman, M. S. *Proc. Natl. Acad. Sci. U.S.A* **2014**.
(19) Milo, A.; Bess, E. N.; Sigman, M. S. *Nature* **2014,** *507*, 210-214.

```
stdr = nanstd(data);
means = nanmean(data);
sr1 = data-repmat(means,size(data,1),1);
sr = sr1./repmat(stdr,size(data,1),1);
%sr = data./repmat(stdr,size(data,1),1);
Xz=sr(1:5,2:6);
Yz=sr(1:5,1);
X=data(1:5,2:6);
Y=data(1:5,1);
%%
FitForward = LinearModel.stepwise(Xz, Yz)
FitBackL = LinearModel.stepwise(Xz, Yz, 'linear')
FitBackI = LinearModel.stepwise(Xz, Yz, 'interactions')
FitQuad = LinearModel.stepwise(Xz, Yz, 'purequadratic')
%% R^2=0.92
ManFit1 = LinearModel.fit (X, Y, 'y ~ 1 + x1')
%% R^2=0.99
ManFit1 = LinearModel.fit (X, Y, 'y ~ 1 + x2')
```

While the correlation of ν_{diazo} with σ is good (non-adjusted R^2=0.92), the correlation with σ^+ (Non-normalized: $\boldsymbol{\nu_{diazo}}$ = 2285.90 + 12.15$\boldsymbol{\sigma^+_{R'}}$; Normalized: $\boldsymbol{\nu_{diazo}}$ = 1.00$\boldsymbol{\sigma^+_{R'}}$) is essentially perfect (non-adjusted R^2=0.99), as demonstrated in Figure 4b.

i_{diazo} Model with R Variation

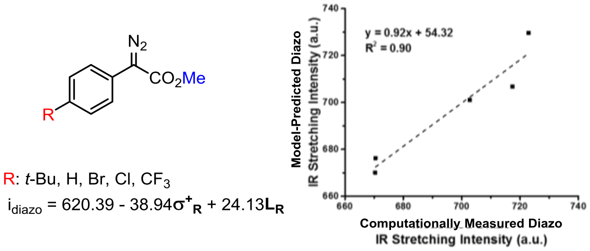

Table S4. Training Set for i_{diazo} Model with R Variation

Aryl R	*Diazo IR Frequency (y)*	*Aryl σ (x1)*	*Aryl σ⁺ (x2)*	*R B₅ (x3)*	*R L (x4)*	*Molecular Weight, Arene (x5)*
t-Bu	722.8714	-0.20	-0.26	3.17	4.11	133.214
H	670.4157	0.00	0.00	1.00	2.06	77.106
Br	717.3972	0.23	0.15	1.95	3.82	156.002
Cl	702.7315	0.23	0.11	1.80	3.52	111.548
CF_3	670.4399	0.54	0.61	2.61	3.30	145.103

Neither σ (x1) nor σ^+ (x2) could solely describe the observed trends in the intensity of the diazo IR stretch. Thus, combinations of σ (x1) or σ^+ (x2) with each of the terms x3, x4, and x5 were assessed, as given in the below MATLAB script.

MATLAB script used for model development:

```
%
clear all;
close all;
clc;
data=[722.8714  -0.2    -0.26   3.17    4.11    133.214
670.4157    0   0   1   2.06    77.106
717.3972    0.23    0.15    1.95    3.82    156.002
702.7315    0.23    0.11    1.8 3.52    111.548
670.4399    0.54    0.61    2.61    3.3 145.103];
stdr = nanstd(data);
means = nanmean(data);
sr1 = data-repmat(means,size(data,1),1);
sr = sr1./repmat(stdr,size(data,1),1);
%sr = data./repmat(stdr,size(data,1),1);
Xz=sr(1:5,2:6);
Yz=sr(1:5,1);
X=data(1:5,2:6);
Y=data(1:5,1);
%%
FitForward = LinearModel.stepwise(Xz, Yz)
FitBackL = LinearModel.stepwise(Xz, Yz, 'linear')
FitBackI = LinearModel.stepwise(Xz, Yz, 'interactions')
FitQuad = LinearModel.stepwise(Xz, Yz, 'purequadratic')
%% R^2=0.23
ManFit1 = LinearModel.fit (X, Y, 'y ~ 1 + x1')
%% R^2=0.36
ManFit1 = LinearModel.fit (X, Y, 'y ~ 1 + x2')
%% R^2=0.42
ManFit1 = LinearModel.fit (X, Y, 'y ~ 1 + x1 + x3')
%% R^2=0.56
ManFit1 = LinearModel.fit (X, Y, 'y ~ 1 + x2 + x3')
%% R^2=0.88
ManFit1 = LinearModel.fit (X, Y, 'y ~ 1 + x1 + x4')
%% ***R^2=0.92***
ManFit1 = LinearModel.fit (X, Y, 'y ~ 1 + x2 + x4')
%% R^2=0.75
ManFit1 = LinearModel.fit (X, Y, 'y ~ 1 + x1 + x5')
%% R^2=0.89
ManFit1 = LinearModel.fit (X, Y, 'y ~ 1 + x2 + x5')
```

The model y ~ 1 + x2 + x4 (Non-normalized: $\mathbf{i}_{\mathbf{diazo}} = 620.39 - 38.94\boldsymbol{\sigma}^{+}_{\mathbf{R'}} + 24.13\mathbf{L}_{\mathbf{R'}}$; Normalized: $\mathbf{i}_{\mathbf{diazo}} = -0.49\boldsymbol{\sigma}^{+}_{\mathbf{R'}} + 0.76\mathbf{L}_{\mathbf{R'}}$) demonstrates accuracy and precision in its prediction of diazo IR stretching intensities (Figure 4b).

Development of Model for Rh_2(*S*-DOSP)$_4$ (Figure 5a)

The training and external validation sets that were used for model development and validation are given in Table S5.

Table S5. Training and External Validation Sets for Rh_2(*S*-DOSP)$_4$ Model Development.

Ester, R	*Aryl, R'*	*Toluene Substrate*	$\Delta\Delta G^{\ddagger}$ (*y*)	ν_{diazo} (*x1*)	i_{diazo} (*x2*)	*q* (*x3*)
**Training Set**						
Me	*t*Bu	4-ethyl	1.92	2282.73	722.8714	-0.415
Me	CF_3	4-ethyl	1.48	2293.4	670.4399	-0.415
Me	Br	4-*iso*propyl	0.41	2287.76	717.3972	-0.232
CH_2CF_3	Br	4-ethyl	1.54	2297.98	642.6425	-0.415
CH_2CF_3	*t*Bu	4-*iso*propyl	0.97	2294.67	647.7295	-0.232
CH_2CF_3	Br	4-*iso*propyl	0.38	2297.98	642.6425	-0.232
CH_2CF_3	CF_3	4-*iso*propyl	0.22	2304.36	599.2573	-0.232
CH_2CCl_3	*t*Bu	4-ethyl	1.41	2295.14	598.3918	-0.415
CH_2CCl_3	CF_3	4-ethyl	1.01	2304.59	552.1165	-0.415
CH_2CCl_3	Br	4-*iso*propyl	-0.48	2298.77	594.1926	-0.232
**External Validation Set**						
Me	Cl	4-ethyl	1.65	2286.75	702.7315	-0.415
CH_2CH_3	Br	4-ethyl	1.61	2288.37	704.0457	-0.415
CH_2CBr_3	Br	4-ethyl	1.13	2298.45	572.0623	-0.415
Me	OMe	4-*iso*propyl	0.99	2277.66	727.0815	-0.232
CH_2CCl_3	OMe	4-*iso*propyl	0.17	2289.52	573.0793	-0.232
CH_2CCl_3	Br	4-ethyl	1.21	2298.77	594.1926	-0.415

MATLAB script used for model development:

```
%
clear all;
close all;
clc;
data=[1.923816286   2282.73 722.8714    -0.415
1.478687111 2293.4  670.4399    -0.415
0.412189357 2287.76 717.3972    -0.232
1.539893941 2297.98 642.6425    -0.415
0.965896925 2294.67 647.7295    -0.232
0.377468163 2297.98 642.6425    -0.232
0.216077643 2304.36 599.2573    -0.232
1.4110261   2295.14 598.3918    -0.415
1.007342637 2304.59 552.1165    -0.415
-0.476461518    2298.77 594.1926    -0.232
1.647173678 2286.75 702.7315    -0.415
1.611628641 2288.37 704.0457    -0.415
```

```
1.128871134 2298.45 572.0623    -0.415
0.99382244  2277.66 727.0815    -0.232
0.168486566 2289.52 573.0793    -0.232
1.211849055 2298.77 594.1926    -0.415];
stdr = nanstd(data);
means = nanmean(data);
sr1 = data-repmat(means,size(data,1),1);
sr = sr1./repmat(stdr,size(data,1),1);
%sr = data./repmat(stdr,size(data,1),1);
Xz=sr(1:10,2:4);
Yz=sr(1:10,1);
X=data(1:10,2:4);
Y=data(1:10,1);
%%
FitForward = LinearModel.stepwise(Xz, Yz)
FitBackL = LinearModel.stepwise(Xz, Yz, 'linear')
FitBackI = LinearModel.stepwise(Xz, Yz, 'interactions')
FitQuad = LinearModel.stepwise(Xz, Yz, 'purequadratic')
```

All four stepwise algorithms in the above MATLAB script afforded the model y ~ 1 + x2 + x3 (Non-normalized: $\mathbf{\Delta\Delta G^{\ddagger}_{DOSP}} = -4.39 + 0.005\mathbf{i_{diazo}} - 6.49\mathbf{q}$; Normalized: $\mathbf{\Delta\Delta G^{\ddagger}_{DOSP}} = 0.45\mathbf{i_{diazo}} - 0.92\mathbf{q}$). External validation of this model is demonstrated in Figure 5a.

Development of Model for Rh_2(*R*-BPCP)$_4$ (Figure 5b)

The training and external validation sets that were used for model development and validation are given in Table S6.

Table S6. Training and External Validation Sets for Rh_2(*R*-BPCP)$_4$ Model Development.

Ester, R	*Aryl, R'*	*Toluene Substrate*	*$\Delta\Delta G^{\ddagger}$ (y)*	*v_{diazo} (x1)*	*i_{diazo} (x2)*	*q (x3)*
**Training Set**						
Me	*t*Bu	4-ethyl	-0.81	2282.73	722.8714	-0.415
Me	CF_3	4-ethyl	-1.03	2293.40	670.4399	-0.415
Me	Br	4-*iso*propyl	-2.83	2287.76	717.3972	-0.232
CH_2CF_3	Br	4-ethyl	-1.01	2297.98	642.6425	-0.415
CH_2CF_3	*t*Bu	4-*iso*propyl	-2.87	2294.67	647.7295	-0.232
CH_2CF_3	Br	4-*iso*propyl	-3.24	2297.98	642.6425	-0.232
CH_2CF_3	CF_3	4-*iso*propyl	-3.33	2304.36	599.2573	-0.232
CH_2CCl_3	*t*Bu	4-ethyl	-1.43	2295.14	598.3918	-0.415
CH_2CCl_3	CF_3	4-ethyl	-1.64	2304.59	552.1165	-0.415
CH_2CCl_3	Br	4-*iso*propyl	-3.99	2298.77	594.1926	-0.232
**External Validation Set**						
Me	Cl	4-ethyl	-0.83	2286.75	702.7315	-0.415
CH_2CH_3	Br	4-ethyl	-0.92	2288.37	704.0457	-0.415
CH_2CBr_3	Br	4-ethyl	-1.64	2298.45	572.0623	-0.415

CH_2CCl_3	OMe	4-*iso*propyl	-2.49	2289.52	573.0793	-0.232
CH_2CCl_3	F	4-*iso*propyl	-3.78	2296.69	553.5468	-0.232
CH_2*t*-Bu	Br	4-ethyl	-1.24	2287.20	659.6421	-0.415
CH_2CCl_3	Br	4-ethyl	-1.50	2298.77	594.1926	-0.415
CH_2CCl_3	H	4-ethyl	-0.68	2286.34	670.4157	-0.415
Me	Br	4-ethyl	-0.88	2287.76	717.3972	-0.415

MATLAB script used for model development:

```
%
clear all;
close all;
clc;
data=[-0.813673306  2282.73 722.8714    -0.415
-1.025327419    2293.4  670.4399    -0.415
-2.832129568    2287.76 717.3972    -0.232
-1.013250998    2297.98 642.6425    -0.415
-2.870217968    2294.67 647.7295    -0.232
-3.236455785    2297.98 642.6425    -0.232
-3.334274164    2304.36 599.2573    -0.232
-1.432014848    2295.14 598.3918    -0.415
-1.641272365    2304.59 552.1165    -0.415
-3.988633681    2298.77 594.1926    -0.232
-0.830258719    2286.75 702.7315    -0.415
-0.921433787    2288.37 704.0457    -0.415
-1.641272365    2298.45 572.0623    -0.415
-2.492225213    2289.52 573.0793    -0.232
-3.781208624    2296.69 553.5468    -0.232
-1.242226323    2287.2  659.6421    -0.415
-1.496918027    2298.77 594.1926    -0.415
-0.683244721    2286.34 670.4157    -0.415
-0.877515582    2287.76 717.3972    -0.415];
stdr = nanstd(data);
means = nanmean(data);
sr1 = data-repmat(means,size(data,1),1);
sr = sr1./repmat(stdr,size(data,1),1);
%sr = data./repmat(stdr,size(data,1),1);
Xz=sr(1:10,2:4);
Yz=sr(1:10,1);
X=data(1:10,2:4);
Y=data(1:10,1);
%%
FitForward = LinearModel.stepwise(Xz, Yz)
FitBackL = LinearModel.stepwise(Xz, Yz, 'linear')
FitBackI = LinearModel.stepwise(Xz, Yz, 'interactions')
FitQuad = LinearModel.stepwise(Xz, Yz, 'purequadratic')
```

All four stepwise algorithms in the above MATLAB script afforded the model y ~ 1 + x2 + x3 (Non-normalized: $\mathbf{\Delta\Delta G^{\ddagger}_{BPCP}}$ = -9.66 + 0.006$\mathbf{i_{diazo}}$ – 11.39$\mathbf{q}$; Normalized: $\mathbf{\Delta\Delta G^{\ddagger}_{BPCP}}$ = -0.04 + 0.31$\mathbf{i_{diazo}}$ – 0.93$\mathbf{q}$). External validation of this model is demonstrated in Figure 5b.

Development of Comprehensive Model for Rh_2(*S*-DOSP)$_4$ and Rh_2(*R*-BPCP)$_4$ (Figure 5c)

The training and external validation sets that were used for model development and validation are given in Table S7.

Table S7. Training and External Validation Sets for Development of Comprehensive Model.

Ester, R	*Aryl, R'*	*Toluene Substrate*	*$\Delta\Delta G^{\ddagger}$ (y)*	i_{diazo} *(x1)*	*q (x2)*	*C* (x3)*
**Training Set**						
Me	*t*Bu	4-ethyl	-0.81	722.8714	-0.415	-1
Me	CF_3	4-ethyl	-1.03	670.4399	-0.415	-1
Me	Br	4-*iso*propyl	-2.83	717.3972	-0.232	-1
CH_2CF_3	Br	4-ethyl	-1.01	642.6425	-0.415	-1
CH_2CF_3	*t*Bu	4-*iso*propyl	-2.87	647.7295	-0.232	-1
CH_2CF_3	Br	4-*iso*propyl	-3.24	642.6425	-0.232	-1
CH_2CF_3	CF_3	4-*iso*propyl	-3.33	599.2573	-0.232	-1
CH_2CCl_3	*t*Bu	4-ethyl	-1.43	598.3918	-0.415	-1
CH_2CCl_3	CF_3	4-ethyl	-1.64	552.1165	-0.415	-1
CH_2CCl_3	Br	4-*iso*propyl	-3.99	594.1926	-0.232	-1
Me	*t*Bu	4-ethyl	1.92	722.8714	-0.415	1
Me	CF_3	4-ethyl	1.48	670.4399	-0.415	1
Me	Br	4-*iso*propyl	0.41	717.3972	-0.232	1
CH_2CF_3	Br	4-ethyl	1.54	642.6425	-0.415	1
CH_2CF_3	*t*Bu	4-*iso*propyl	0.97	647.7295	-0.232	1
CH_2CF_3	Br	4-*iso*propyl	0.38	642.6425	-0.232	1
CH_2CF_3	CF_3	4-*iso*propyl	0.22	599.2573	-0.232	1
CH_2CCl_3	*t*Bu	4-ethyl	1.41	598.3918	-0.415	1
CH_2CCl_3	CF_3	4-ethyl	1.01	552.1165	-0.415	1
CH_2CCl_3	Br	4-*iso*propyl	-0.48	594.1926	-0.232	1
**External Validation Set**						
Me	Cl	4-ethyl	-0.83	702.7315	-0.415	-1
CH_2CH_3	Br	4-ethyl	-0.92	704.0457	-0.415	-1
CH_2CBr_3	Br	4-ethyl	-1.64	572.0623	-0.415	-1
CH_2CCl_3	OMe	4-*iso*propyl	-2.49	573.0793	-0.232	-1
CH_2CCl_3	F	4-*iso*propyl	-3.78	553.5468	-0.232	-1
CH_2*t*-Bu	Br	4-ethyl	-1.24	659.6421	-0.415	-1
CH_2CCl_3	Br	4-ethyl	-1.50	594.1926	-0.415	-1
CH_2CCl_3	H	4-ethyl	-0.68	670.4157	-0.415	-1
Me	Br	4-ethyl	-0.88	717.3972	-0.415	-1
Me	Cl	4-ethyl	1.65	702.7315	-0.415	1

CH_2CH_3	Br	4-ethyl	1.61	704.0457	-0.415	1
CH_2CBr_3	Br	4-ethyl	1.13	572.0623	-0.415	1
Me	OMe	4-*iso*propyl	0.99	727.0815	-0.232	1
CH_2CCl_3	OMe	4-*iso*propyl	0.17	573.0793	-0.232	1
CH_2CCl_3	Br	4-ethyl	1.21	594.1926	-0.415	1

* *Rh_2(S-DOSP)$_4$ = 1; Rh_2(R-BPCP)$_4$ = -1*

MATLAB script used for model development:

```
%
clear all;
close all;
clc;
data=[1.923816286   722.8714    -0.415  1
1.478687111 670.4399    -0.415  1
0.412189357 717.3972    -0.232  1
1.539893941 642.6425    -0.415  1
0.965896925 647.7295    -0.232  1
0.377468163 642.6425    -0.232  1
0.216077643 599.2573    -0.232  1
1.4110261   598.3918    -0.415  1
1.007342637 552.1165    -0.415  1
-0.476461518    594.1926    -0.232  1
-0.813673306    722.8714    -0.415  -1
-1.025327419    670.4399    -0.415  -1
-2.832129568    717.3972    -0.232  -1
-1.013250998    642.6425    -0.415  -1
-2.870217968    647.7295    -0.232  -1
-3.236455785    642.6425    -0.232  -1
-3.334274164    599.2573    -0.232  -1
-1.432014848    598.3918    -0.415  -1
-1.641272365    552.1165    -0.415  -1
-3.988633681    594.1926    -0.232  -1
1.647173678 702.7315    -0.415  1
1.611628641 704.0457    -0.415  1
1.128871134 572.0623    -0.415  1
0.99382244  727.0815    -0.232  1
0.168486566 573.0793    -0.232  1
1.211849055 594.1926    -0.415  1
-0.830258719    702.7315    -0.415  -1
-0.921433787    704.0457    -0.415  -1
-1.641272365    572.0623    -0.415  -1
-2.492225213    573.0793    -0.232  -1
-3.781208624    553.5468    -0.232  -1
-1.244752729    659.6421    -0.415  -1
-1.496918027    594.1926    -0.415  -1
```

```
-0.683244721    670.4157    -0.415  -1
-0.877515582    717.3972    -0.415  -1];
stdr = nanstd(data);
means = nanmean(data);
sr1 = data-repmat(means,size(data,1),1);
sr = sr1./repmat(stdr,size(data,1),1);
%sr = data./repmat(stdr,size(data,1),1);
Xz=sr(1:20,2:4);
Yz=sr(1:20,1);
X=data(1:20,2:4);
Y=data(1:20,1);
%%
FitForward = LinearModel.stepwise(Xz, Yz)
FitBackL = LinearModel.stepwise(Xz, Yz, 'linear')
FitBackI = LinearModel.stepwise(Xz, Yz, 'interactions')
FitQuad = LinearModel.stepwise(Xz, Yz, 'purequadratic')
%% y ~ 1 + x1 + x2 + x3 + x2:x3 was consistently identified for each of the above four algorithms, yielding R^2=-1.02
```

All four stepwise algorithms in the above MATLAB script afforded the model y ~ 1 + x1 + x2 + x3 + x2:x3 (Non-normalized: $\mathbf{\Delta\Delta G^{\ddagger}_{Comprehensive}}$ = -7.02 + 0.005$\mathbf{i}_{\mathbf{diazo}}$ – 8.94$\mathbf{q}$ + 2.34$\boldsymbol{C}$ + 2.44$\boldsymbol{q}$*$\boldsymbol{C}$; Normalized: $\mathbf{\Delta\Delta G^{\ddagger}_{Comprehensive}}$ = -0.02 + 0.18$\mathbf{i}_{\mathbf{diazo}}$ – 0.48$\mathbf{q}$ + 0.88$\boldsymbol{C}$ + 0.13$\boldsymbol{q}$*$\boldsymbol{C}$). External validation of this model is demonstrated in Figure 5c.

6 – NMR Spectra

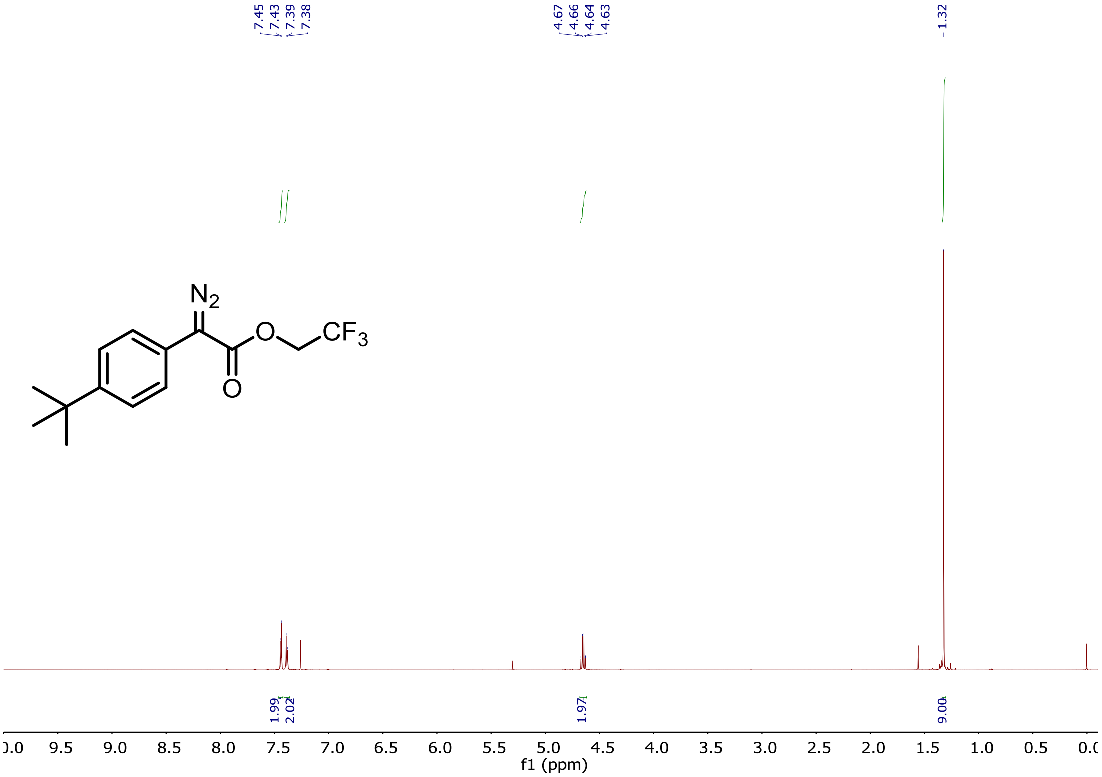
7.45
7.43
7.39
7.38
4.67
4.66
4.64
4.63
1.32
N2
O
CF3
O
1.99
2.02
1.97
9.00
f1 (ppm)

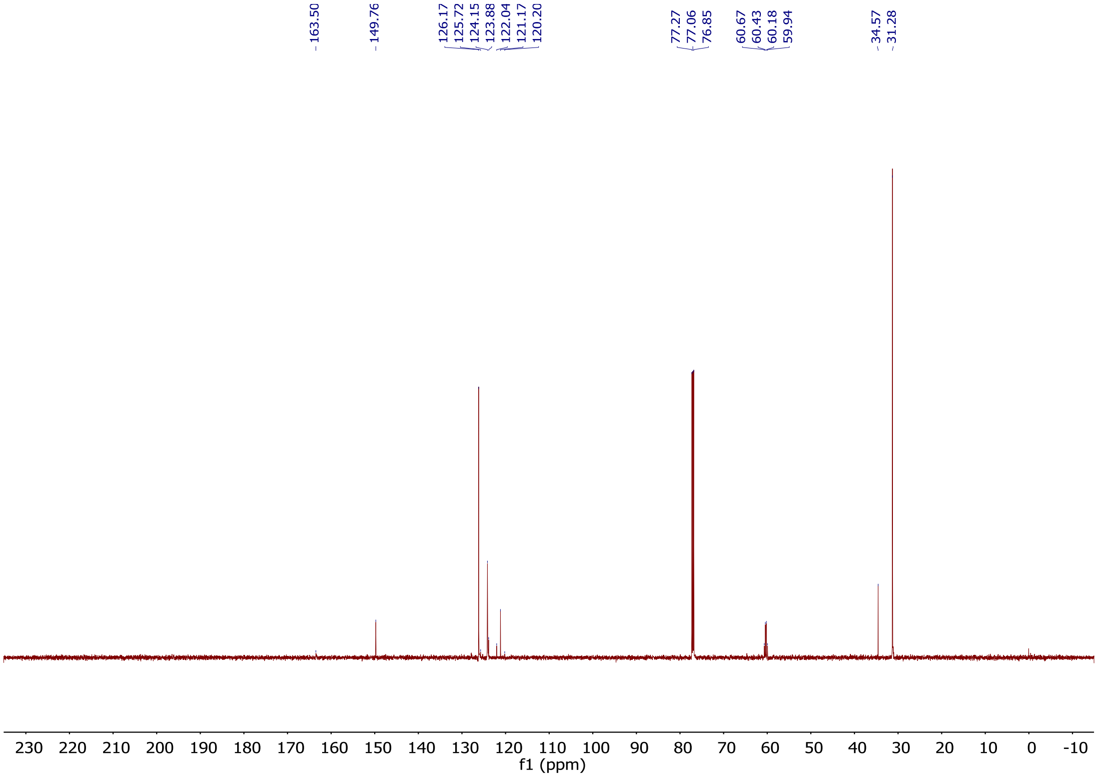
163.50
149.76
126.17
125.72
124.15
123.88
122.04
121.17
120.20
77.27
77.06
76.85
60.67
60.43
60.18
59.94
34.57
31.28
f1 (ppm)

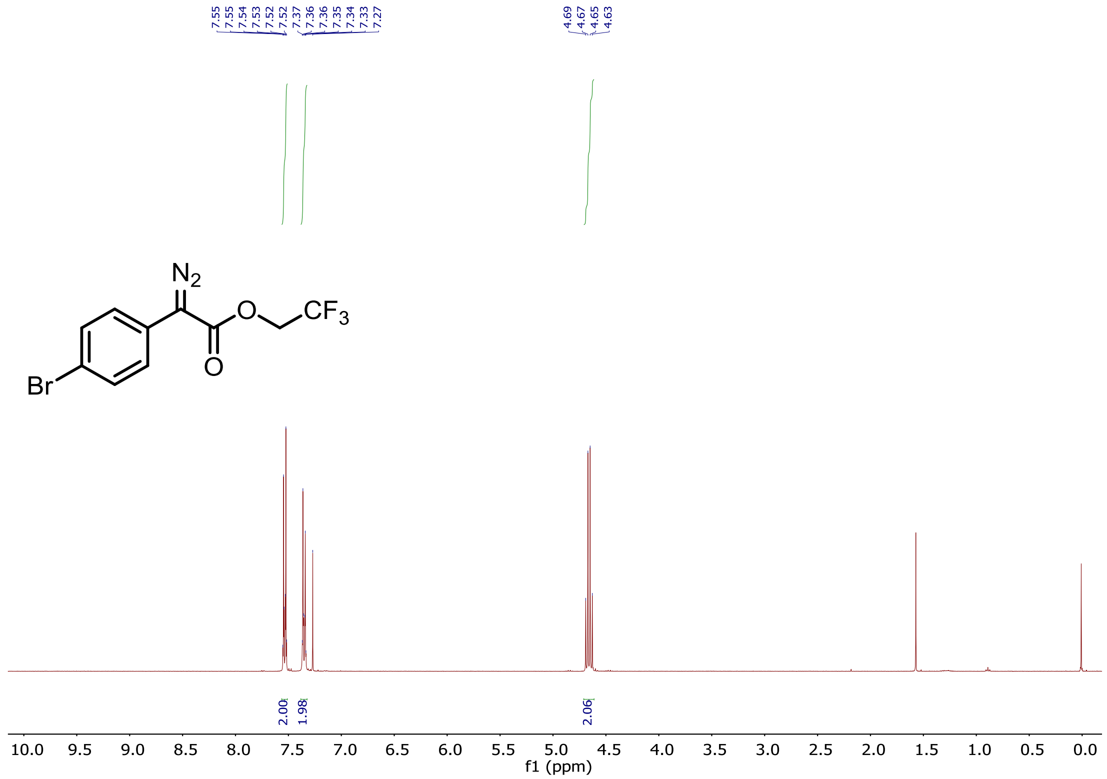
7.55
7.55
7.54
7.53
7.52
7.52
7.37
7.36
7.36
7.35
7.34
7.33
7.27
4.69
4.67
4.65
4.63
N2
O
CF3
Br
O
2.00
1.98
2.06
10.0
9.5
9.0
8.5
8.0
7.5
7.0
6.5
6.0
5.5
5.0
4.5
4.0
3.5
3.0
2.5
2.0
1.5
1.0
0.5
0.0
f1 (ppm)

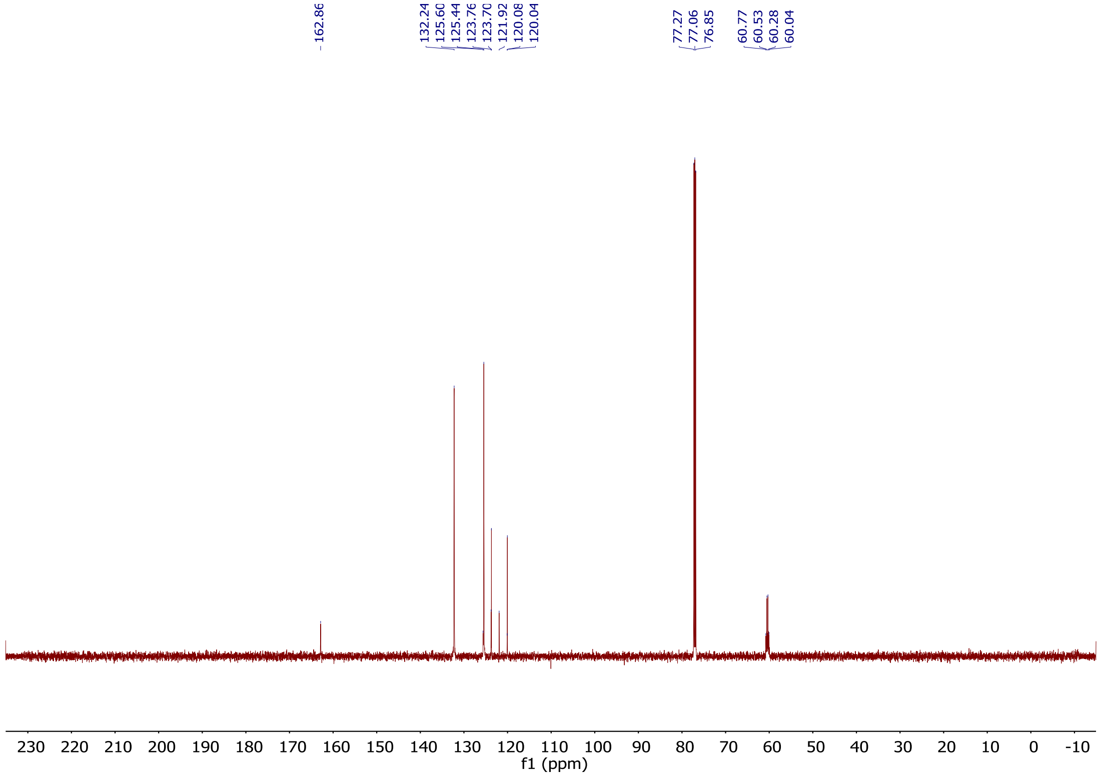
162.86
132.24
125.60
125.44
123.76
123.70
121.92
120.08
120.04
77.27
77.06
76.85
60.77
60.53
60.28
60.04
230 220 210 200 190 180 170 160 150 140 130 120 110 100 90 80 70 60 50 40 30 20 10 0 -10
f1 (ppm)

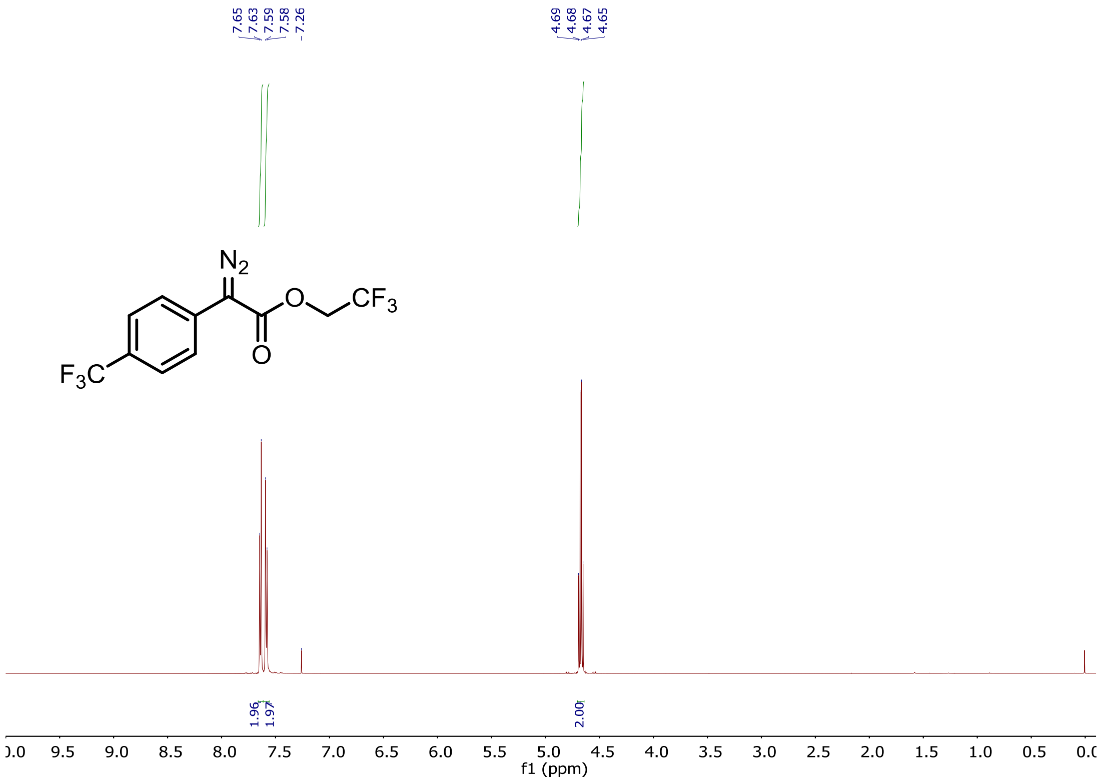
7.65
7.63
7.59
7.58
7.26
4.69
4.68
4.67
4.65
N2
O
CF3
O
F3C
1.96
1.97
2.00
0.0 9.5 9.0 8.5 8.0 7.5 7.0 6.5 6.0 5.5 5.0 4.5 4.0 3.5 3.0 2.5 2.0 1.5 1.0 0.5 0.0
f1 (ppm)

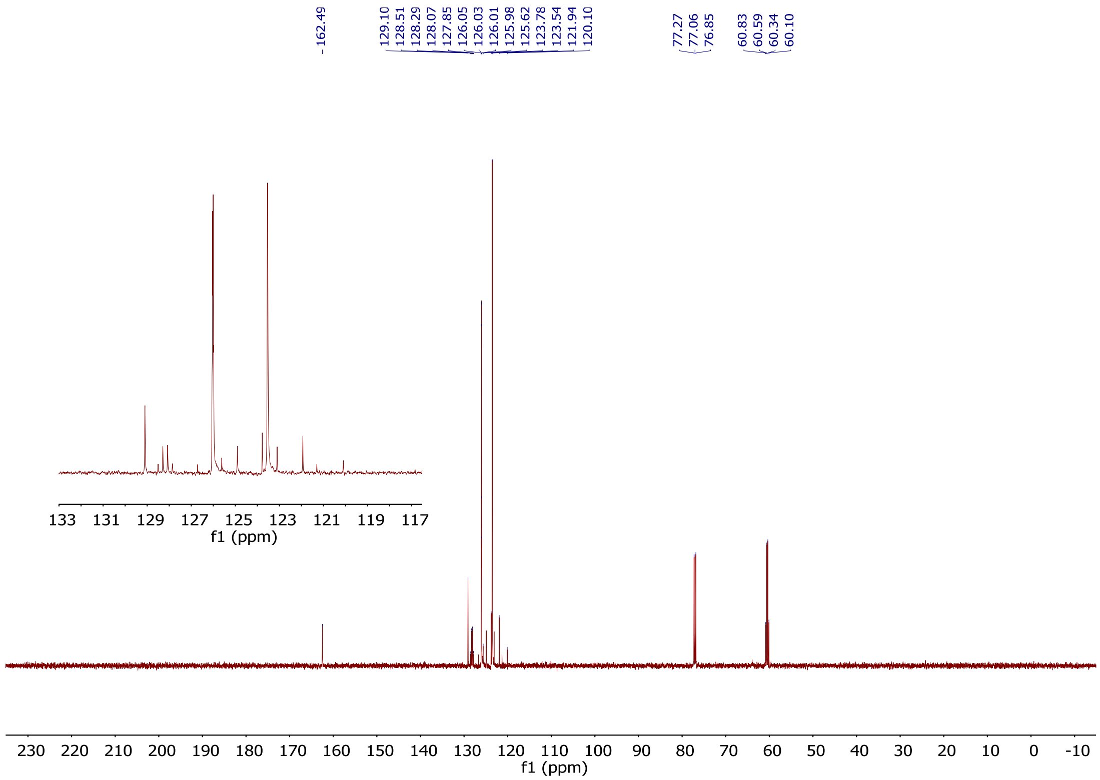
162.49
129.10
128.51
128.29
128.07
127.85
126.05
126.03
126.01
125.98
125.62
123.78
123.54
121.94
120.10
77.27
77.06
76.85
60.83
60.59
60.34
60.10
133 131 129 127 125 123 121 119 117
f1 (ppm)
230 220 210 200 190 180 170 160 150 140 130 120 110 100 90 80 70 60 50 40 30 20 10 0 -10
f1 (ppm)

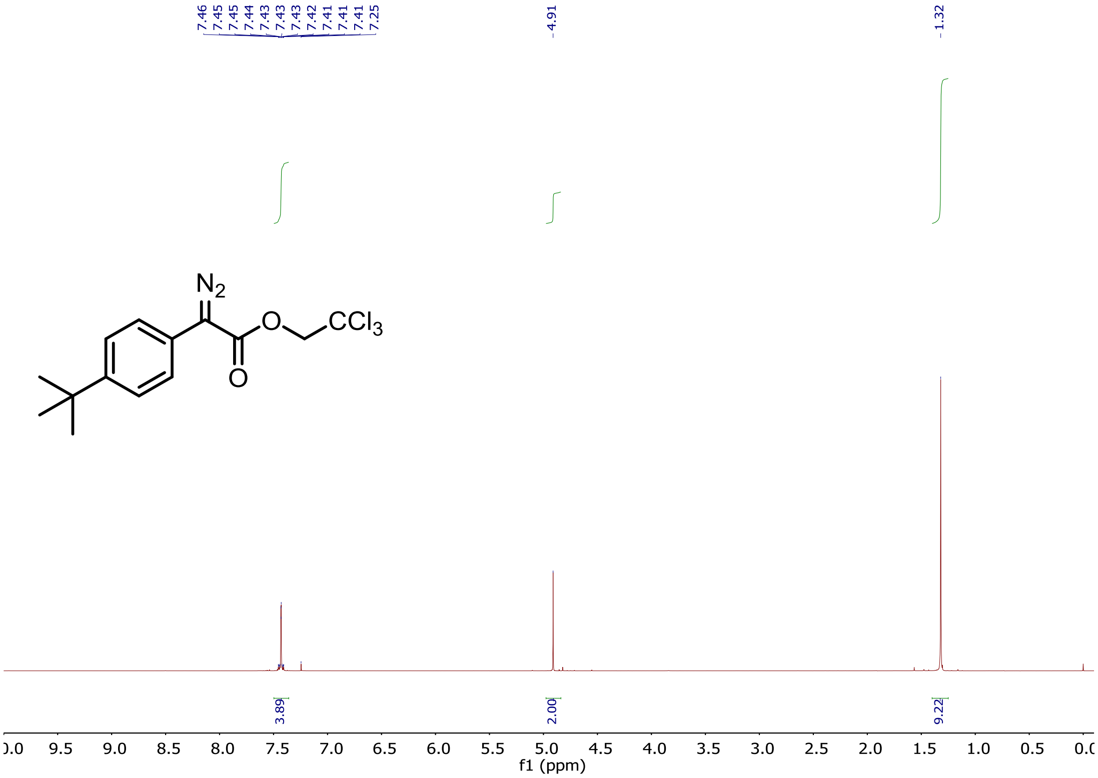
7.46
7.45
7.45
7.44
7.43
7.43
7.42
7.41
7.41
7.41
7.25
4.91
1.32
N2
O
CCl3
O
3.89
2.00
9.22
f1 (ppm)

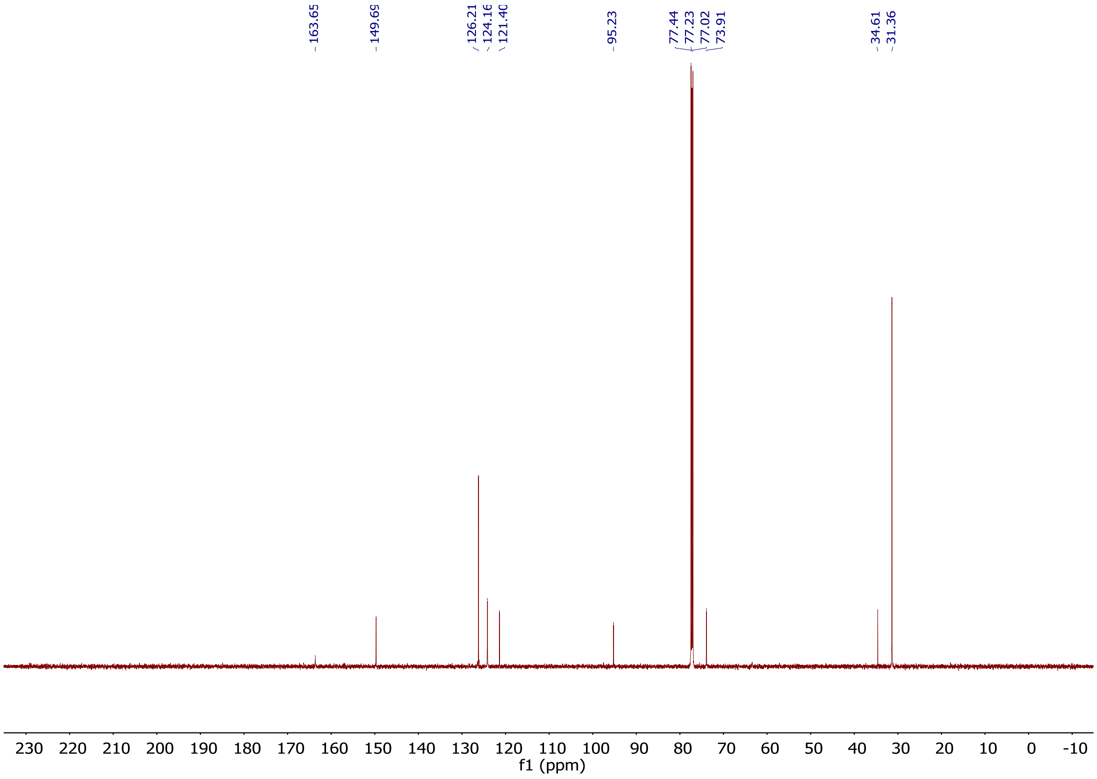
163.65
149.69
126.21
124.16
121.40
95.23
77.44
77.23
77.02
73.91
34.61
31.36
f1 (ppm)

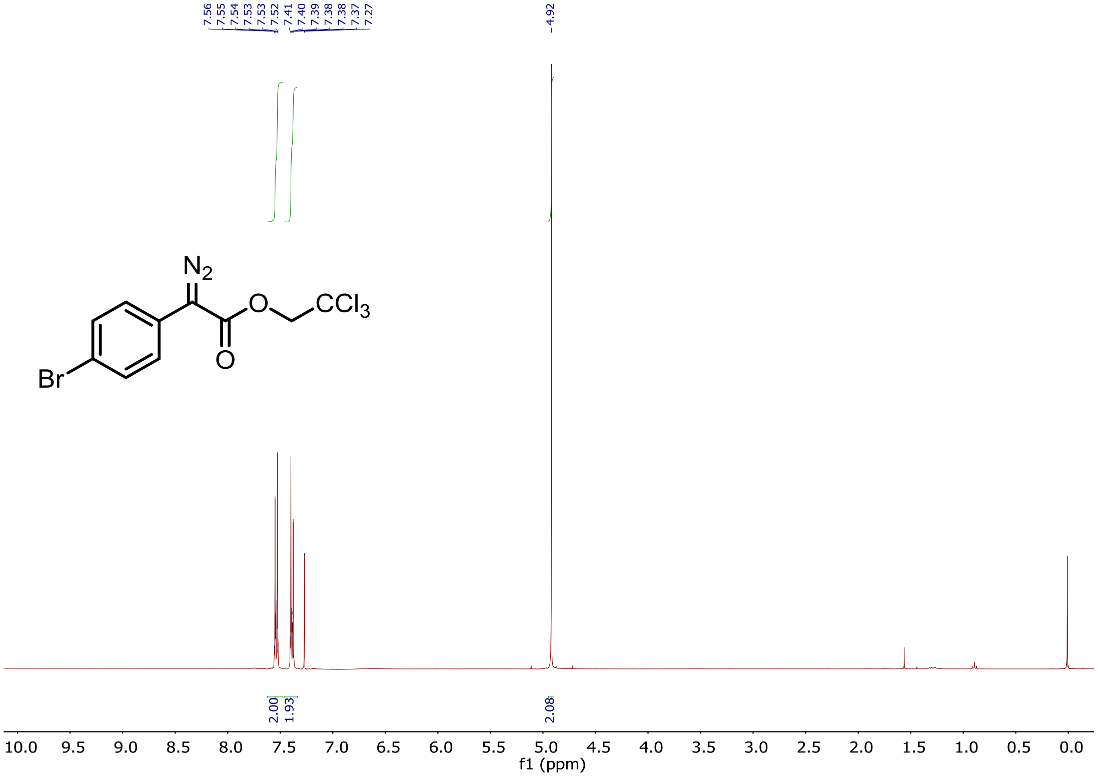
7.56
7.55
7.54
7.53
7.53
7.52
7.41
7.40
7.39
7.38
7.38
7.37
7.27
4.92
N2
O
CCl3
Br
O
2.00
1.93
2.08
10.0 9.5 9.0 8.5 8.0 7.5 7.0 6.5 6.0 5.5 5.0 4.5 4.0 3.5 3.0 2.5 2.0 1.5 1.0 0.5 0.0
f1 (ppm)

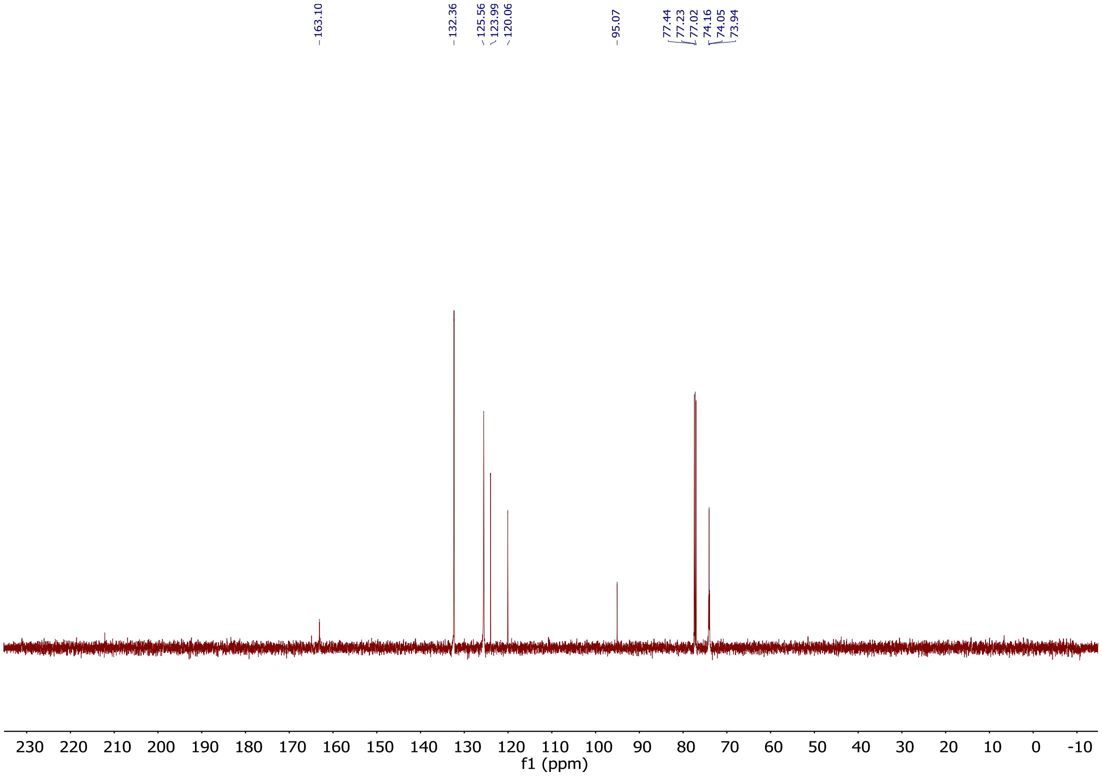

163.10
132.36
125.56
123.99
120.06
95.07
77.44
77.23
77.02
74.16
74.05
73.94
230 220 210 200 190 180 170 160 150 140 130 120 110 100 90 80 70 60 50 40 30 20 10 0 -10
f1 (ppm)

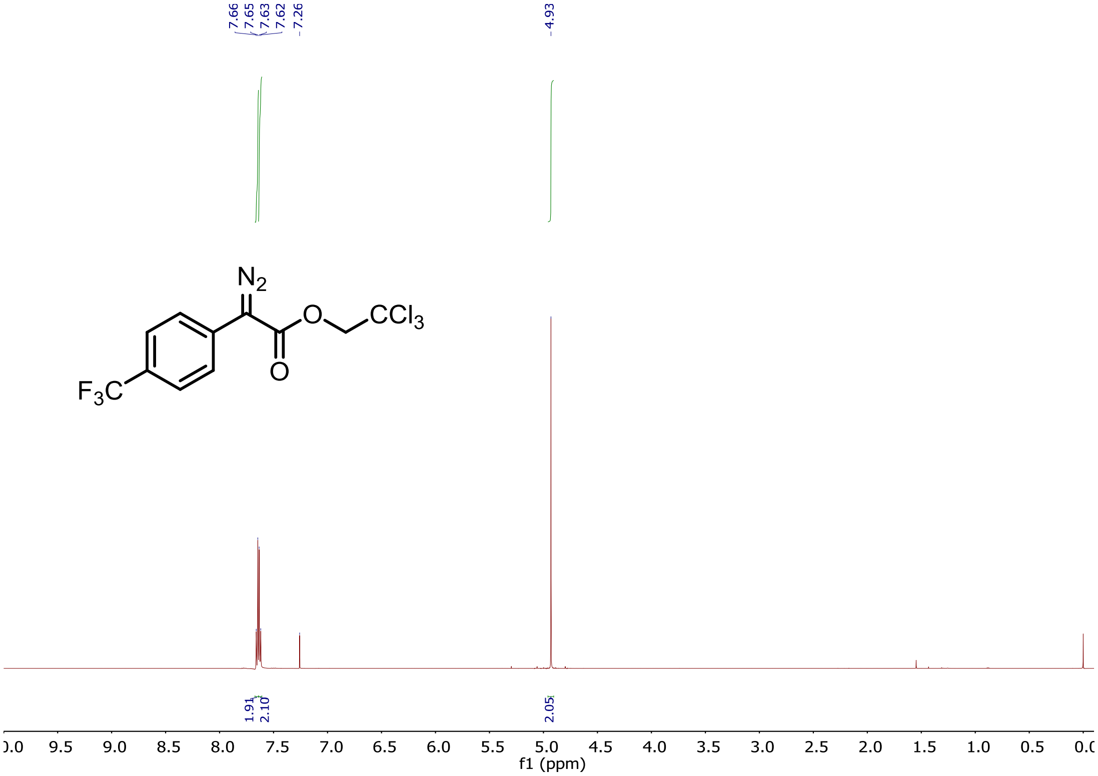
7.66
7.65
7.63
7.62
7.26
4.93
N_2
CCl_3
O
O
F_3C
1.91
2.10
2.05
f1 (ppm)

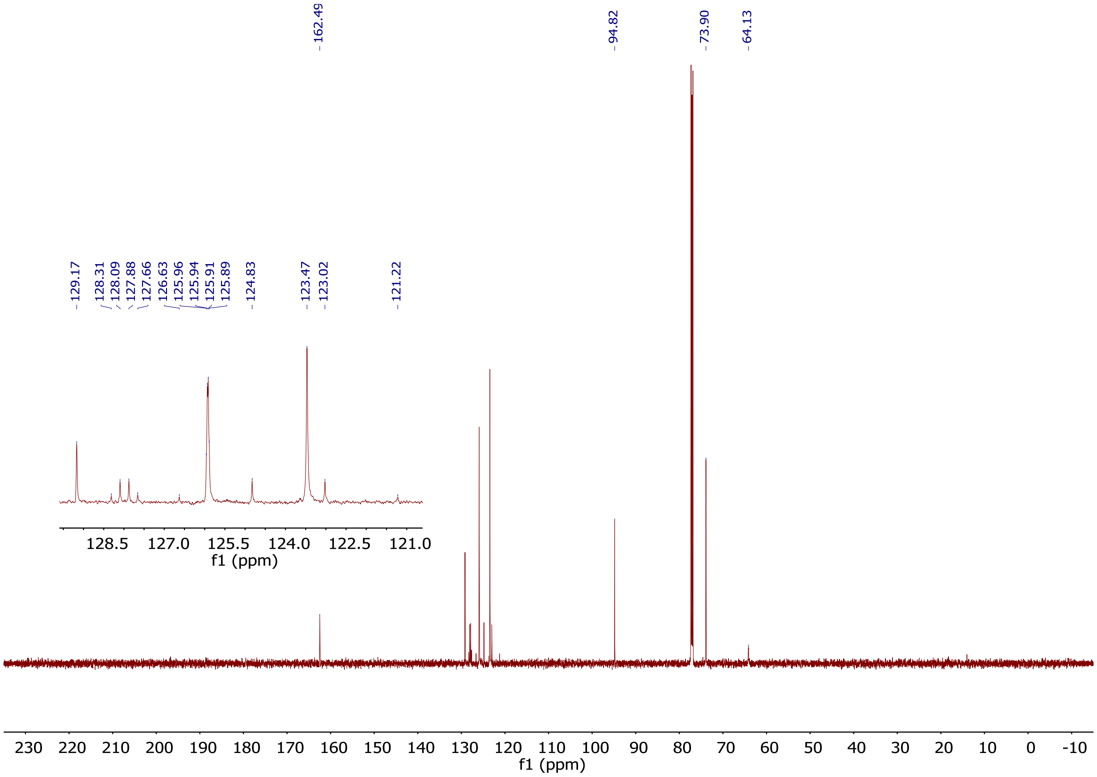
162.49
94.82
73.90
64.13
129.17
128.31
128.09
127.88
127.66
126.63
125.96
125.94
125.91
125.89
124.83
123.47
123.02
121.22
f1 (ppm)
f1 (ppm)

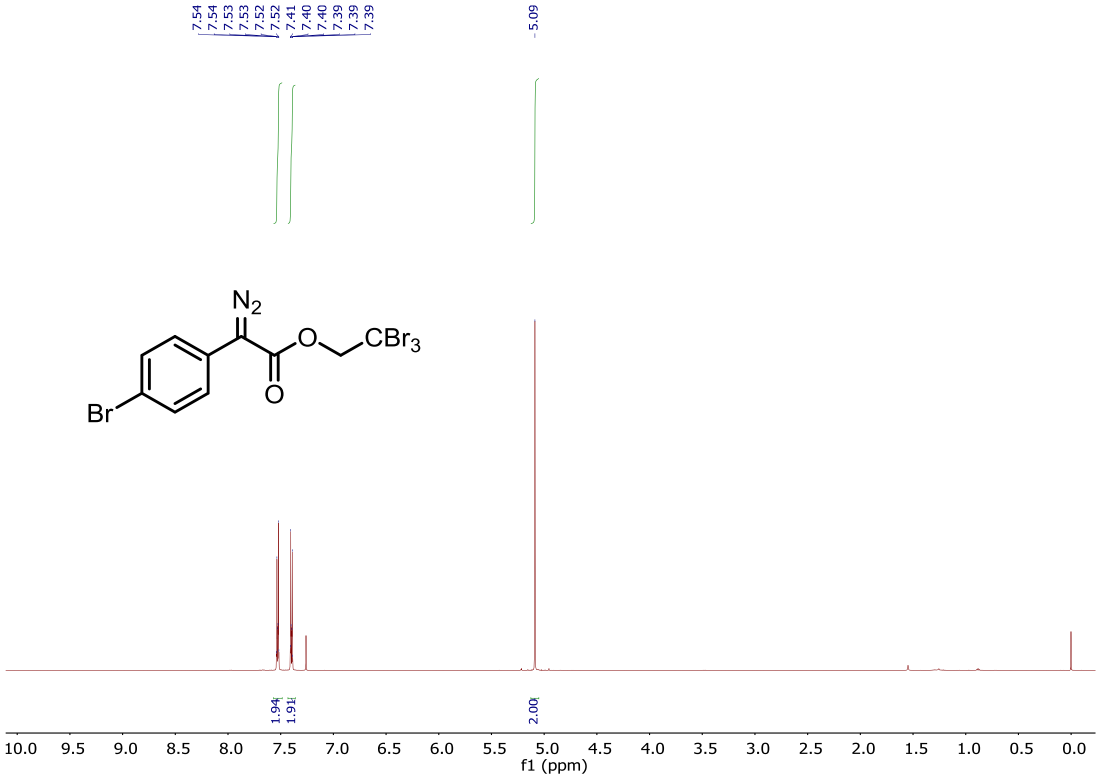
7.54
7.54
7.53
7.53
7.52
7.52
7.41
7.40
7.40
7.39
7.39
7.39
5.09
N2
O
CBr3
Br
O
1.94
1.91
2.00
10.0 9.5 9.0 8.5 8.0 7.5 7.0 6.5 6.0 5.5 5.0 4.5 4.0 3.5 3.0 2.5 2.0 1.5 1.0 0.5 0.0
f1 (ppm)

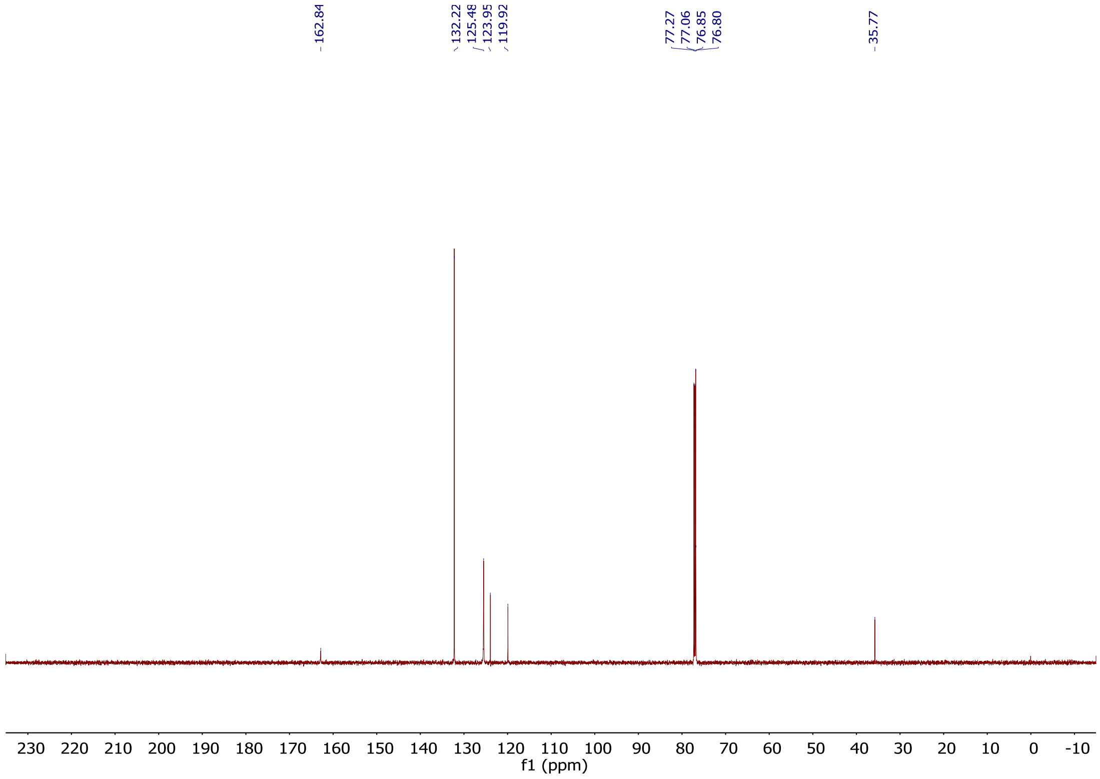
162.84
132.22
125.48
123.95
119.92
77.27
77.06
76.85
76.80
35.77
230 220 210 200 190 180 170 160 150 140 130 120 110 100 90 80 70 60 50 40 30 20 10 0 -10
f1 (ppm)

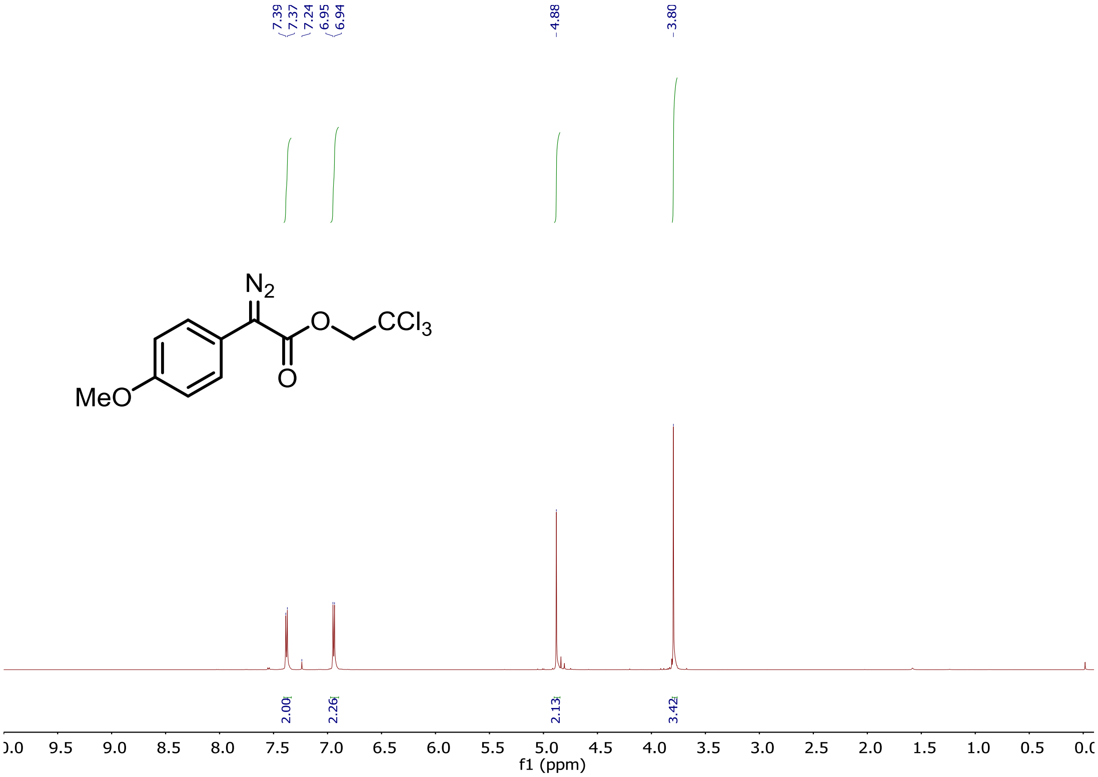
7.39
7.37
7.24
6.95
6.94
4.88
3.80
N2
CCl3
O
MeO
2.00
2.26
2.13
3.42
f1 (ppm)

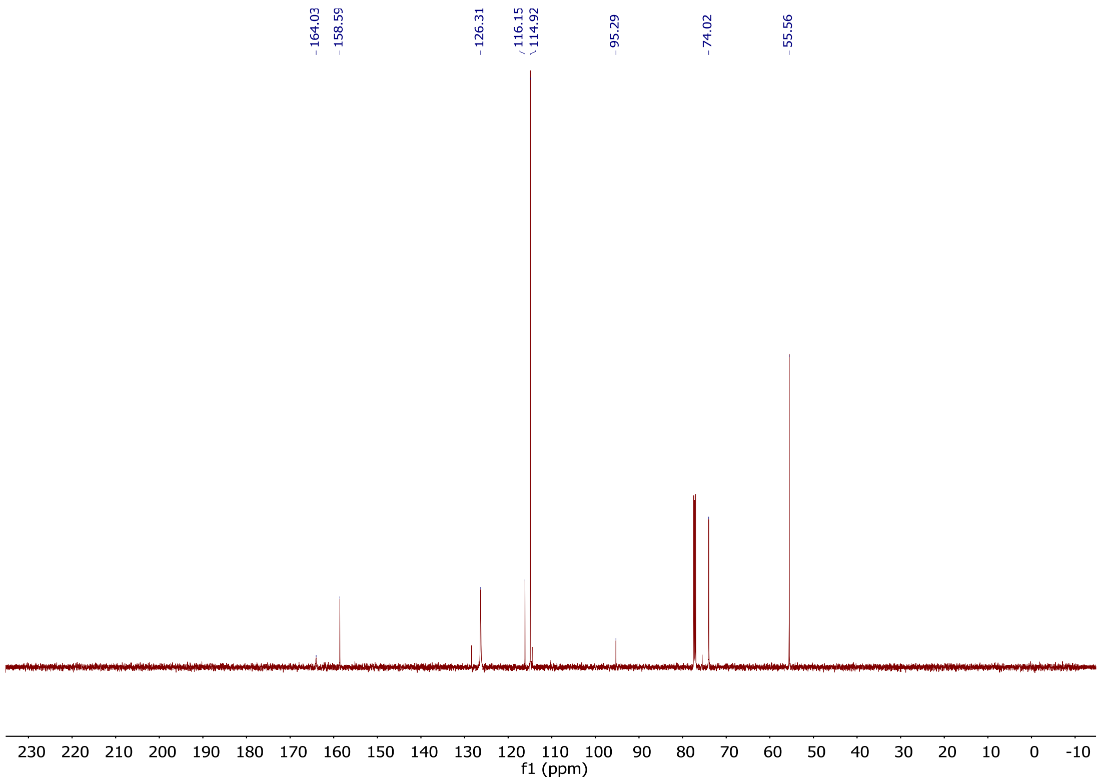
164.03
158.59
126.31
116.15
114.92
95.29
74.02
55.56
230 220 210 200 190 180 170 160 150 140 130 120 110 100 90 80 70 60 50 40 30 20 10 0 -10
f1 (ppm)

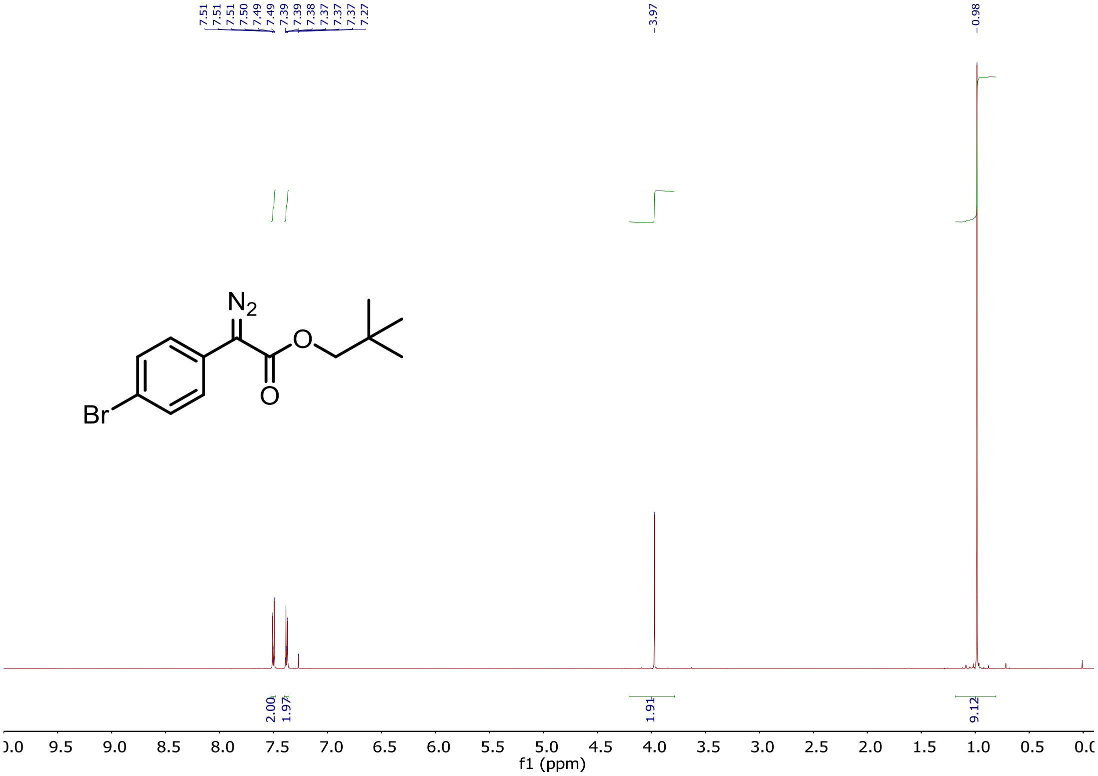
7.51
7.51
7.51
7.50
7.49
7.49
7.39
7.39
7.38
7.37
7.37
7.37
7.27
3.97
0.98
N2
O
O
Br
2.00
1.97
1.91
9.12
f1 (ppm)

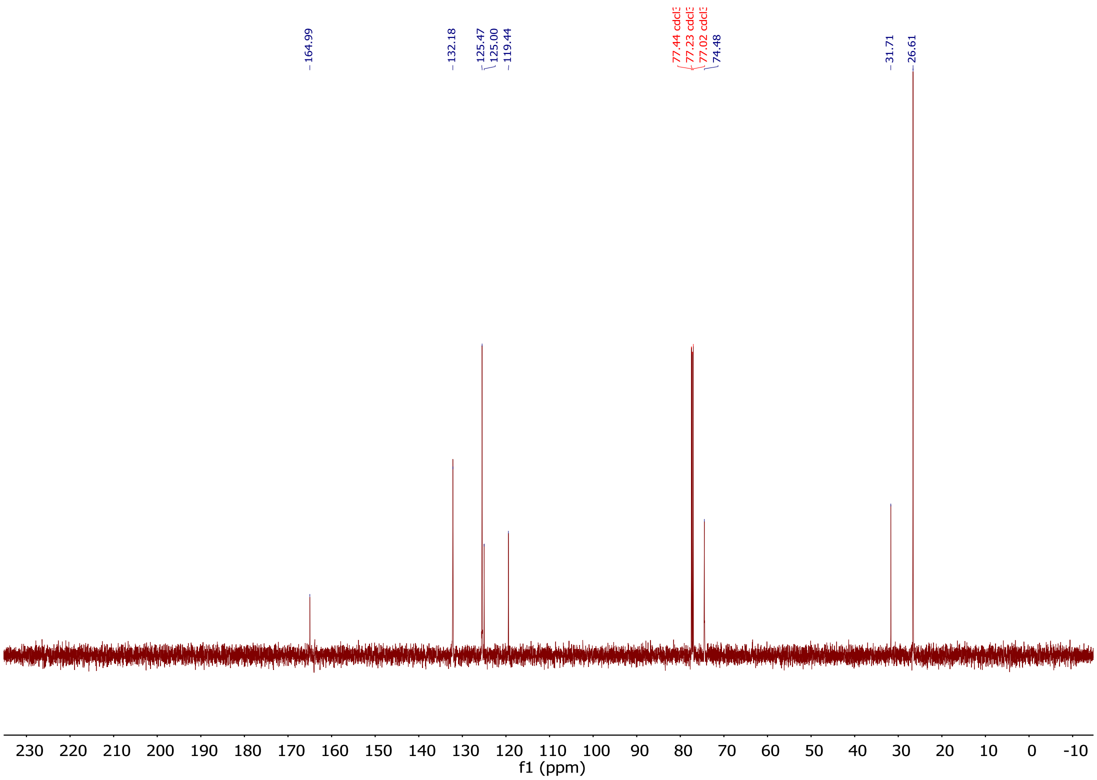
164.99
132.18
125.47
125.00
119.44
77.44 cdcl3
77.23 cdcl3
77.02 cdcl3
74.48
31.71
26.61
f1 (ppm)

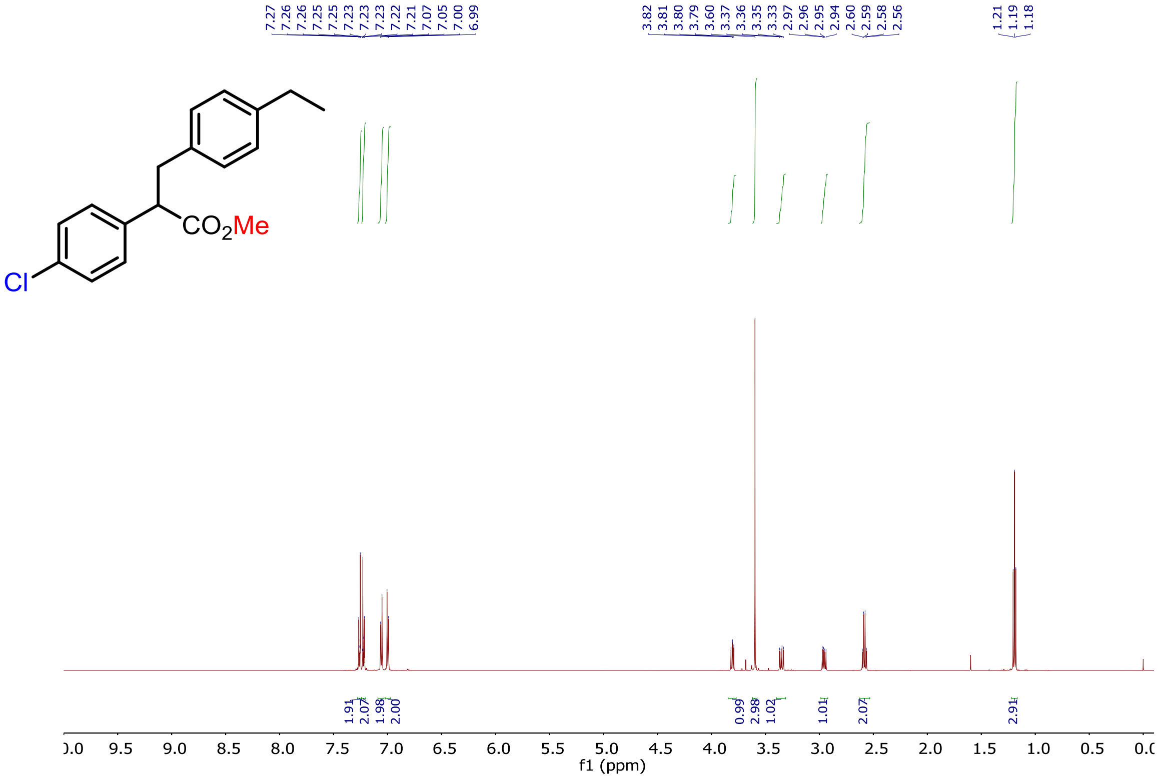

7.27 7.26 7.26 7.25 7.25 7.23 7.23 7.23 7.22 7.21 7.07 7.05 7.00 6.99
3.82 3.81 3.80 3.79 3.60 3.37 3.36 3.35 3.33 2.97 2.96 2.95 2.94 2.60 2.59 2.58 2.56
1.21 1.19 1.18
CO2Me
Cl
1.91 2.07 1.98 2.00
0.99 2.98 1.02
1.01
2.07
2.91
f1 (ppm)

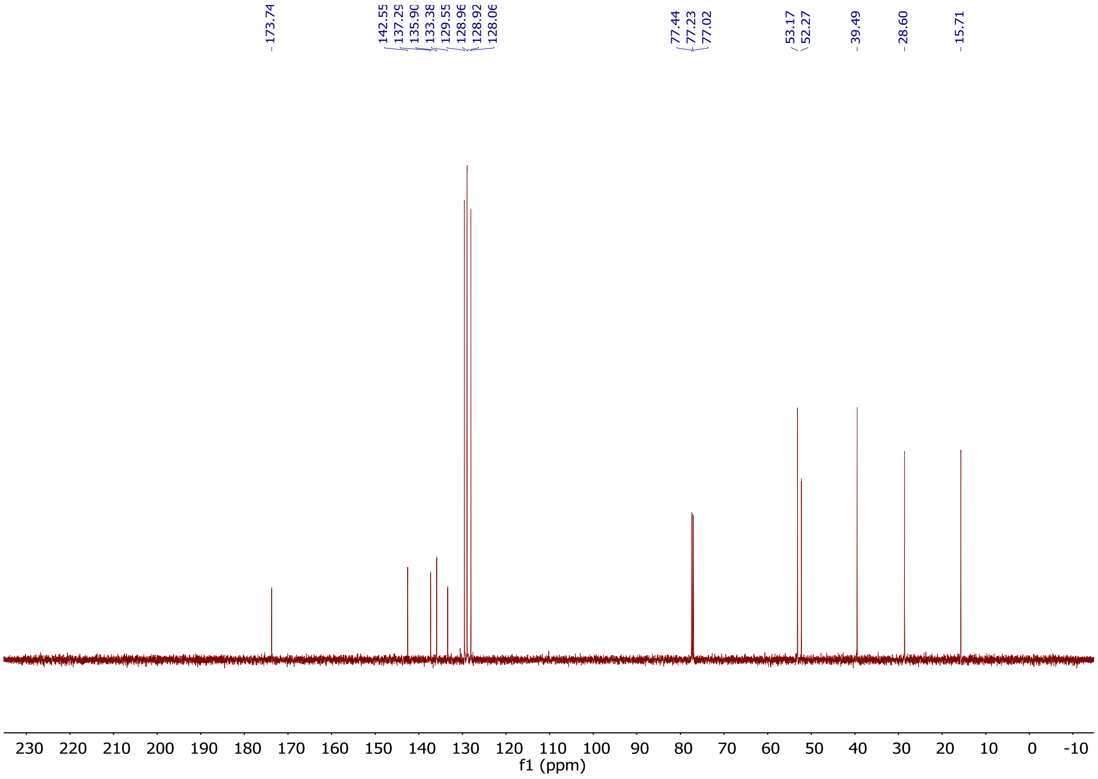

173.74
142.55 137.29 135.90 133.38 129.55 128.96 128.92 128.06
77.44 77.23 77.02
53.17 52.27
39.49
28.60
15.71
f1 (ppm)

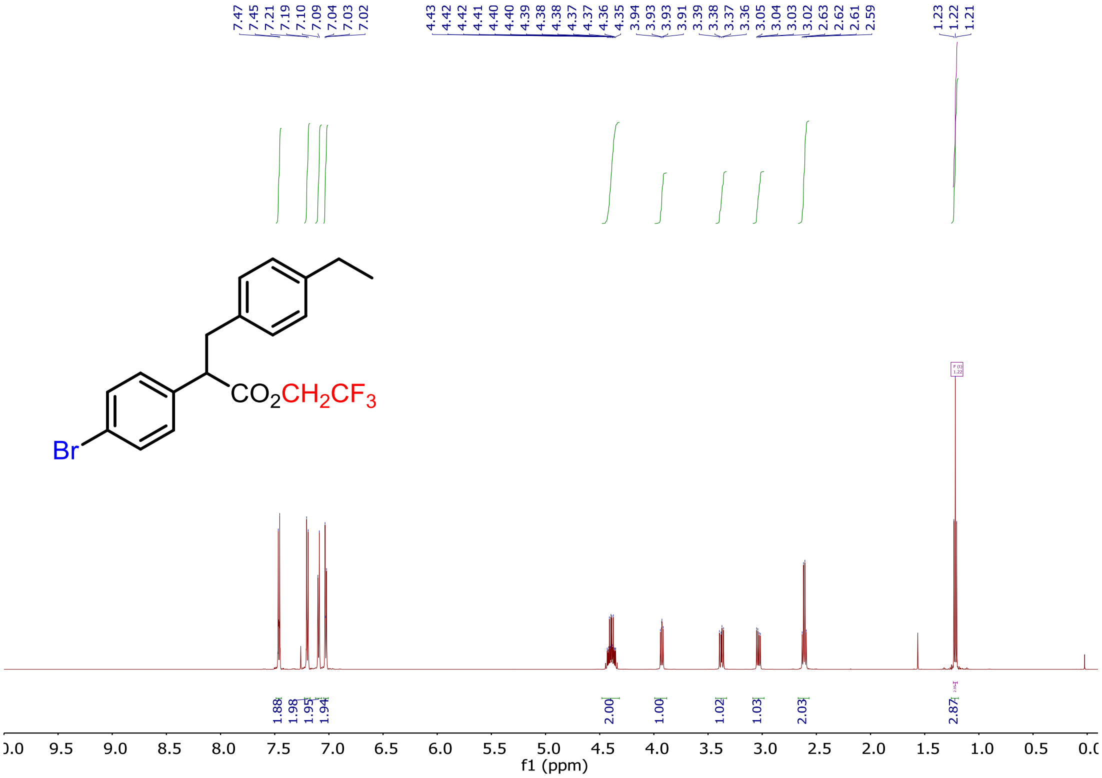
7.47 7.45 7.21 7.19 7.10 7.09 7.04 7.03 7.02
4.43 4.42 4.42 4.41 4.40 4.40 4.39 4.38 4.38 4.37 4.37 4.36 4.35 3.94 3.93 3.93 3.91 3.39 3.38 3.37 3.36 3.05 3.04 3.03 3.02 2.63 2.62 2.61 2.59
1.23 1.22 1.21
CO2CH2CF3
Br
1.88 1.98 1.95 1.94 2.00 1.00 1.02 1.03 2.03 2.87
f1 (ppm)

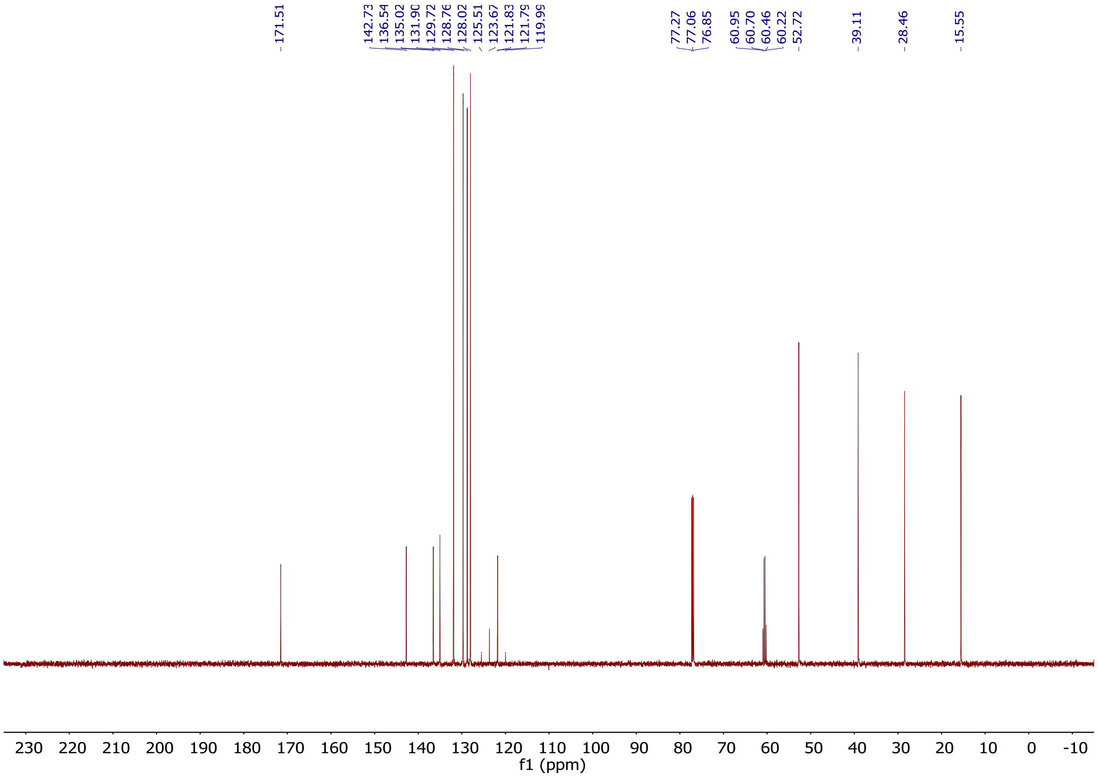
171.51
142.73 136.54 135.02 131.90 129.72 128.76 128.02 125.51 123.67 121.83 121.79 119.99
77.27 77.06 76.85 60.95 60.70 60.46 60.22 52.72
39.11
28.46
15.55
f1 (ppm)

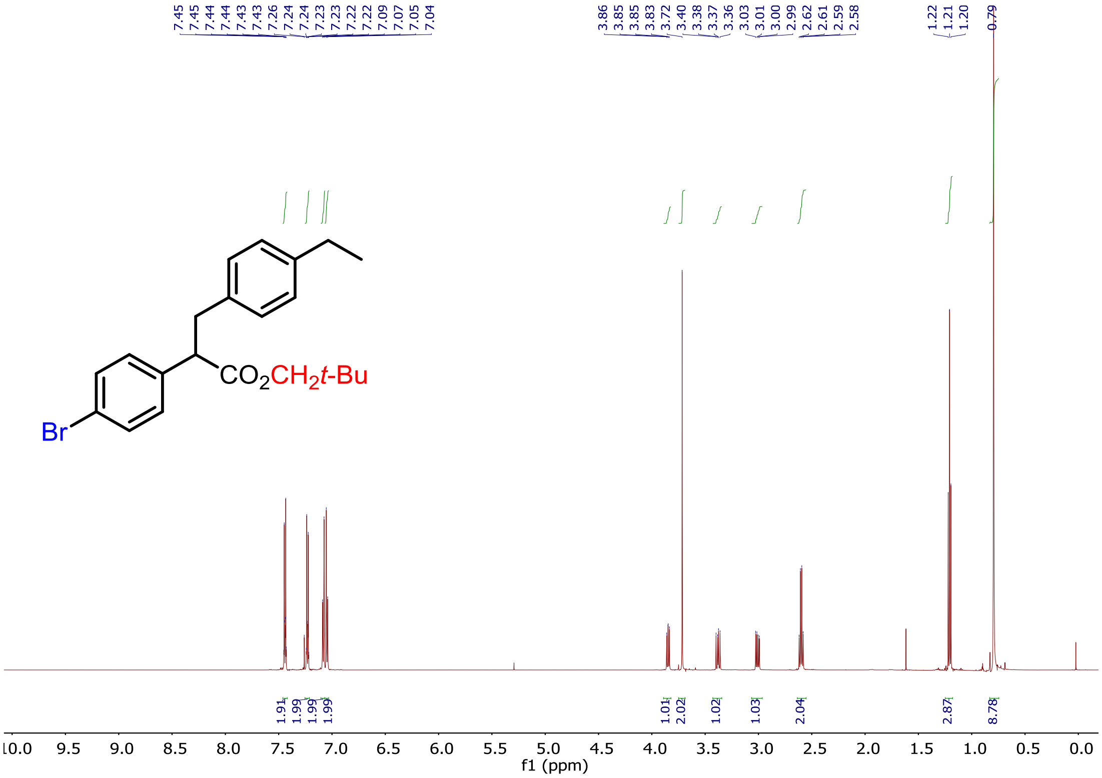
7.45 7.45 7.44 7.44 7.43 7.43 7.26 7.24 7.24 7.23 7.23 7.22 7.22 7.09 7.07 7.05 7.04
3.86 3.85 3.85 3.83 3.72 3.40 3.38 3.37 3.36 3.03 3.01 3.00 2.99 2.62 2.61 2.59 2.58
1.22 1.21 1.20 0.79
Br
CO2CH2t-Bu
1.91 1.99 1.99 1.99
1.01 2.02 1.02 1.03 2.04
2.87 8.78
f1 (ppm)

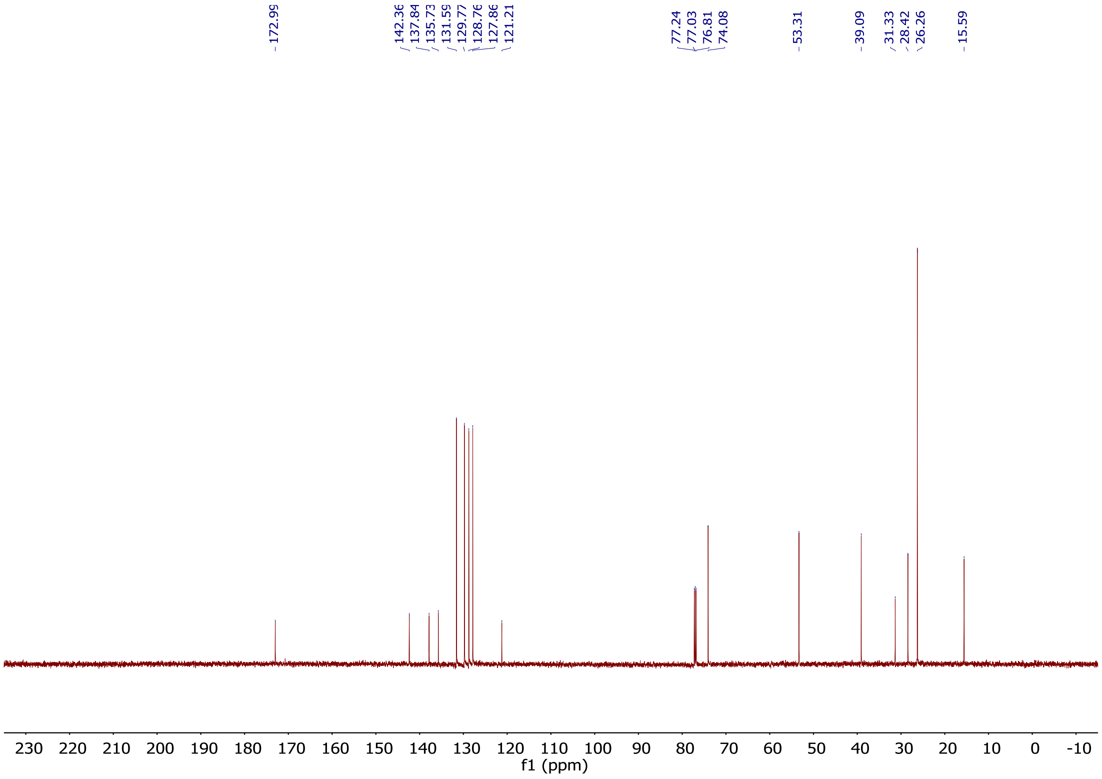
172.99
142.36 137.84 135.73 131.59 129.77 128.76 127.86 121.21
77.24 77.03 76.81 74.08
53.31
39.09 31.33 28.42 26.26
15.59
f1 (ppm)

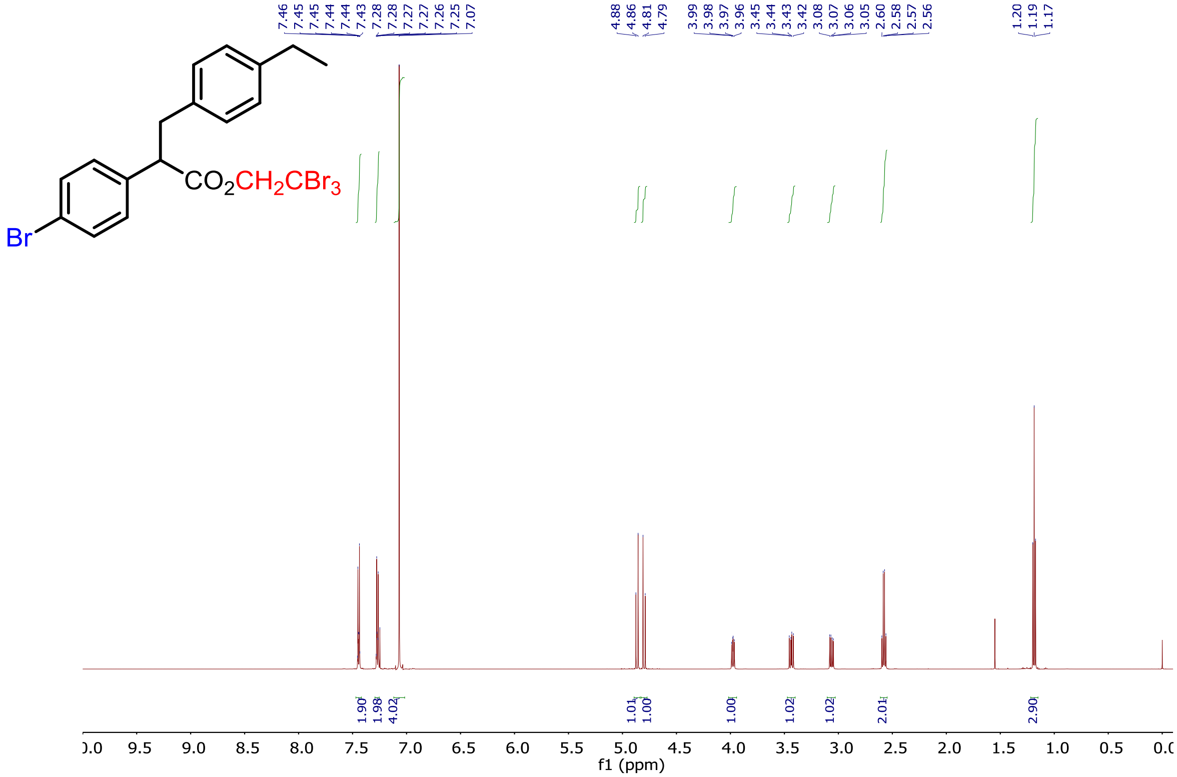
7.46 7.45 7.45 7.44 7.44 7.43 7.28 7.28 7.27 7.27 7.26 7.25 7.07
4.88 4.86 4.81 4.79
3.99 3.98 3.97 3.96 3.45 3.44 3.43 3.42 3.08 3.07 3.06 3.05 2.60 2.58 2.57 2.56
1.20 1.19 1.17
CO2CH2CBr3
Br
1.90 1.98 4.02 1.01 1.00 1.00 1.02 1.02 2.01 2.90
f1 (ppm)

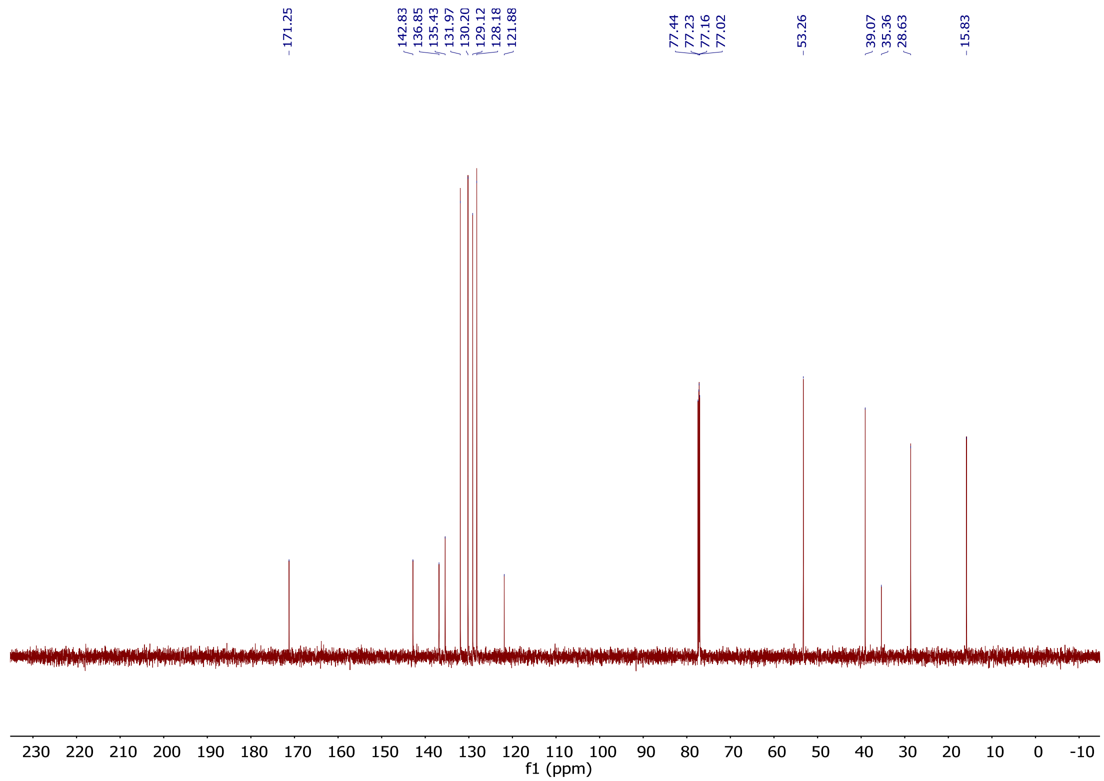
171.25
142.83 136.85 135.43 131.97 130.20 129.12 128.18 121.88
77.44 77.23 77.16 77.02
53.26
39.07 35.36 28.63
15.83
f1 (ppm)

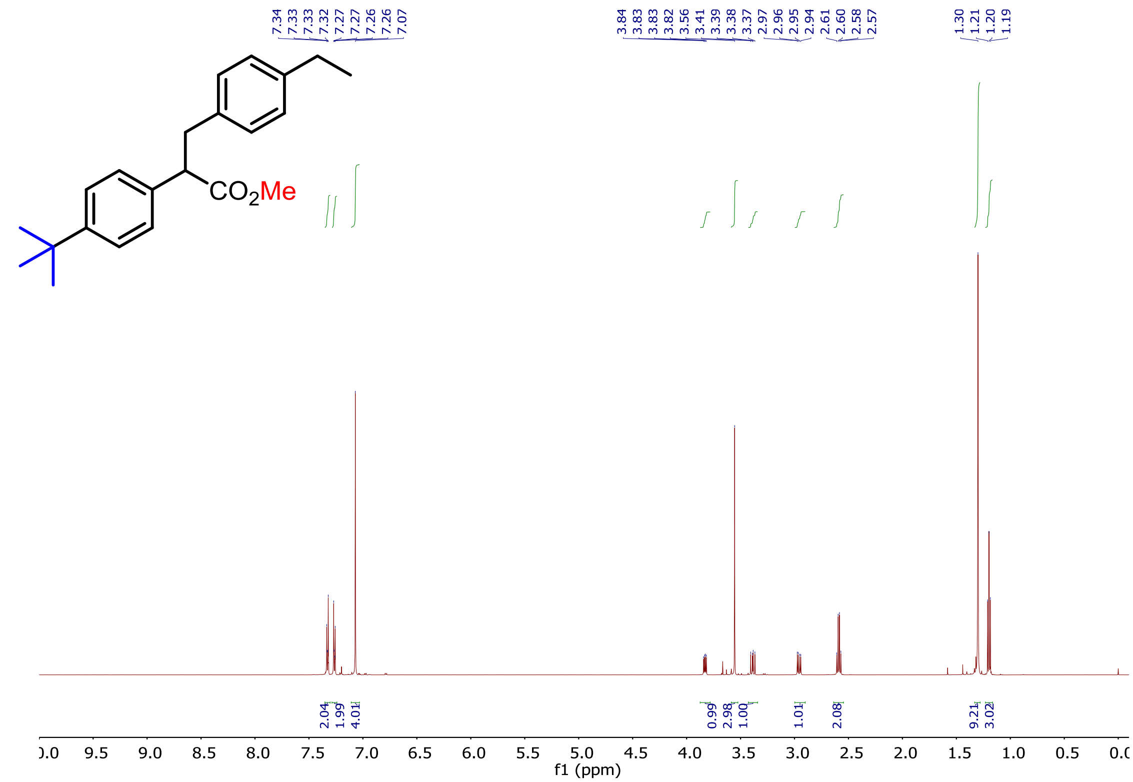

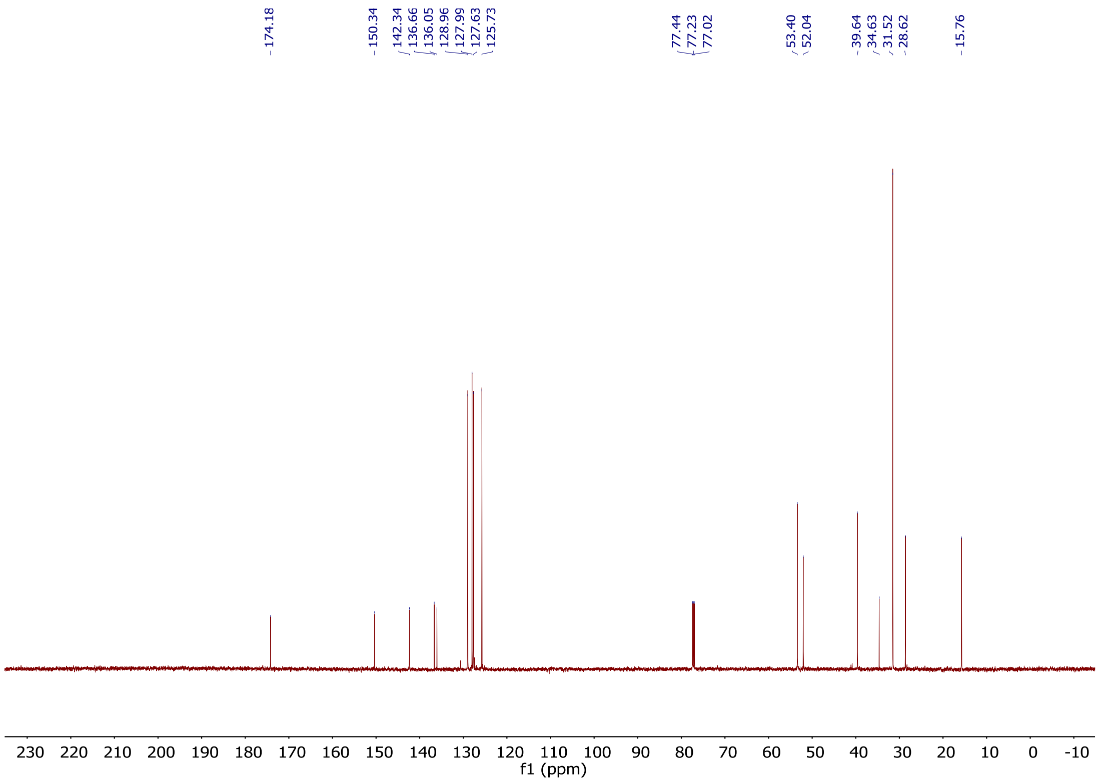

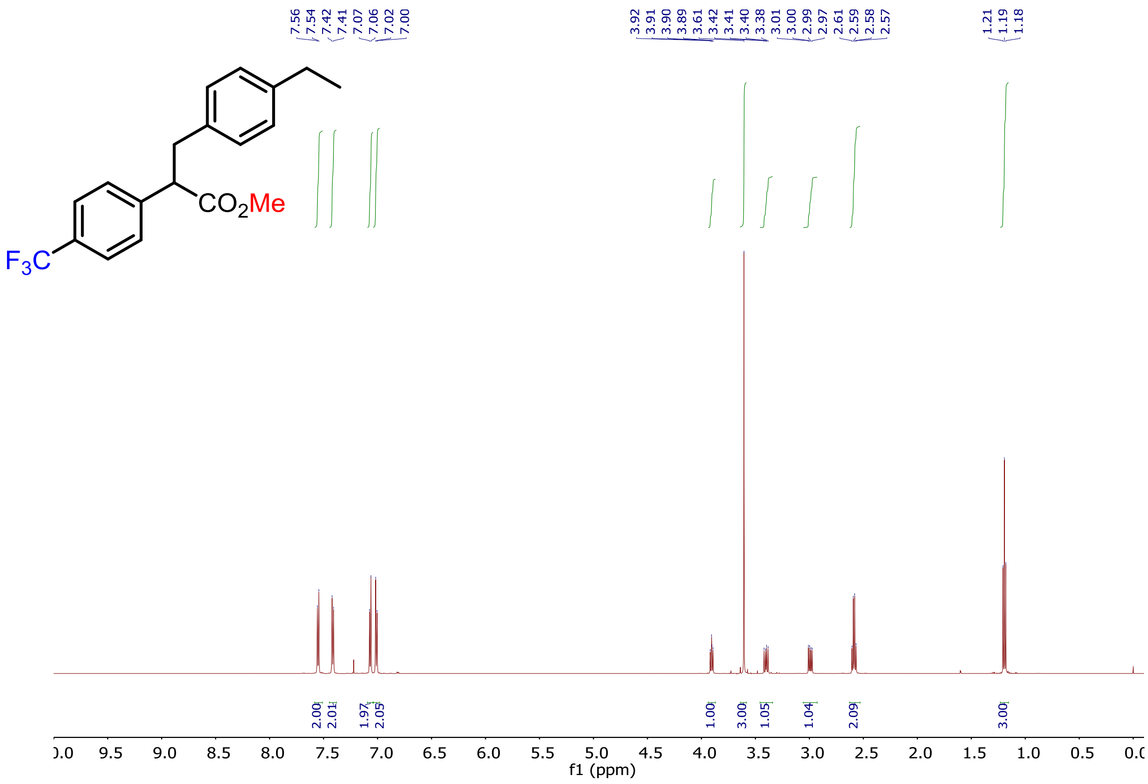

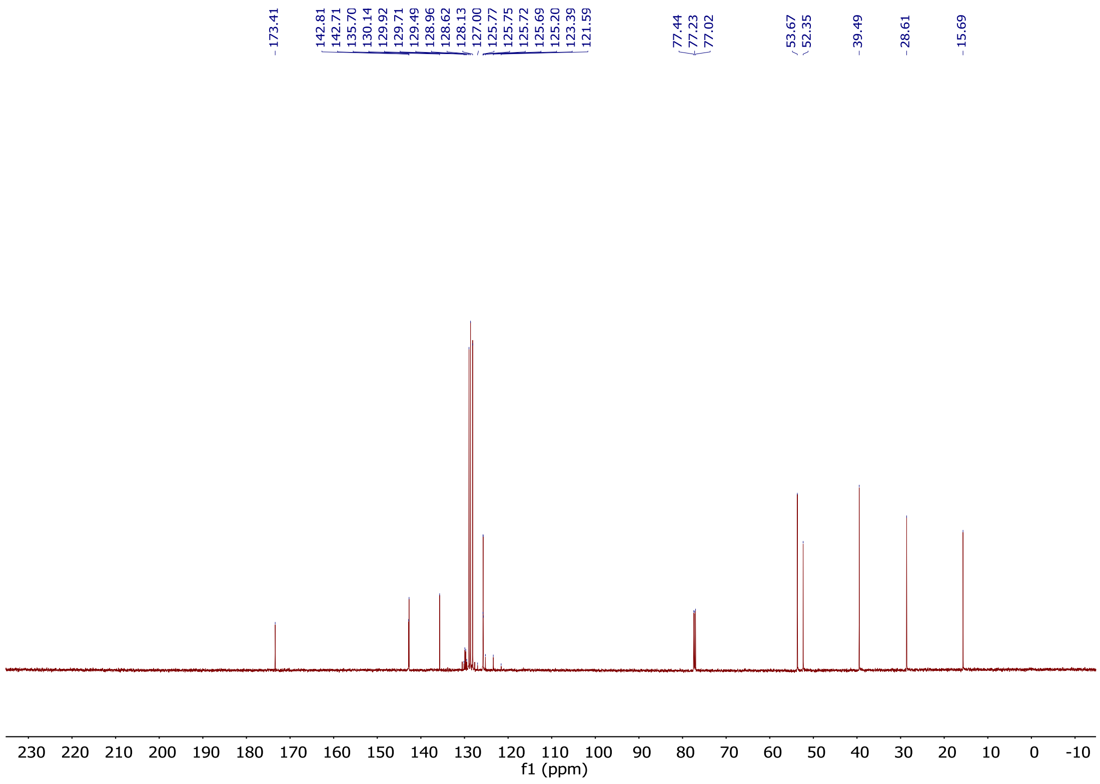

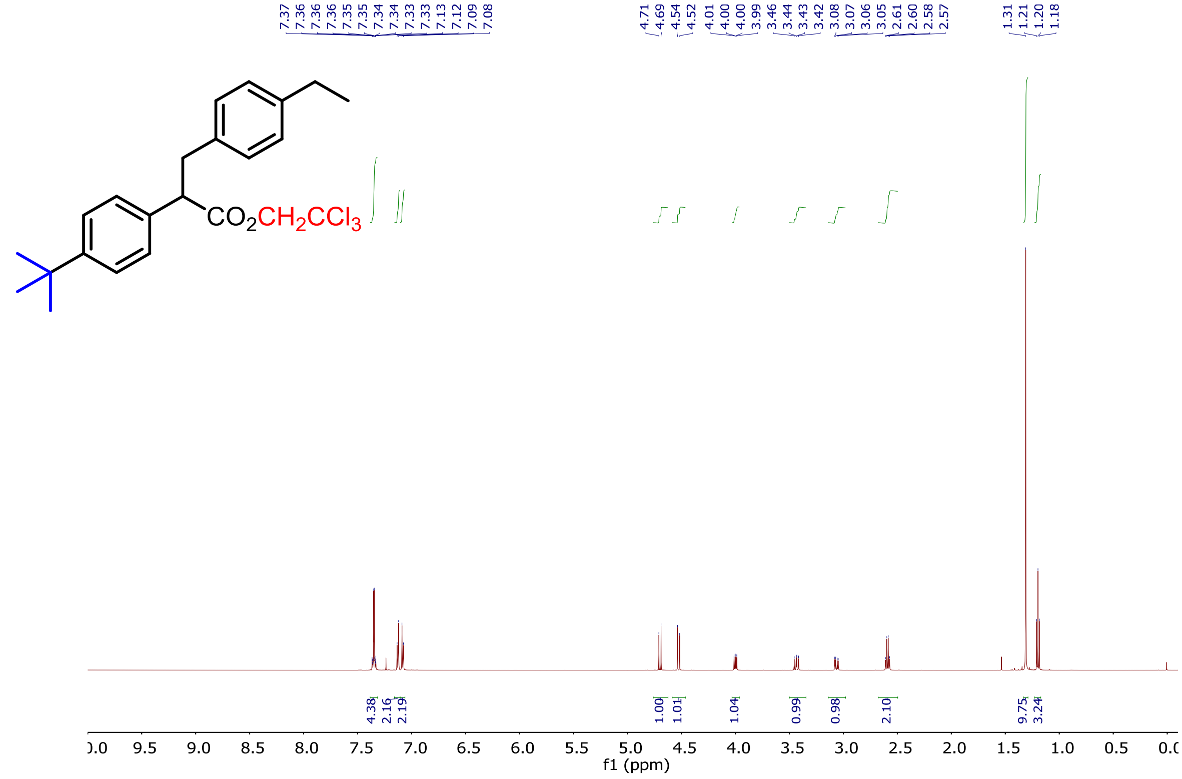

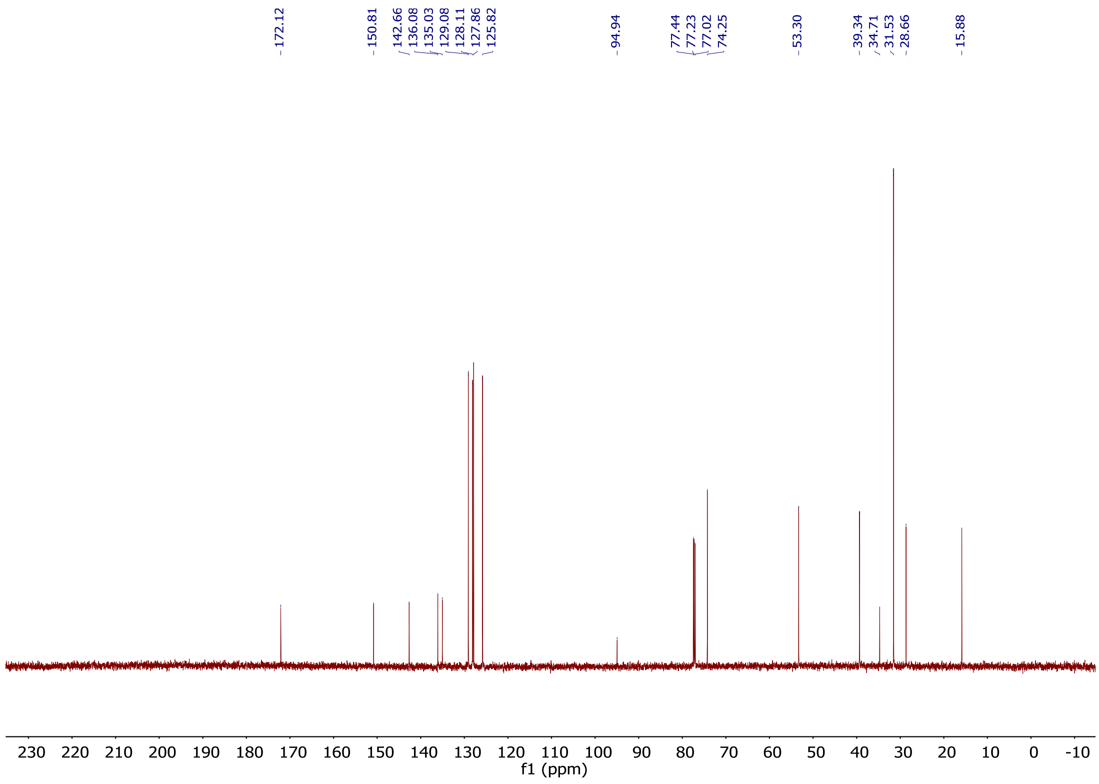

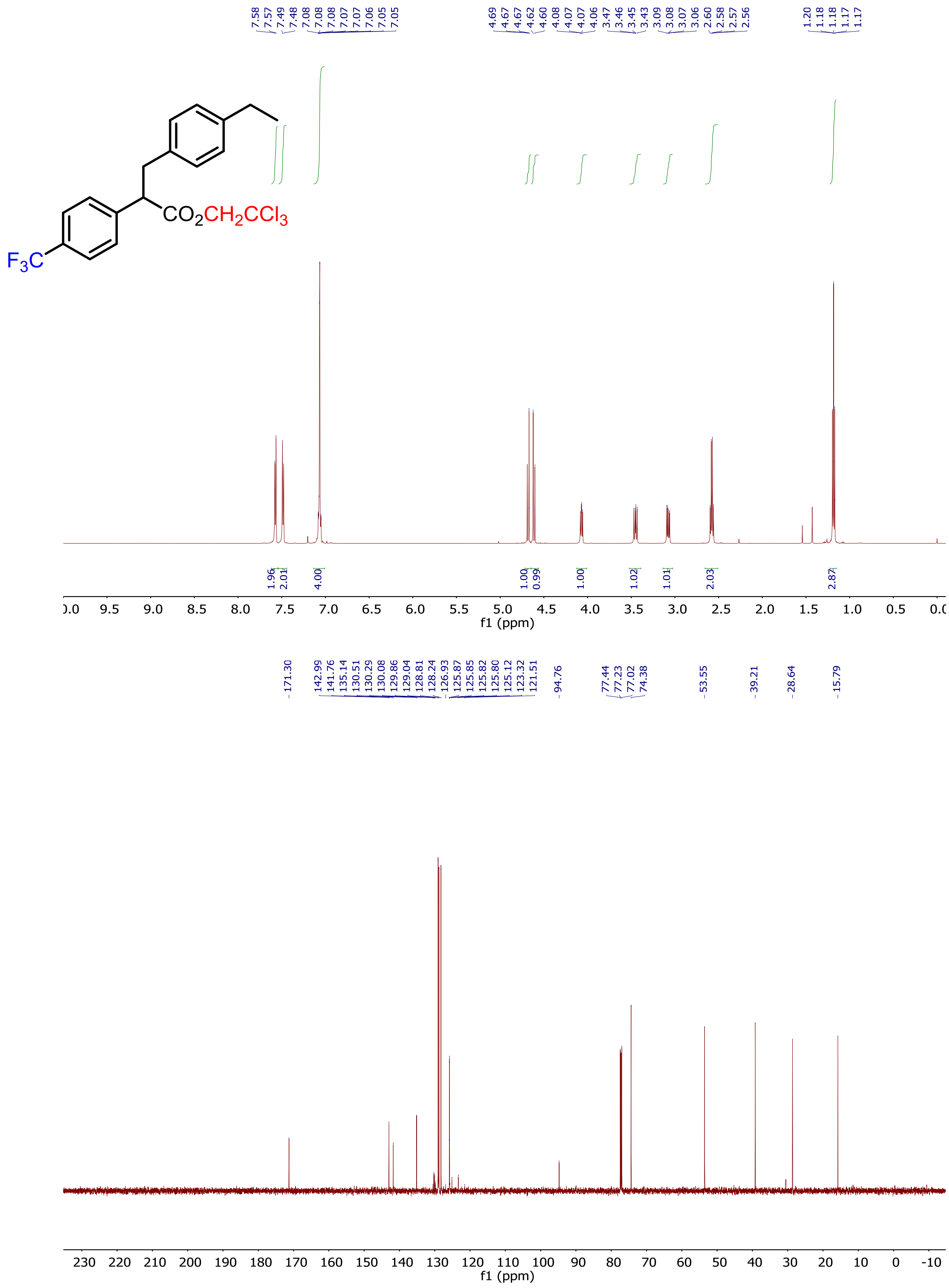
CO2CH2CCl3
F3C
7.58 7.57 7.49 7.48 7.08 7.08 7.08 7.07 7.07 7.06 7.05 7.05
4.69 4.67 4.67 4.62 4.60 4.08 4.07 4.07 4.06 3.47 3.46 3.45 3.43 3.09 3.08 3.07 3.06 2.60 2.58 2.57 2.56
1.20 1.18 1.18 1.17 1.17
1.96 2.01 4.00 1.00 0.99 1.00 1.02 1.01 2.03 2.87
f1 (ppm)
171.30
142.99 141.76 135.14 130.51 130.29 130.08 129.86 129.04 128.81 128.24 126.93 125.87 125.85 125.82 125.80 125.12 123.32 121.51
94.76
77.44 77.23 77.02 74.38
53.55
39.21
28.64
15.79
f1 (ppm)

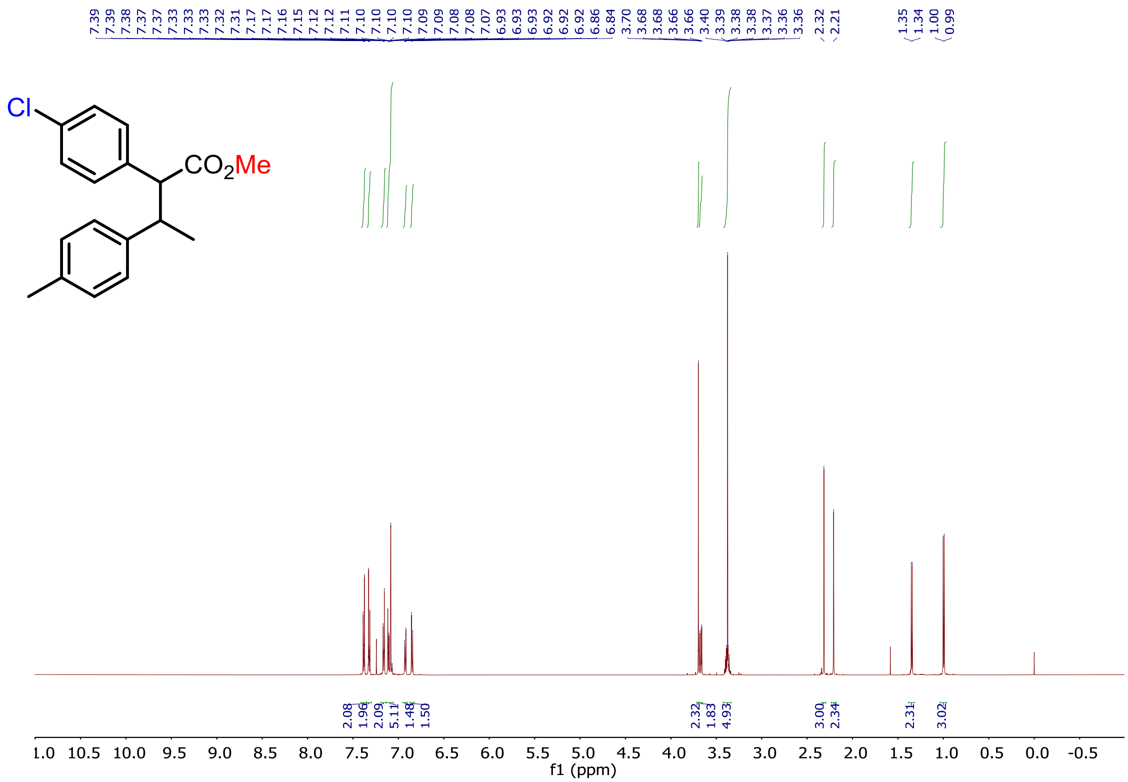
Cl
CO2Me
7.39 7.39 7.38 7.37 7.37 7.33 7.33 7.32 7.31 7.17 7.17 7.16 7.15 7.12 7.12 7.11 7.10 7.10 7.10 7.10 7.09 7.09 7.08 7.08 7.07 6.93 6.93 6.93 6.92 6.92 6.92 6.86 6.84 3.70 3.68 3.68 3.66 3.66 3.40 3.39 3.38 3.38 3.37 3.36 3.36 2.32 2.21 1.35 1.34 1.00 0.99
2.08 1.96 2.09 5.11 1.48 1.50 2.32 1.83 4.93 3.00 2.34 2.31 3.02
11.0 10.5 10.0 9.5 9.0 8.5 8.0 7.5 7.0 6.5 6.0 5.5 5.0 4.5 4.0 3.5 3.0 2.5 2.0 1.5 1.0 0.5 0.0 -0.5
f1 (ppm)

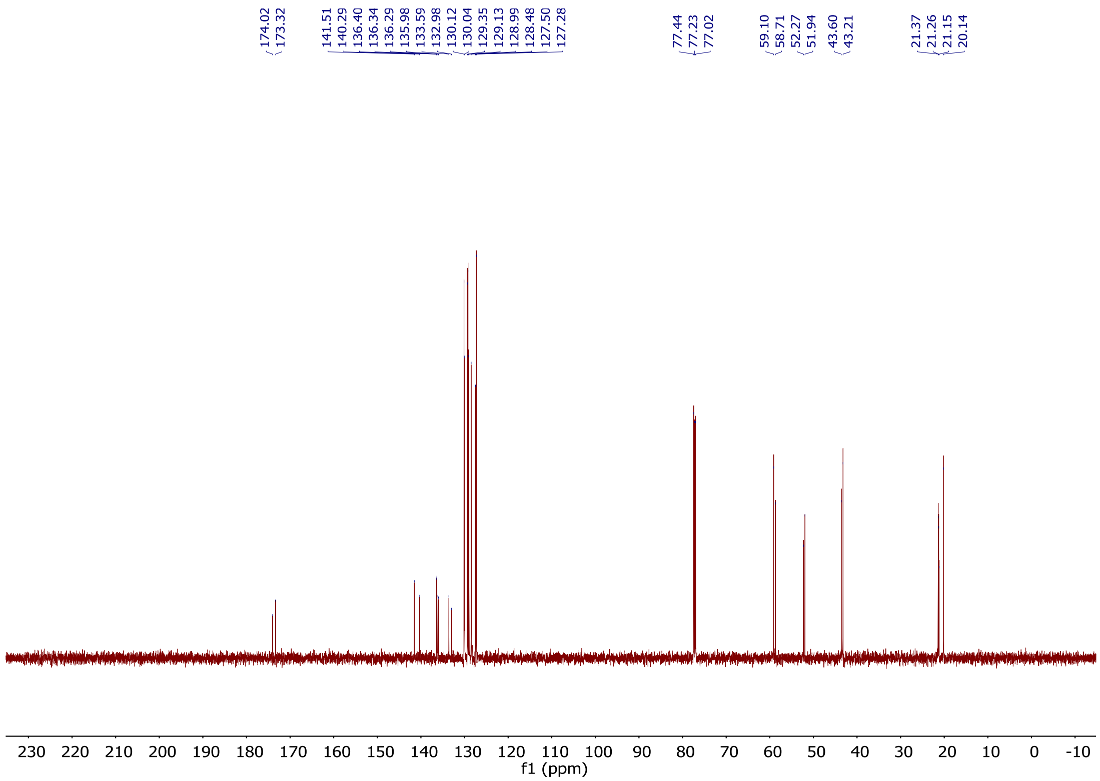
174.02 173.32 141.51 140.29 136.40 136.34 136.29 135.98 133.59 132.98 130.12 130.04 129.35 129.13 128.99 128.48 127.50 127.28 77.44 77.23 77.02 59.10 58.71 52.27 51.94 43.60 43.21 21.37 21.26 21.15 20.14
230 220 210 200 190 180 170 160 150 140 130 120 110 100 90 80 70 60 50 40 30 20 10 0 -10
f1 (ppm)

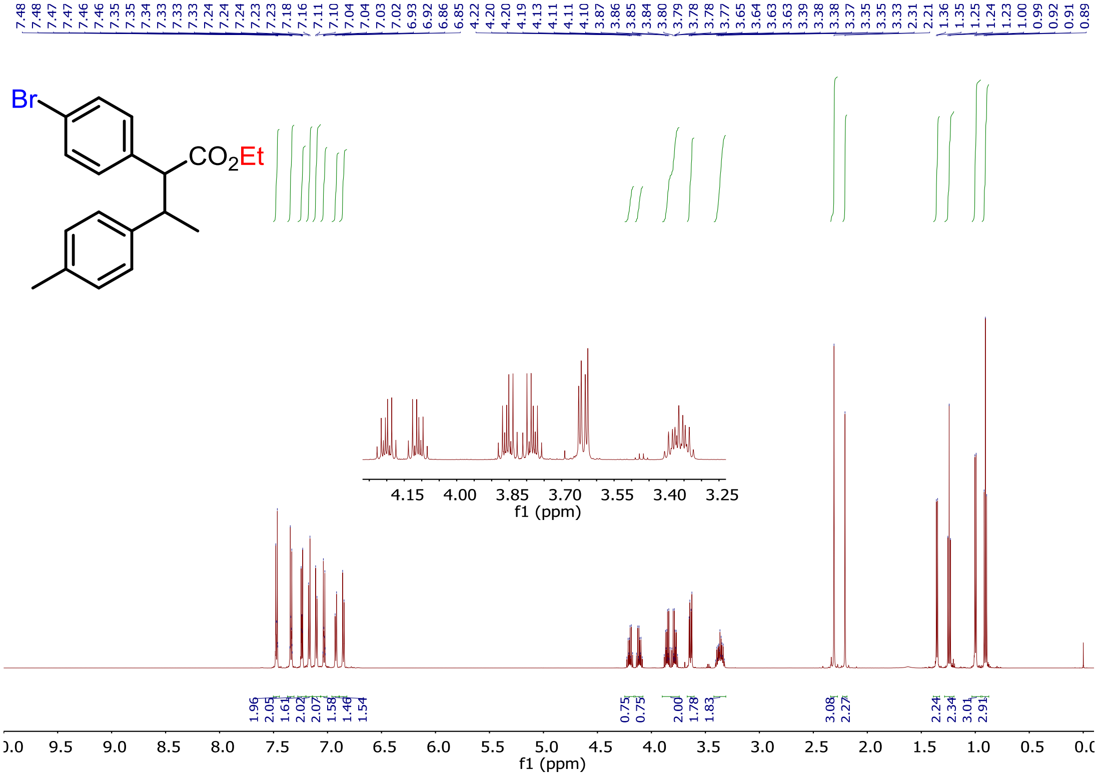
7.48 7.48 7.47 7.47 7.46 7.35 7.35 7.34 7.33 7.33 7.33 7.24 7.24 7.24 7.23 7.23 7.18 7.16 7.11 7.10 7.04 7.04 7.03 7.02 6.93 6.92 6.86 6.85 4.22 4.20 4.19 4.13 4.11 4.11 4.10 3.87 3.86 3.85 3.84 3.80 3.79 3.78 3.78 3.77 3.65 3.64 3.63 3.63 3.39 3.38 3.38 3.37 3.35 3.35 3.33 2.31 2.21 1.36 1.35 1.25 1.24 1.23 1.00 0.99 0.92 0.91 0.89
Br
CO_2Et
4.15 4.00 3.85 3.70 3.55 3.40 3.25
f1 (ppm)
1.96 2.05 1.61 2.02 2.07 1.58 1.46 1.54
0.75 0.75 2.00 1.78 1.83
3.08 2.27
2.24 2.34 3.01 2.91
10.0 9.5 9.0 8.5 8.0 7.5 7.0 6.5 6.0 5.5 5.0 4.5 4.0 3.5 3.0 2.5 2.0 1.5 1.0 0.5 0.0
f1 (ppm)

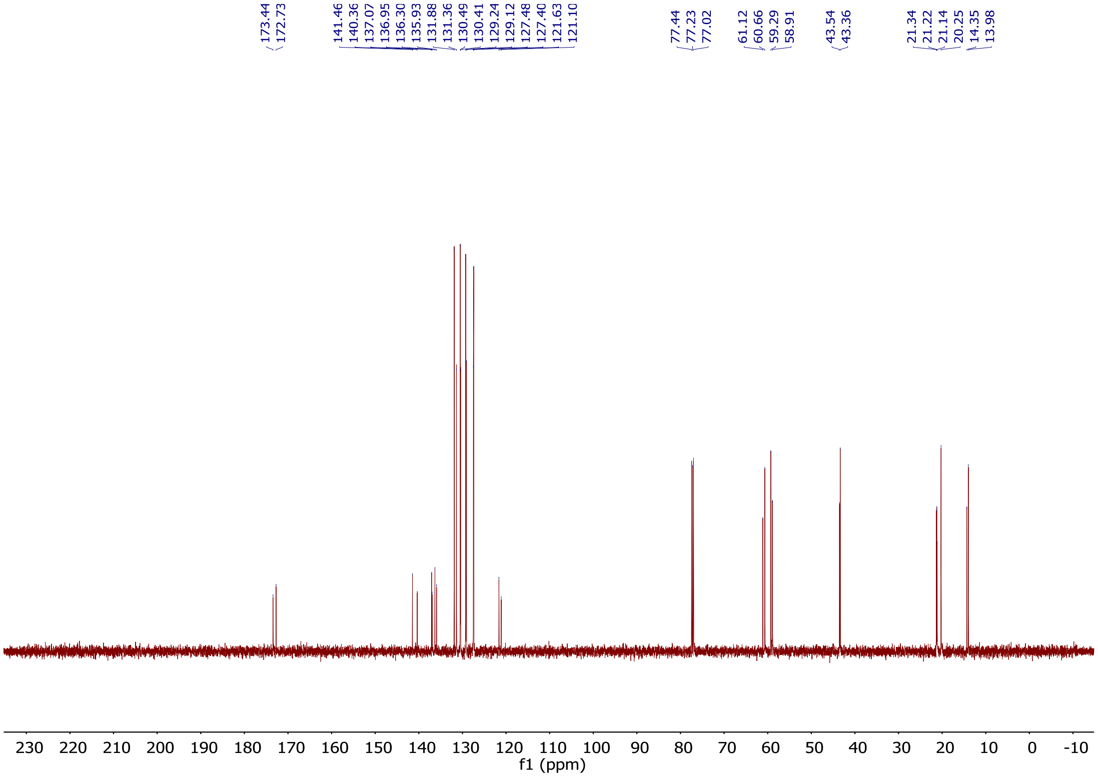
173.44 172.73
141.46 140.36 137.07 136.95 136.30 135.93 131.88 131.36 130.49 130.41 129.24 129.12 127.48 127.40 121.63 121.10
77.44 77.23 77.02
61.12 60.66 59.29 58.91
43.54 43.36
21.34 21.22 21.14 20.25 14.35 13.98
230 220 210 200 190 180 170 160 150 140 130 120 110 100 90 80 70 60 50 40 30 20 10 0 -10
f1 (ppm)

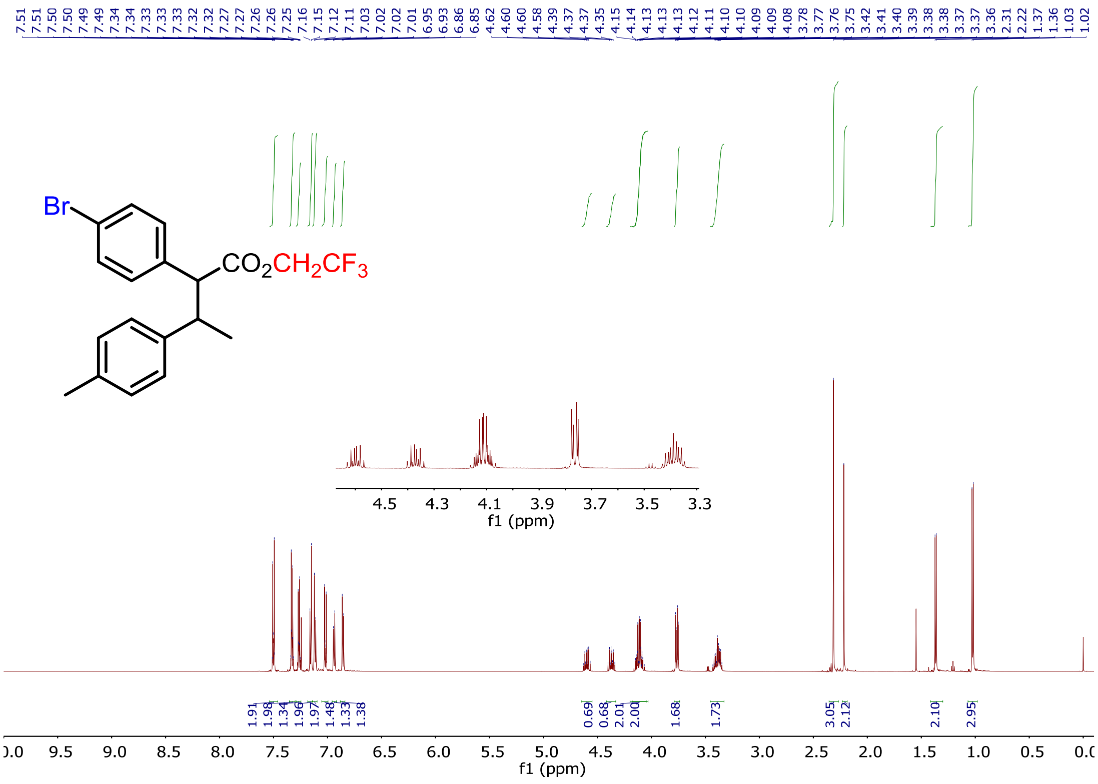
7.51 7.51 7.50 7.50 7.49 7.49 7.34 7.34 7.33 7.33 7.33 7.32 7.32 7.27 7.27 7.26 7.26 7.25 7.16 7.15 7.12 7.11 7.03 7.02 7.02 7.01 6.95 6.93 6.86 6.85 4.62 4.60 4.60 4.58 4.39 4.37 4.37 4.35 4.15 4.14 4.13 4.13 4.13 4.12 4.11 4.10 4.10 4.09 4.09 4.08 3.78 3.77 3.76 3.75 3.42 3.41 3.40 3.39 3.38 3.38 3.37 3.37 3.36 2.31 2.22 1.37 1.36 1.03 1.02
Br
$CO_2CH_2CF_3$
4.5 4.3 4.1 3.9 3.7 3.5 3.3
f1 (ppm)
1.91 1.98 1.34 1.96 1.97 1.48 1.33 1.38
0.69 0.68 2.01 2.00 1.68 1.73 3.05 2.12 2.10 2.95
10.0 9.5 9.0 8.5 8.0 7.5 7.0 6.5 6.0 5.5 5.0 4.5 4.0 3.5 3.0 2.5 2.0 1.5 1.0 0.5 0.0
f1 (ppm)

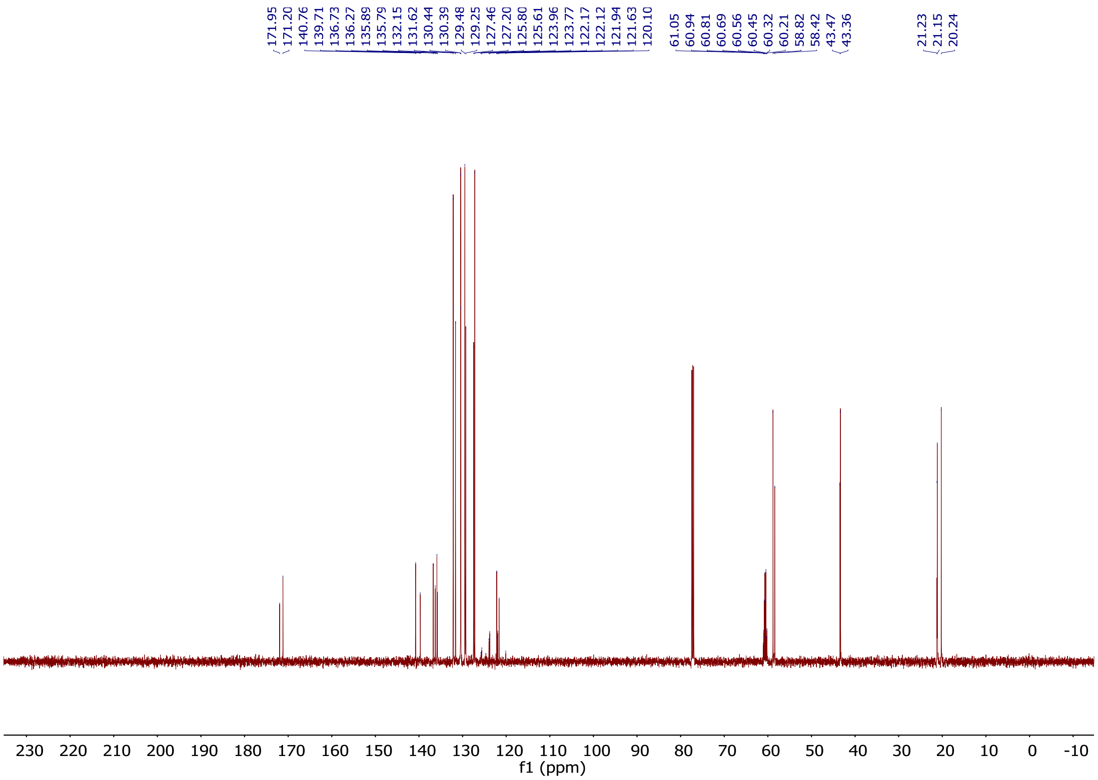
171.95 171.20 140.76 139.71 136.73 136.27 135.89 135.79 132.15 131.62 130.44 130.39 129.48 129.25 127.46 127.20 125.80 125.61 123.96 123.77 122.17 122.12 121.94 121.63 120.10
61.05 60.94 60.81 60.69 60.56 60.45 60.32 60.21 58.82 58.42 43.47 43.36
21.23 21.15 20.24
230 220 210 200 190 180 170 160 150 140 130 120 110 100 90 80 70 60 50 40 30 20 10 0 -10
f1 (ppm)

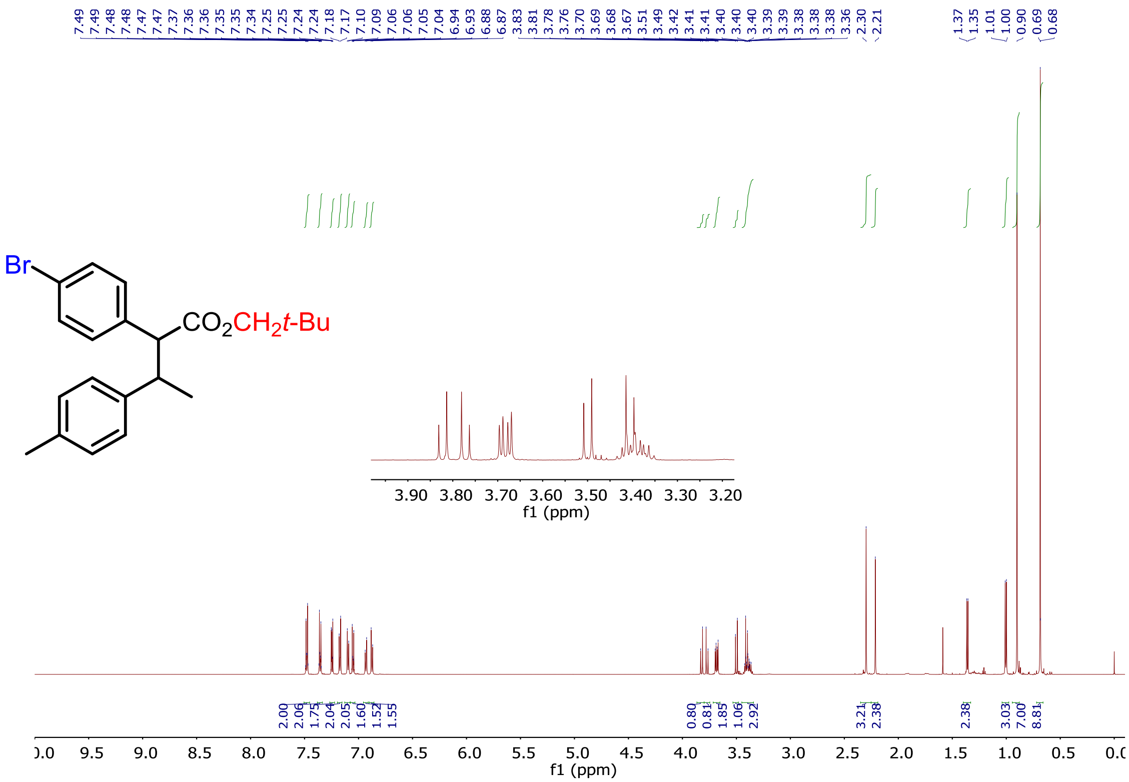

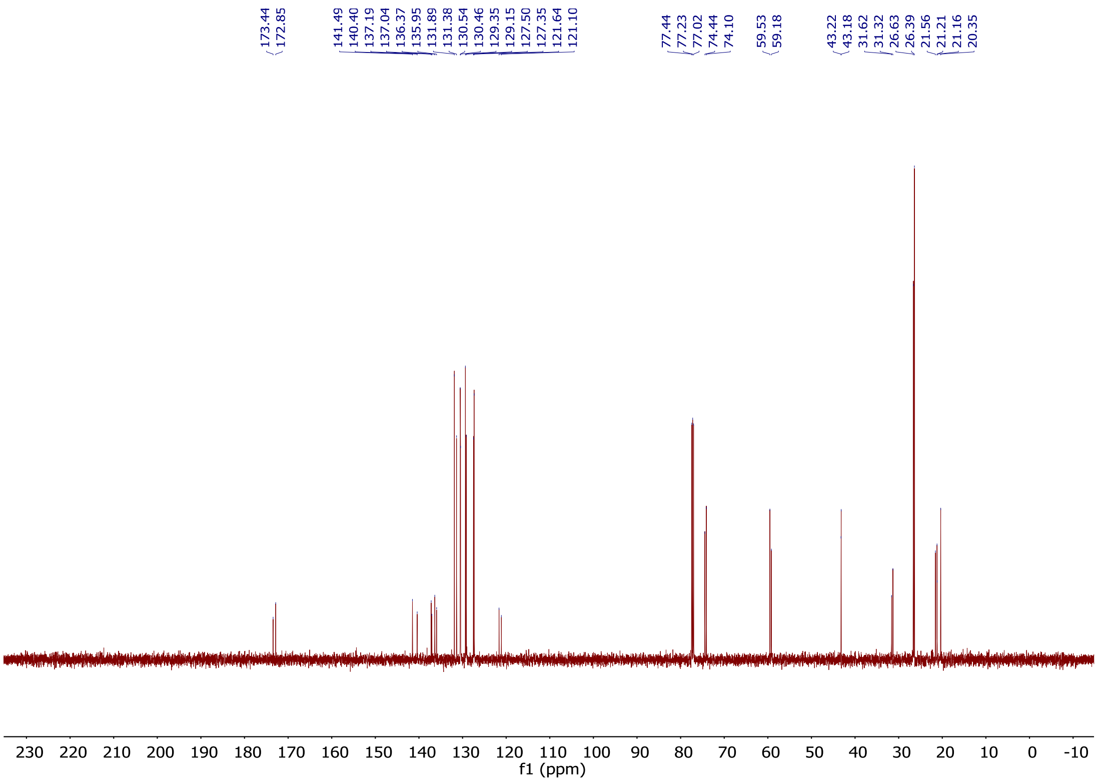

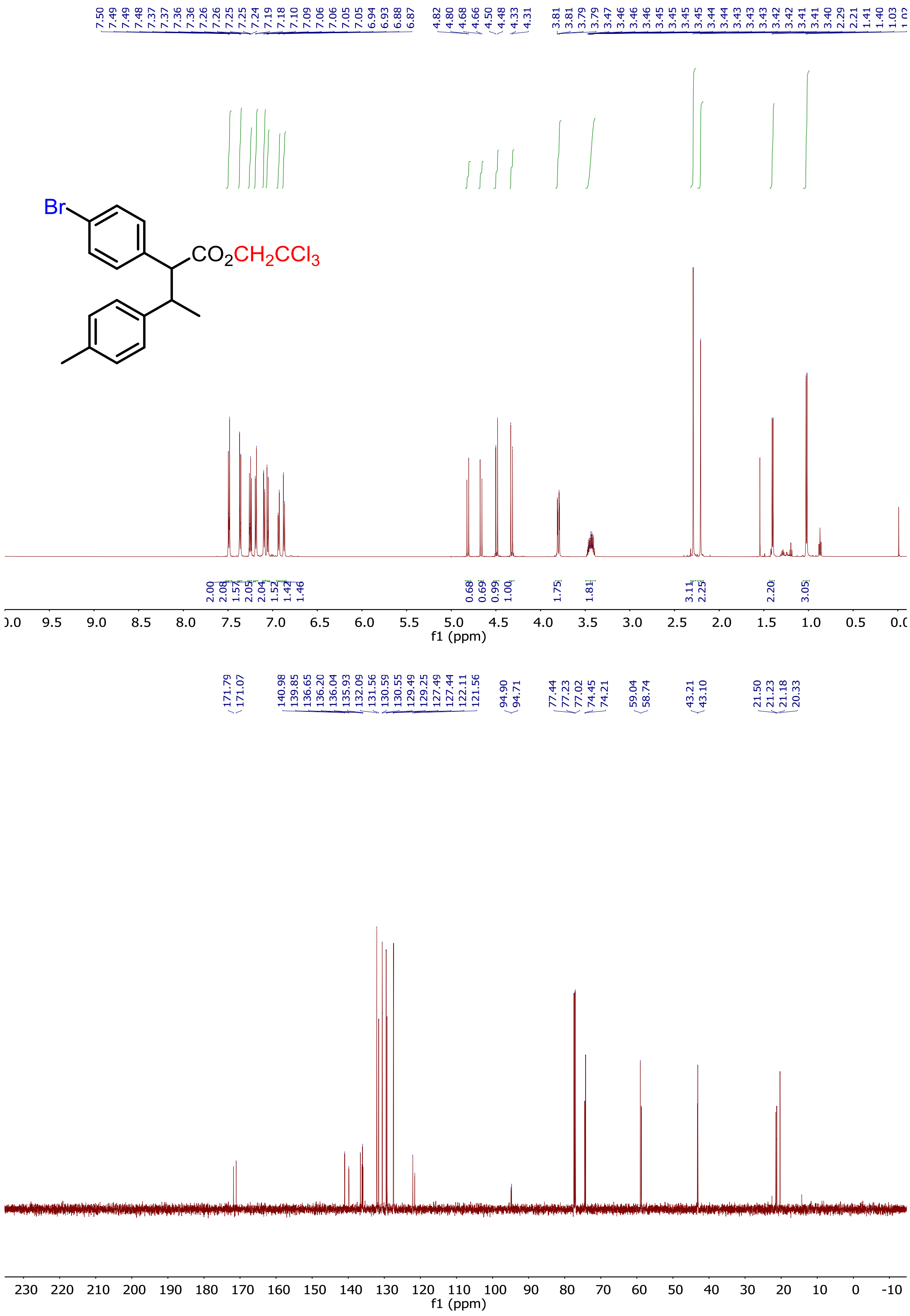
Br
$CO_2CH_2CCl_3$
f1 (ppm)
f1 (ppm)

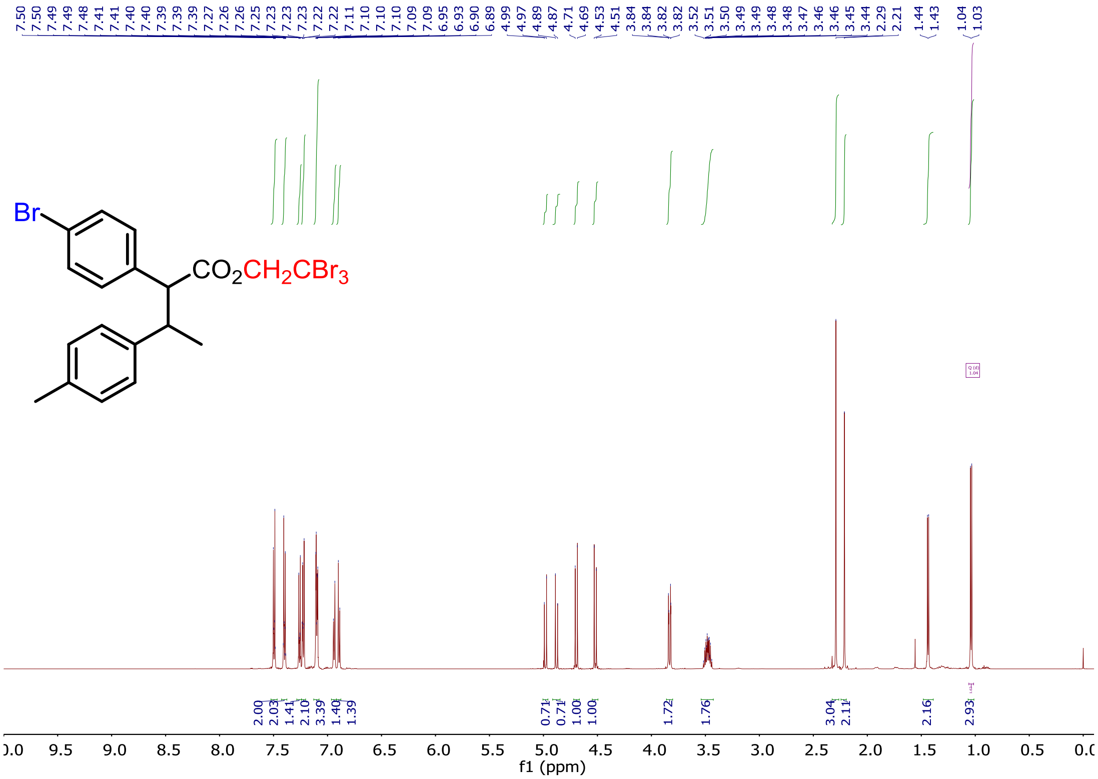
7.50 7.50 7.49 7.48 7.41 7.41 7.40 7.40 7.39 7.39 7.39 7.27 7.26 7.26 7.25 7.23 7.23 7.23 7.22 7.22 7.11 7.10 7.10 7.10 7.09 7.09 6.95 6.93 6.90 6.89 4.99 4.97 4.89 4.87 4.71 4.69 4.53 4.51 3.84 3.84 3.82 3.82 3.52 3.51 3.50 3.49 3.49 3.48 3.48 3.47 3.46 3.46 3.45 3.44 2.29 2.21 1.44 1.43 1.04 1.03
Br
$CO_2CH_2CBr_3$
2.00 2.03 1.41 2.10 3.39 1.40 1.39 0.71 0.71 1.00 1.00 1.72 1.76 3.04 2.11 2.16 2.93
f1 (ppm)

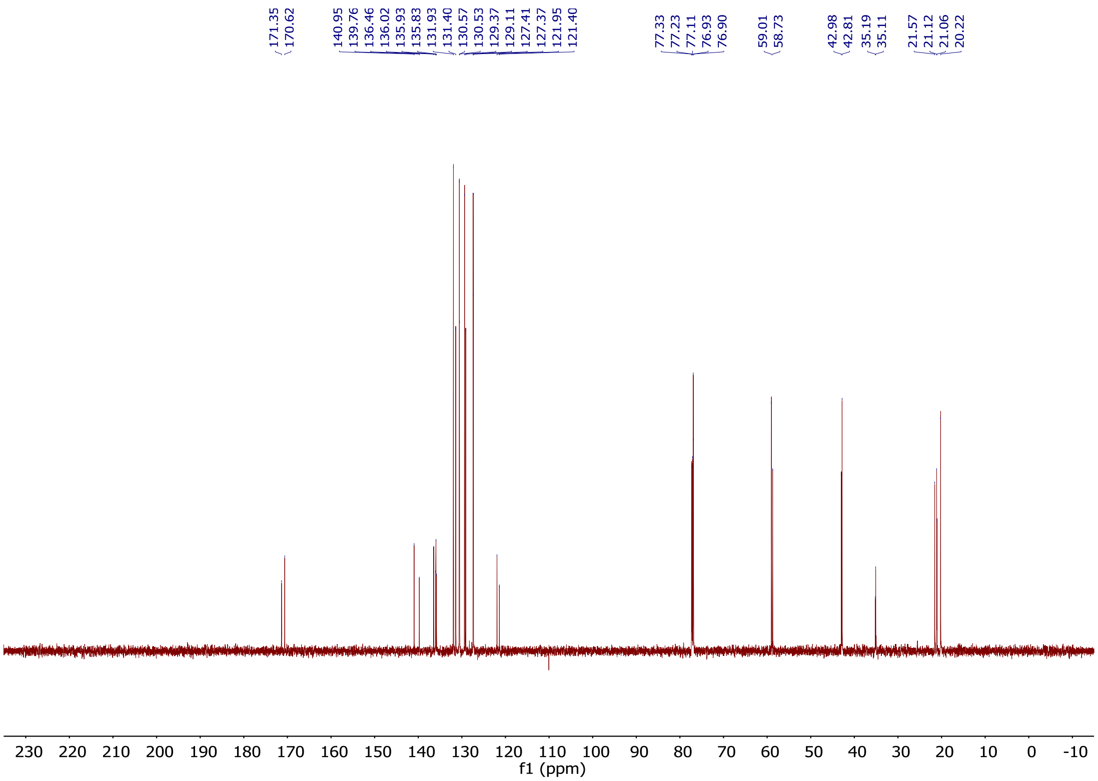
171.35 170.62 140.95 139.76 136.46 136.02 135.93 135.83 131.93 131.40 130.57 130.53 129.37 129.11 127.41 127.37 121.95 121.40 77.33 77.23 77.11 76.93 76.90 59.01 58.73 42.98 42.81 35.19 35.11 21.57 21.12 21.06 20.22
f1 (ppm)

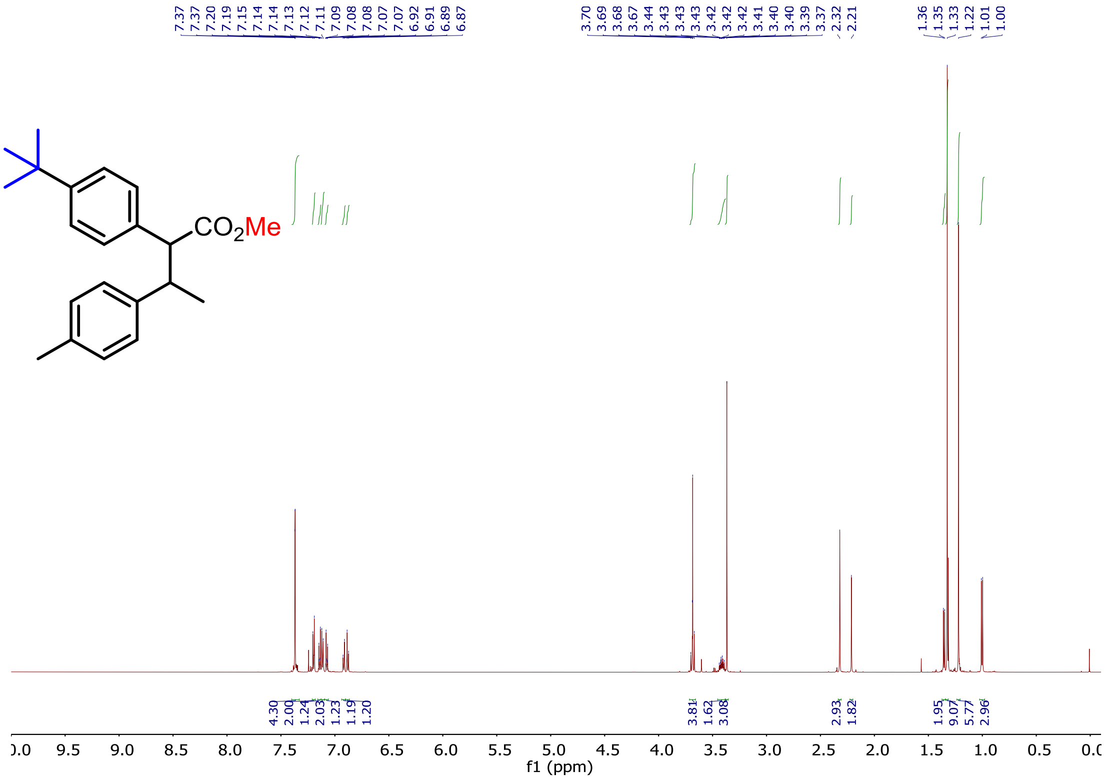

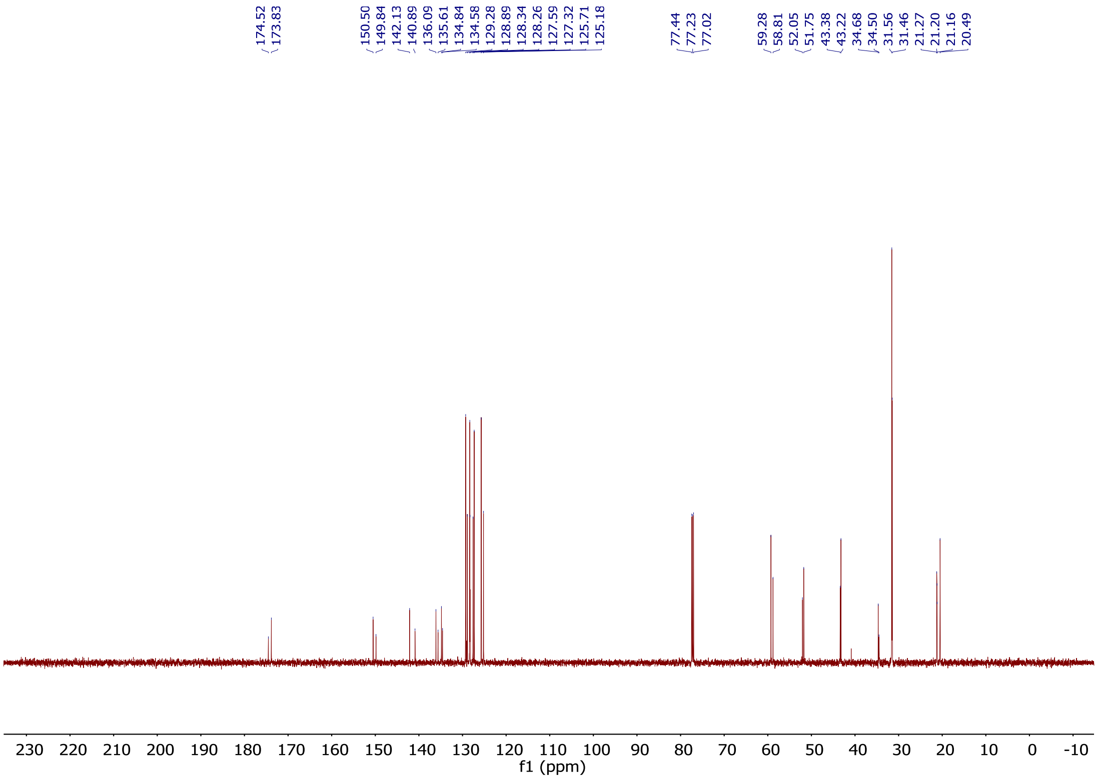

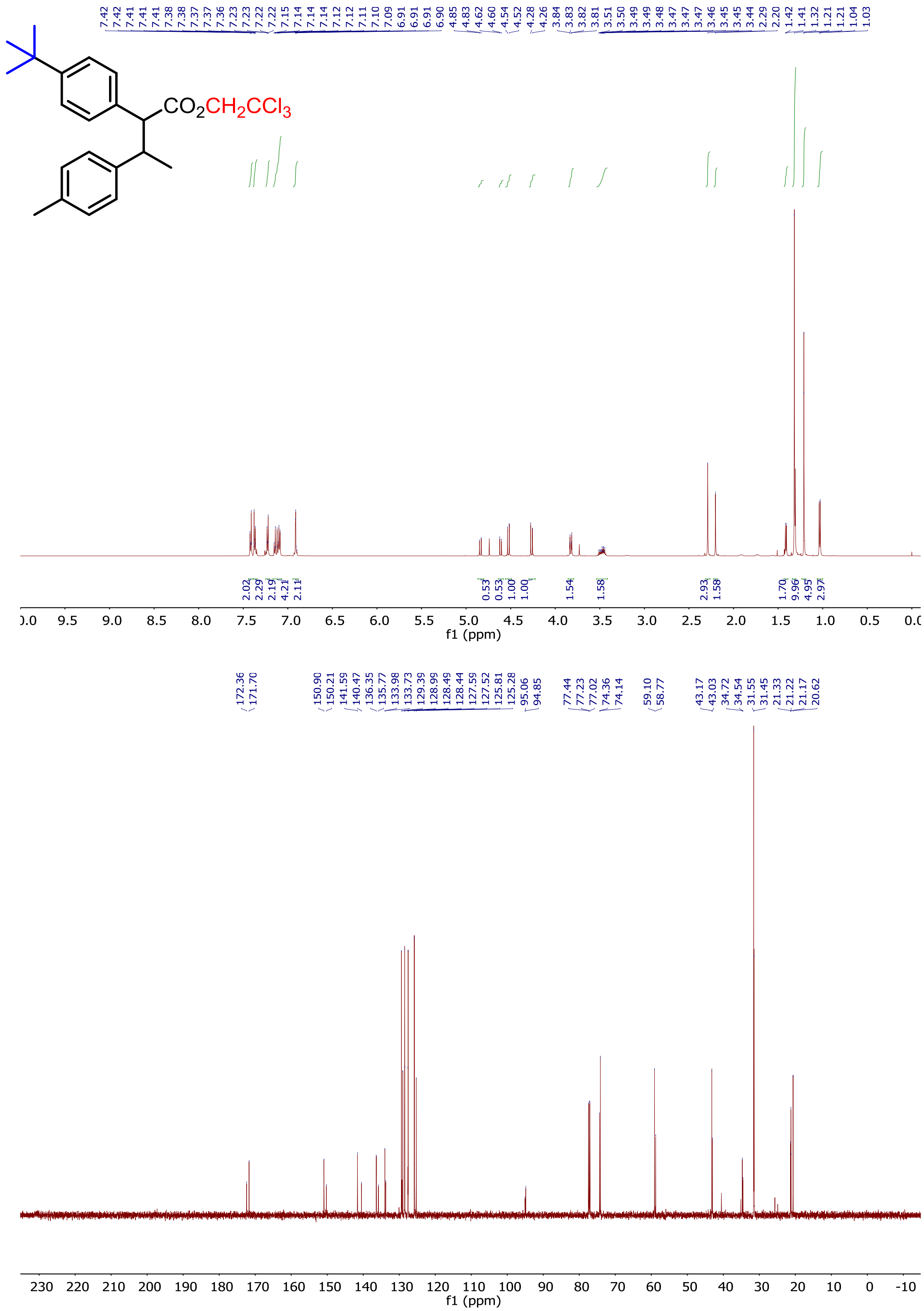
CO2CH2CCl3
7.42 7.42 7.41 7.41 7.41 7.38 7.38 7.37 7.37 7.36 7.23 7.22 7.22 7.15 7.14 7.14 7.14 7.12 7.12 7.11 7.10 7.09 6.91 6.91 6.91 6.90 4.85 4.83 4.62 4.60 4.54 4.52 4.28 4.26 3.84 3.83 3.82 3.81 3.51 3.50 3.49 3.49 3.48 3.47 3.47 3.46 3.45 3.45 3.44 2.29 2.20 1.42 1.41 1.32 1.21 1.21 1.04 1.03
2.02 2.29 2.19 4.21 2.11 0.53 0.53 1.00 1.00 1.54 1.58 2.93 1.58 1.70 9.96 4.95 2.97
f1 (ppm)
172.36 171.70 150.90 150.21 141.59 140.47 136.35 135.77 133.98 133.73 129.39 128.99 128.49 128.44 127.59 127.52 125.81 125.28 95.06 94.85 77.44 77.23 77.02 74.36 74.14 59.10 58.77 43.17 43.03 34.72 34.54 31.55 31.45 21.33 21.22 21.17 20.62
f1 (ppm)

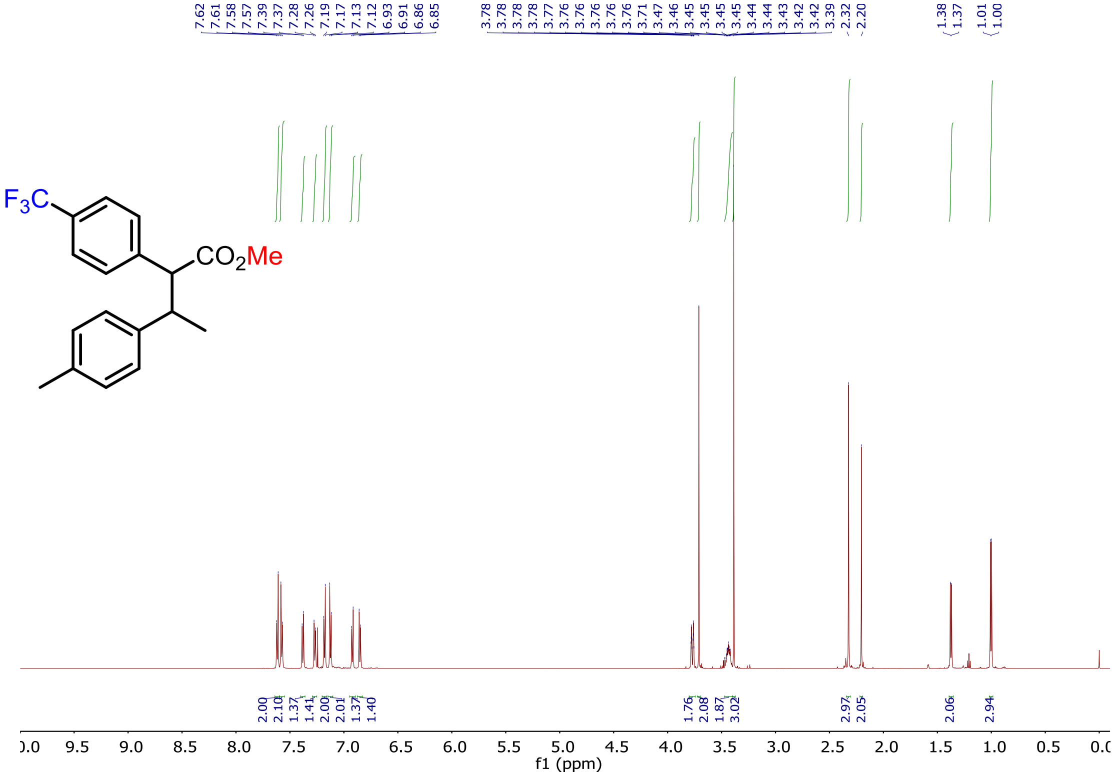
F3C
CO2Me

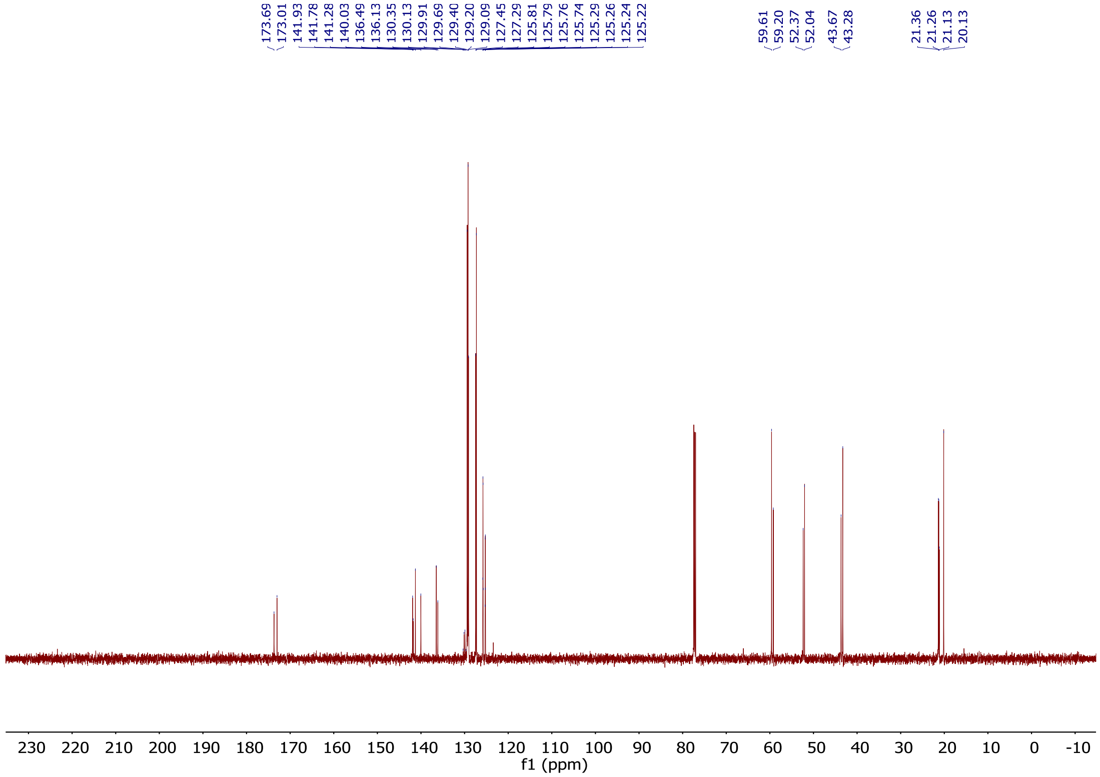

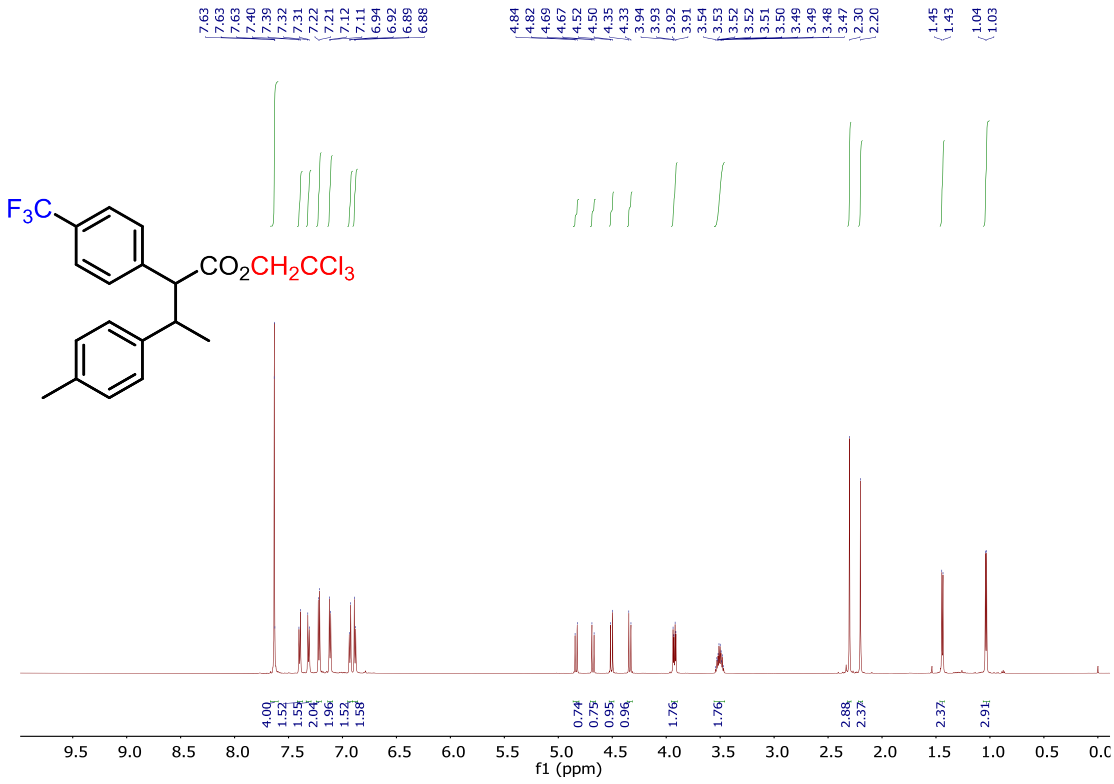
F3C
CO2CH2CCl3
7.63 7.63 7.63 7.40 7.39 7.32 7.31 7.22 7.21 7.12 7.11 6.94 6.92 6.89 6.88
4.84 4.82 4.69 4.67 4.52 4.50 4.35 4.33 3.94 3.93 3.92 3.91 3.54 3.53 3.52 3.52 3.51 3.50 3.49 3.49 3.48 3.47 2.30 2.20
1.45 1.43 1.04 1.03
4.00 1.52 1.55 2.04 1.96 1.52 1.58
0.74 0.75 0.95 0.96 1.76 1.76 2.88 2.37 2.37 2.91
9.5 9.0 8.5 8.0 7.5 7.0 6.5 6.0 5.5 5.0 4.5 4.0 3.5 3.0 2.5 2.0 1.5 1.0 0.5 0.0
f1 (ppm)

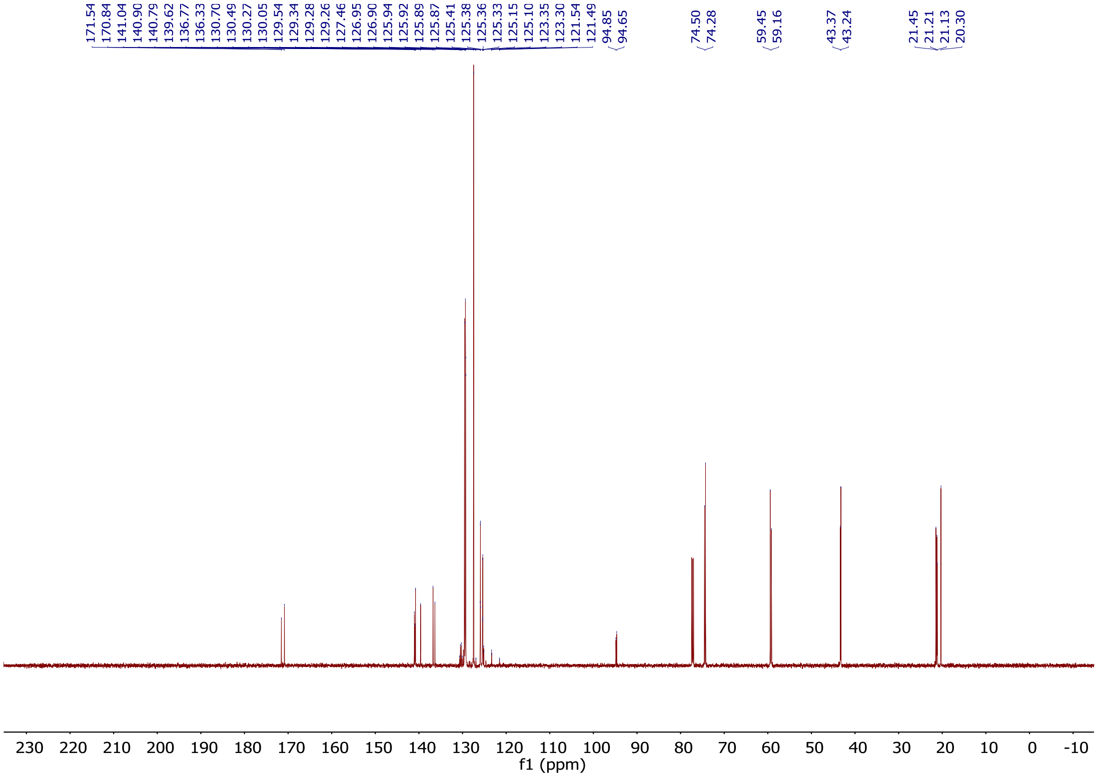
171.54 170.84 141.04 140.90 140.79 139.62 136.77 136.33 130.70 130.49 130.27 130.05 129.54 129.34 129.28 129.26 127.46 126.95 126.90 125.94 125.92 125.89 125.87 125.41 125.38 125.36 125.33 125.15 125.10 123.35 123.30 121.54 121.49 94.85 94.65
74.50 74.28 59.45 59.16 43.37 43.24 21.45 21.21 21.13 20.30
230 220 210 200 190 180 170 160 150 140 130 120 110 100 90 80 70 60 50 40 30 20 10 0 -10
f1 (ppm)

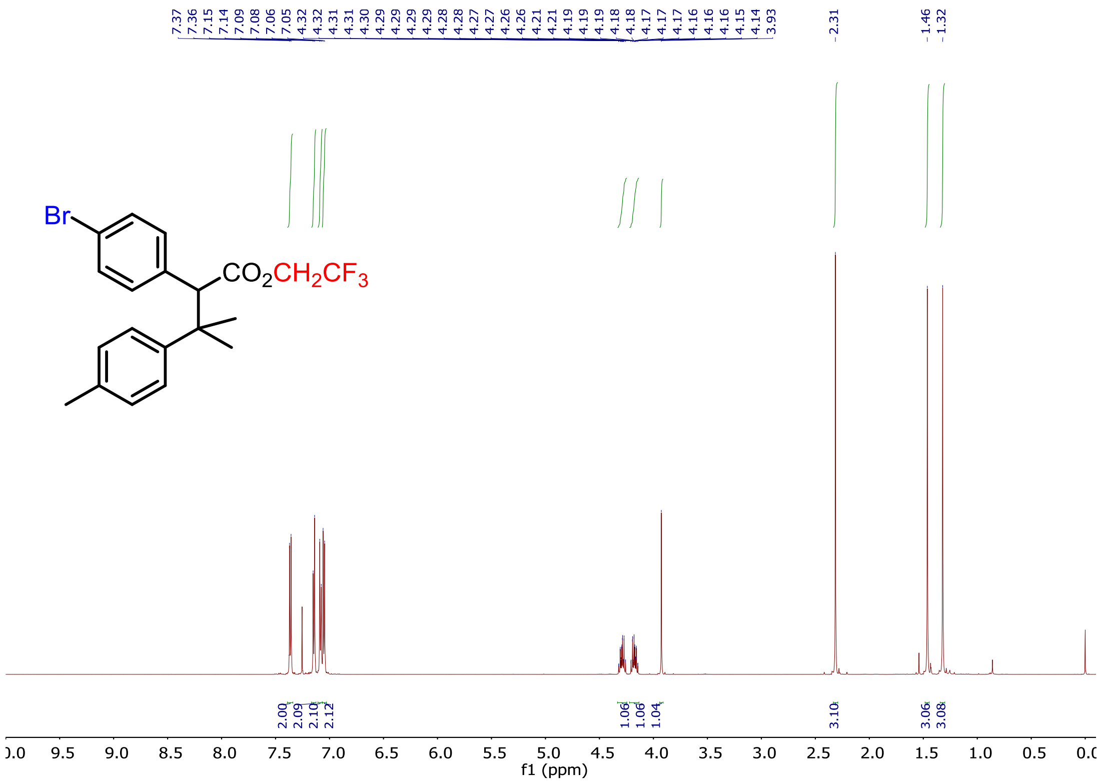

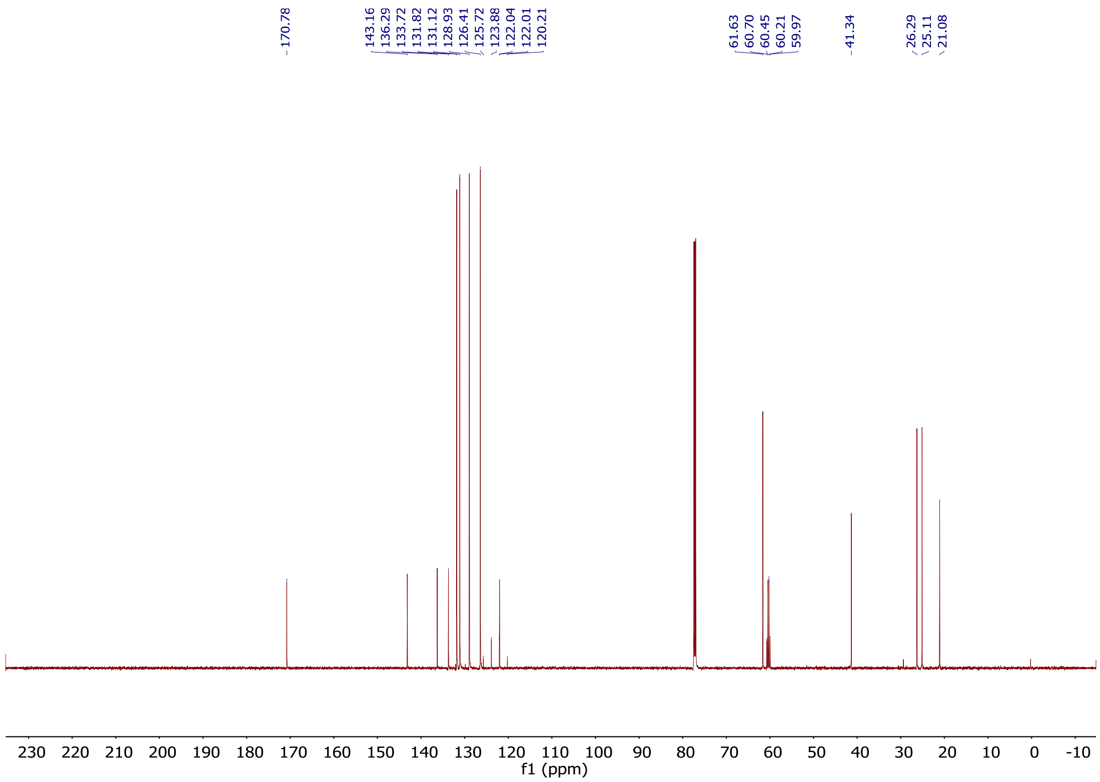

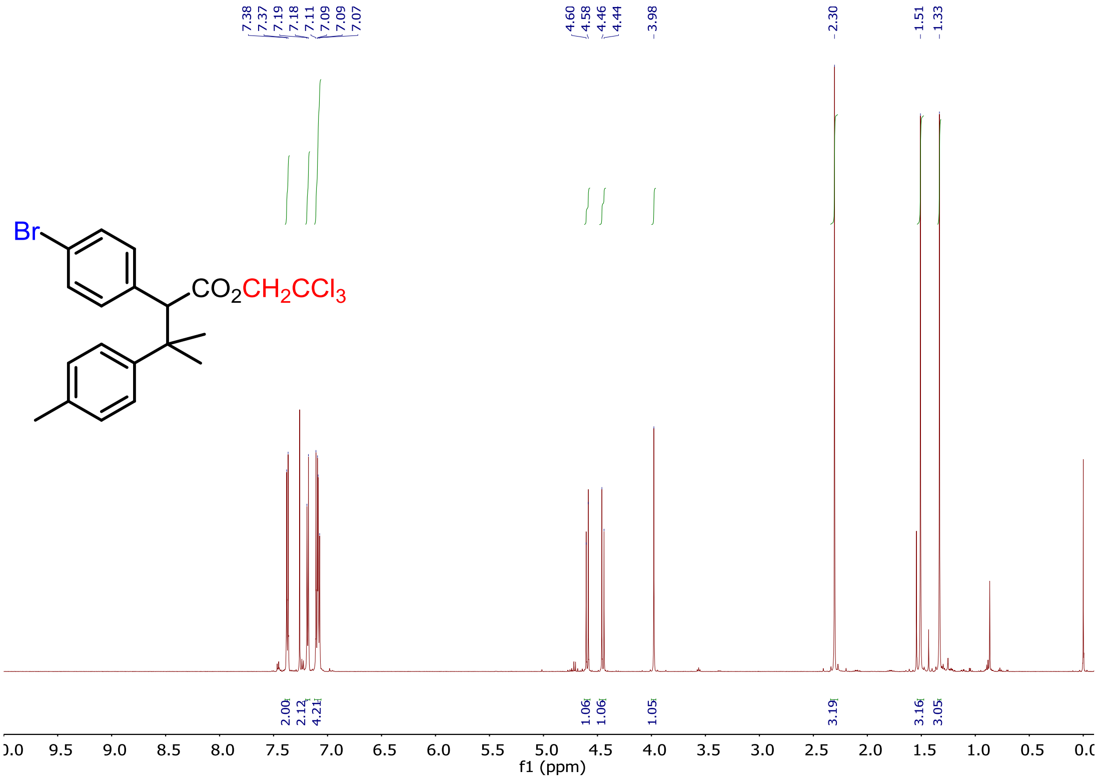
Br
$CO_2CH_2CCl_3$
7.38 7.37 7.19 7.18 7.11 7.09 7.09 7.07
4.60 4.58 4.46 4.44
3.98
2.30
1.51
1.33
2.00 2.12 4.21
1.06 1.06
1.05
3.19
3.16 3.05
f1 (ppm)

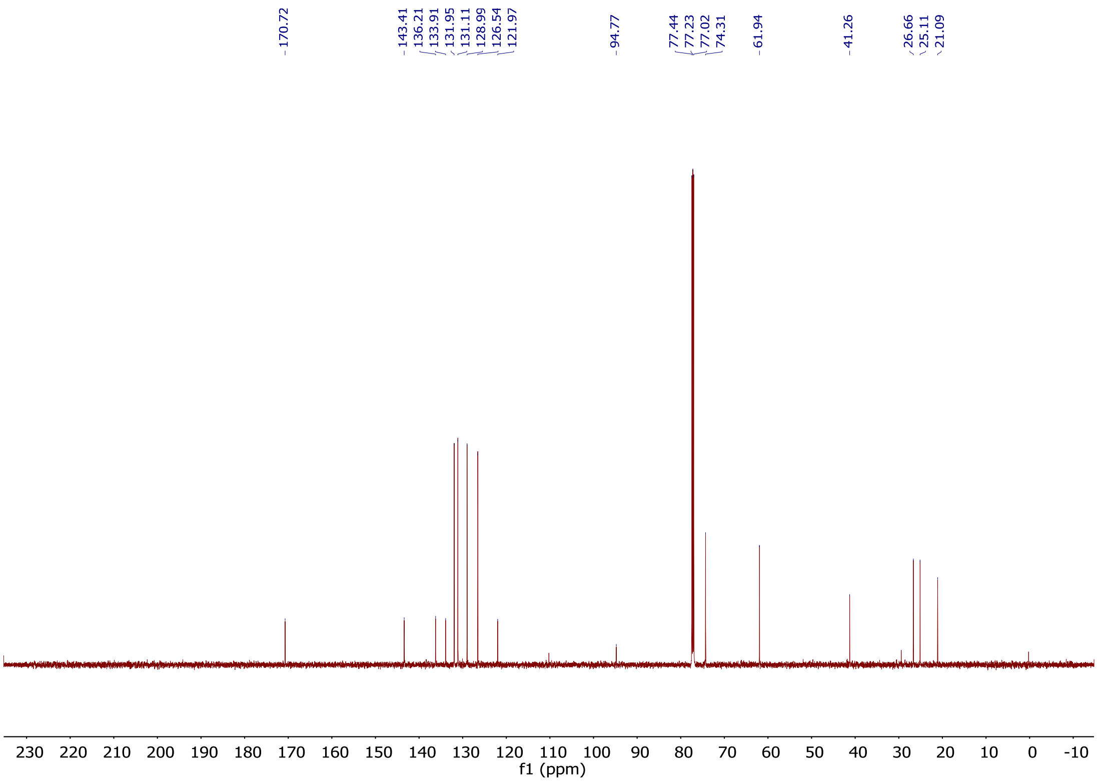
170.72
143.41 136.21 133.91 131.95 131.11 128.99 126.54 121.97
94.77
77.44 77.23 77.02 74.31
61.94
41.26
26.66 25.11 21.09
f1 (ppm)

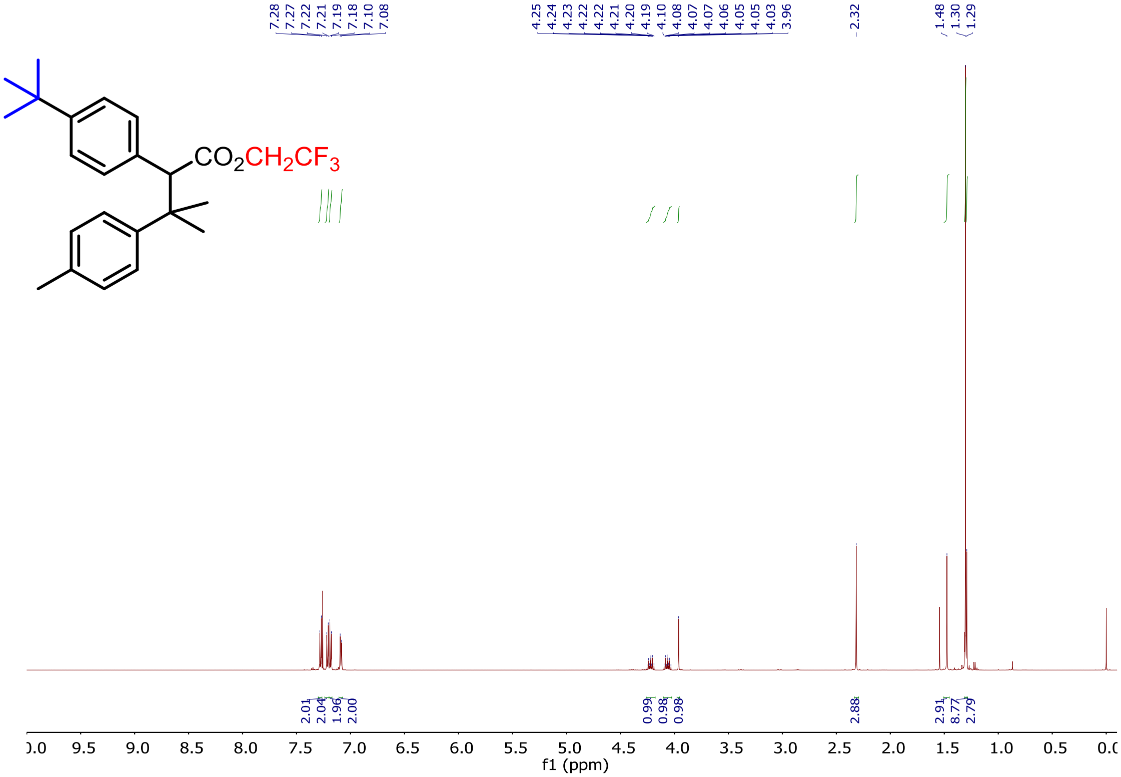
CO2CH2CF3
7.28
7.27
7.22
7.21
7.19
7.18
7.10
7.08
4.25
4.24
4.23
4.22
4.22
4.21
4.20
4.19
4.10
4.08
4.07
4.06
4.05
4.05
4.03
3.96
2.32
1.48
1.30
1.29
2.01
2.04
1.96
2.00
0.99
0.98
0.98
2.88
2.91
8.77
2.79
f1 (ppm)

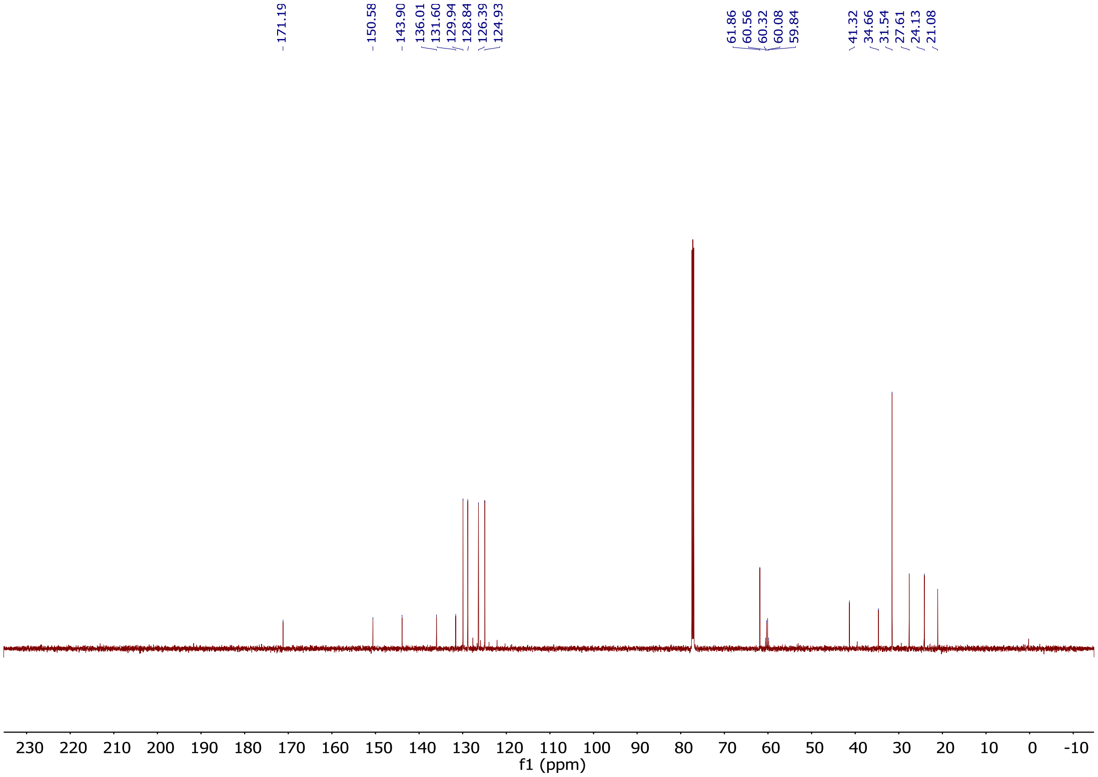
171.19
150.58
143.90
136.01
131.60
129.94
128.84
126.39
124.93
61.86
60.56
60.32
60.08
59.84
41.32
34.66
31.54
27.61
24.13
21.08
f1 (ppm)

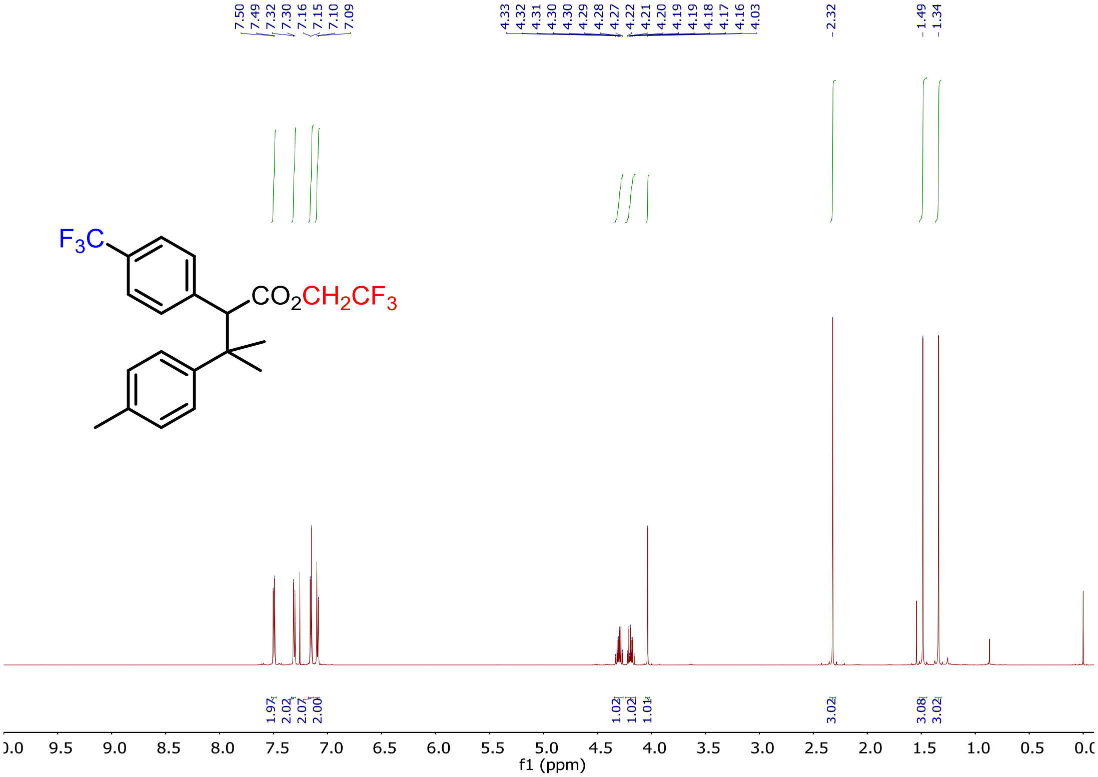

7.50
7.49
7.32
7.30
7.16
7.15
7.10
7.09
4.33
4.32
4.31
4.30
4.30
4.29
4.28
4.27
4.22
4.21
4.20
4.19
4.19
4.18
4.17
4.16
4.03
2.32
1.49
1.34
1.97
2.02
2.07
2.00
1.02
1.02
1.01
3.02
3.08
3.02
f1 (ppm)

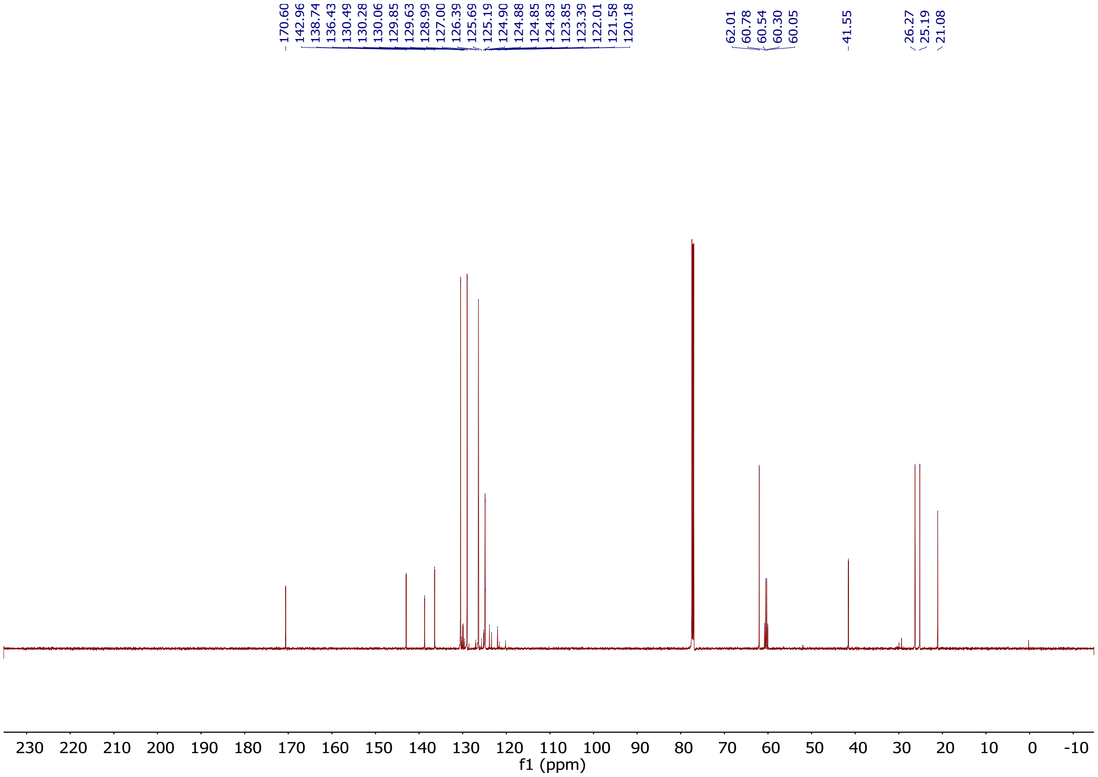

170.60
142.96
138.74
136.43
130.49
130.28
130.06
129.85
129.63
128.99
127.00
126.39
125.69
125.19
124.90
124.88
124.85
124.83
123.85
123.39
122.01
121.58
120.18
62.01
60.78
60.54
60.30
60.05
41.55
26.27
25.19
21.08
f1 (ppm)

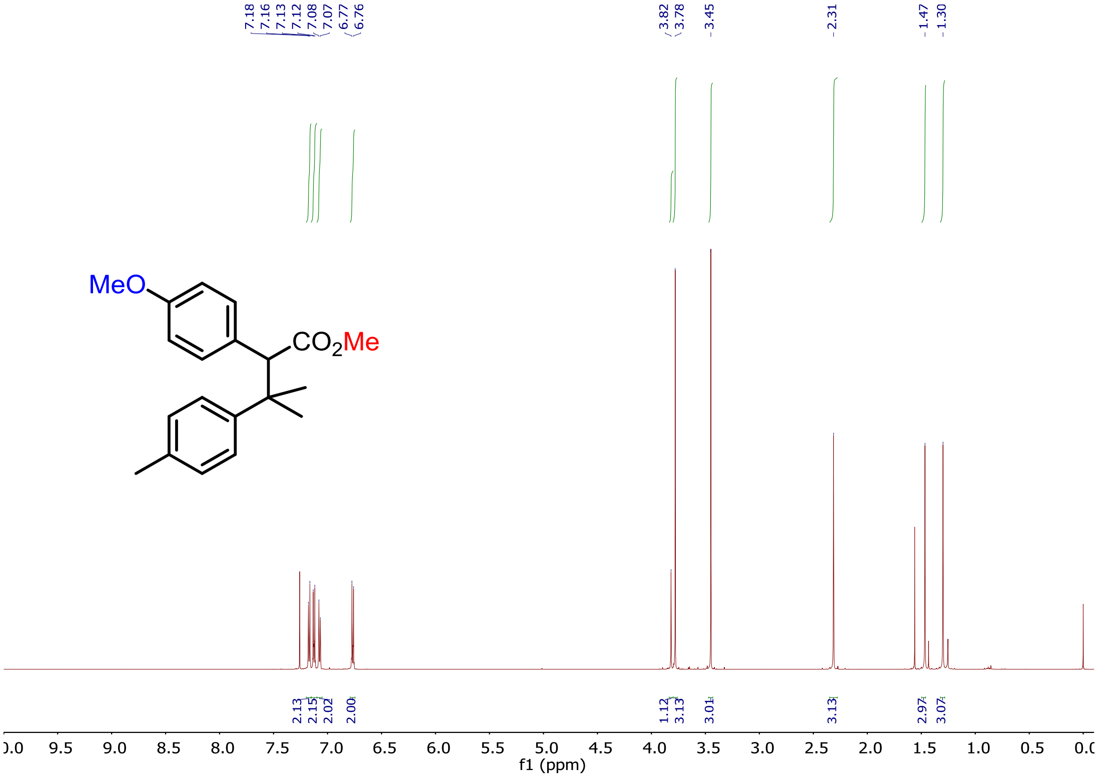
MeO
CO2Me
7.18
7.16
7.13
7.12
7.08
7.07
6.77
6.76
3.82
3.78
3.45
2.31
1.47
1.30
2.13
2.15
2.02
2.00
1.12
3.13
3.01
3.13
2.97
3.07
f1 (ppm)

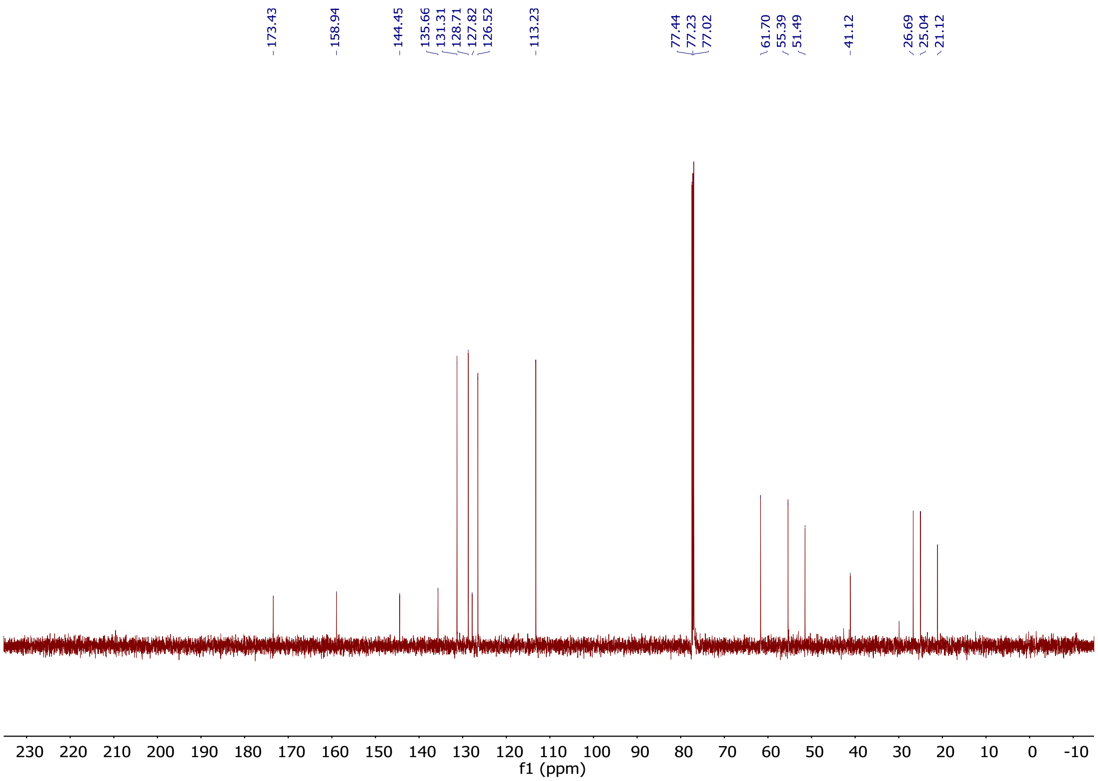
173.43
158.94
144.45
135.66
131.31
128.71
127.82
126.52
113.23
77.44
77.23
77.02
61.70
55.39
51.49
41.12
26.69
25.04
21.12
f1 (ppm)

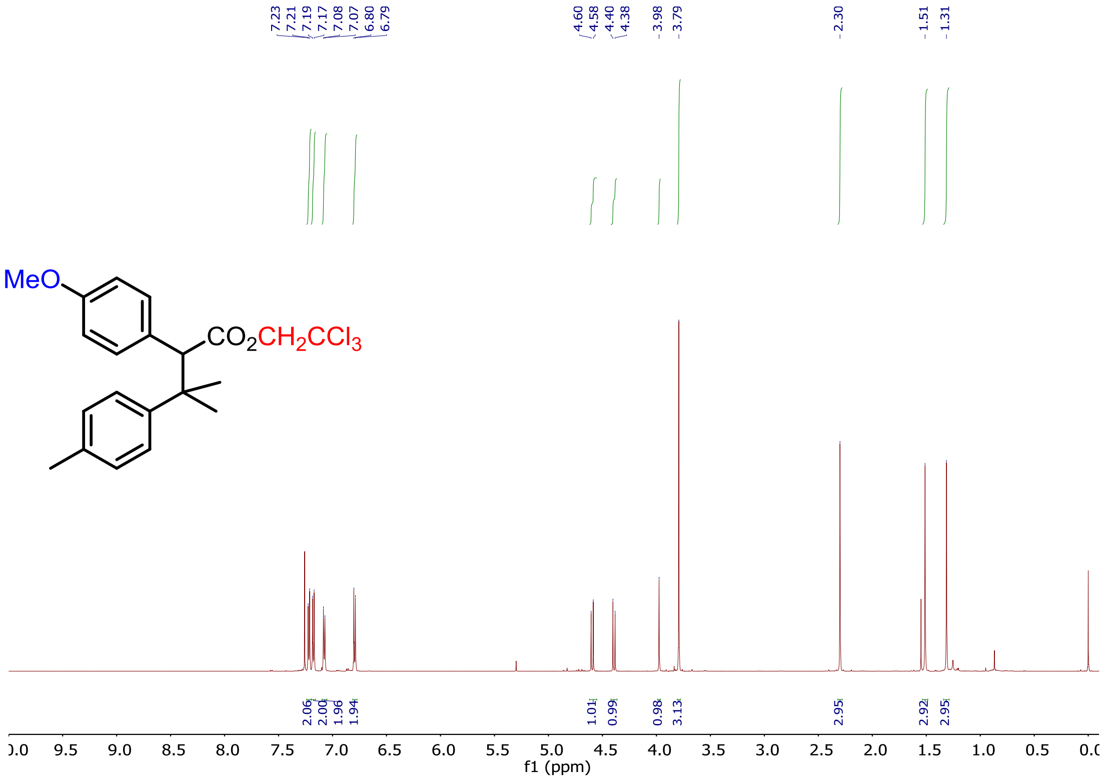
7.23
7.21
7.19
7.17
7.08
7.07
6.80
6.79
4.60
4.58
4.40
4.38
3.98
3.79
2.30
1.51
1.31
MeO
$CO_2CH_2CCl_3$
2.06
2.00
1.96
1.94
1.01
0.99
0.98
3.13
2.95
2.92
2.95
f1 (ppm)

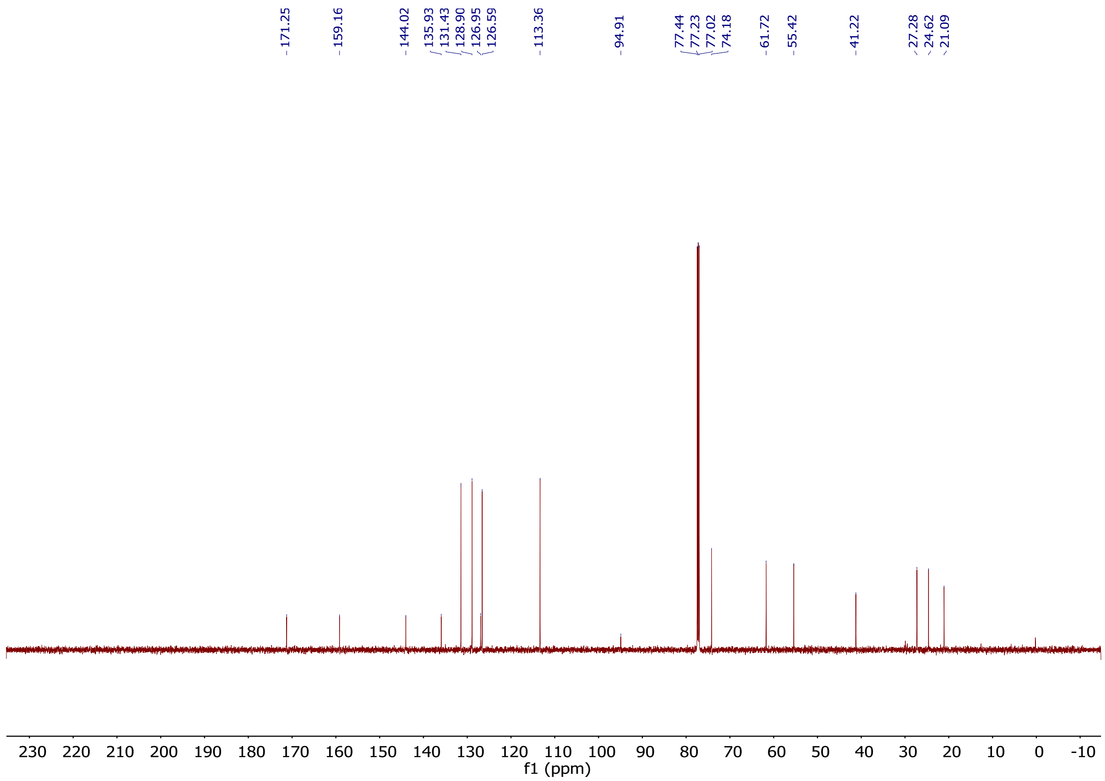
171.25
159.16
144.02
135.93
131.43
128.90
126.95
126.59
113.36
94.91
77.44
77.23
77.02
74.18
61.72
55.42
41.22
27.28
24.62
21.09
f1 (ppm)

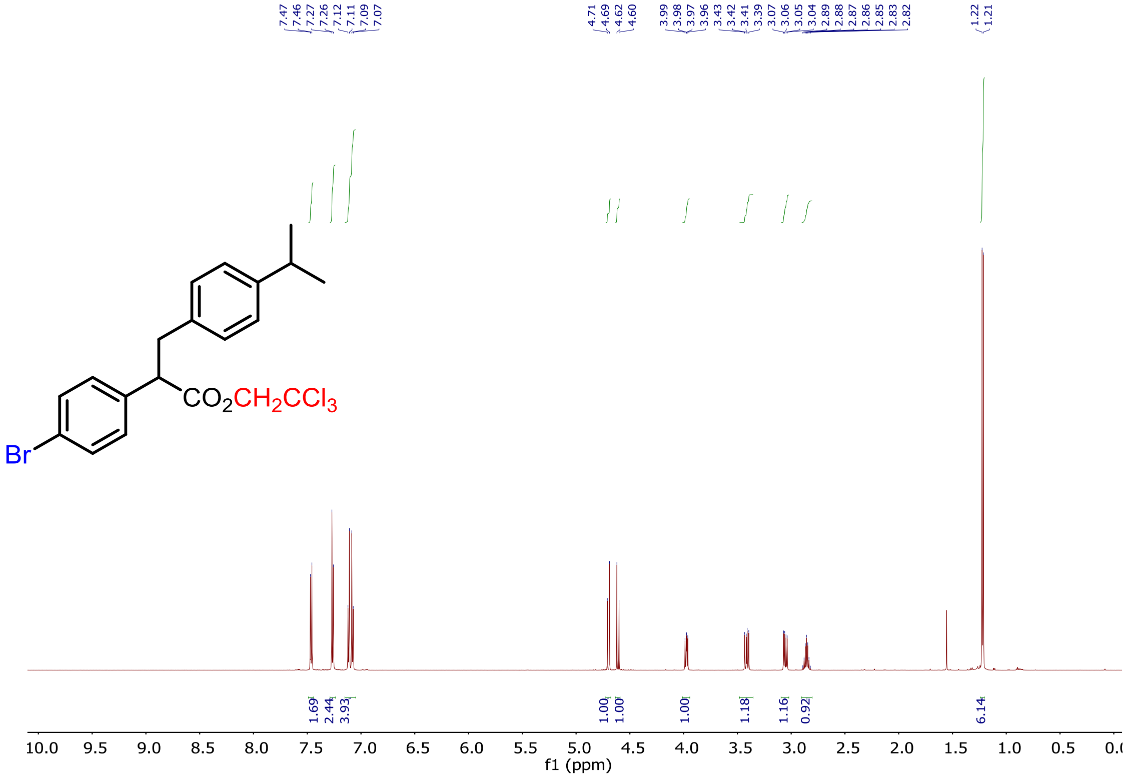
7.47
7.46
7.27
7.26
7.12
7.11
7.09
7.07
4.71
4.69
4.62
4.60
3.99
3.98
3.97
3.96
3.43
3.42
3.41
3.39
3.07
3.06
3.05
3.04
2.89
2.88
2.87
2.86
2.85
2.83
2.82
1.22
1.21
$CO_2CH_2CCl_3$
Br
1.69
2.44
3.93
1.00
1.00
1.00
1.18
1.16
0.92
6.14
f1 (ppm)

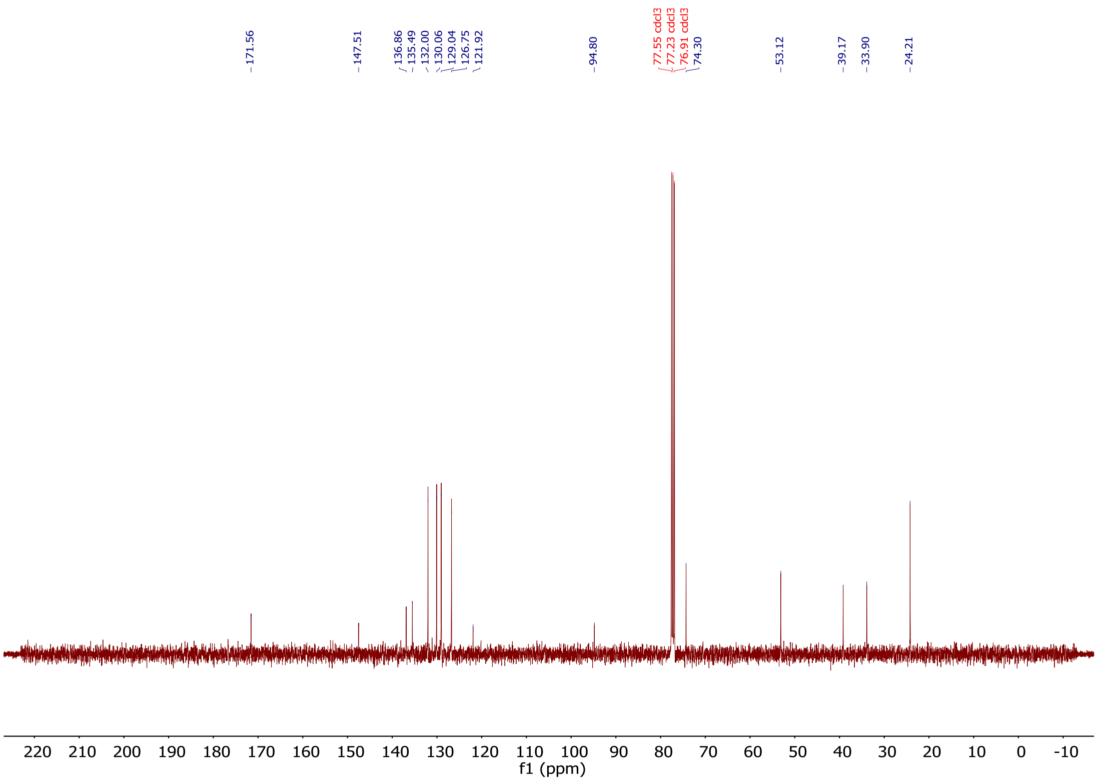
171.56
147.51
136.86
135.49
132.00
130.06
129.04
126.75
121.92
94.80
77.55 cdcl3
77.23 cdcl3
76.91 cdcl3
74.30
53.12
39.17
33.90
24.21
f1 (ppm)

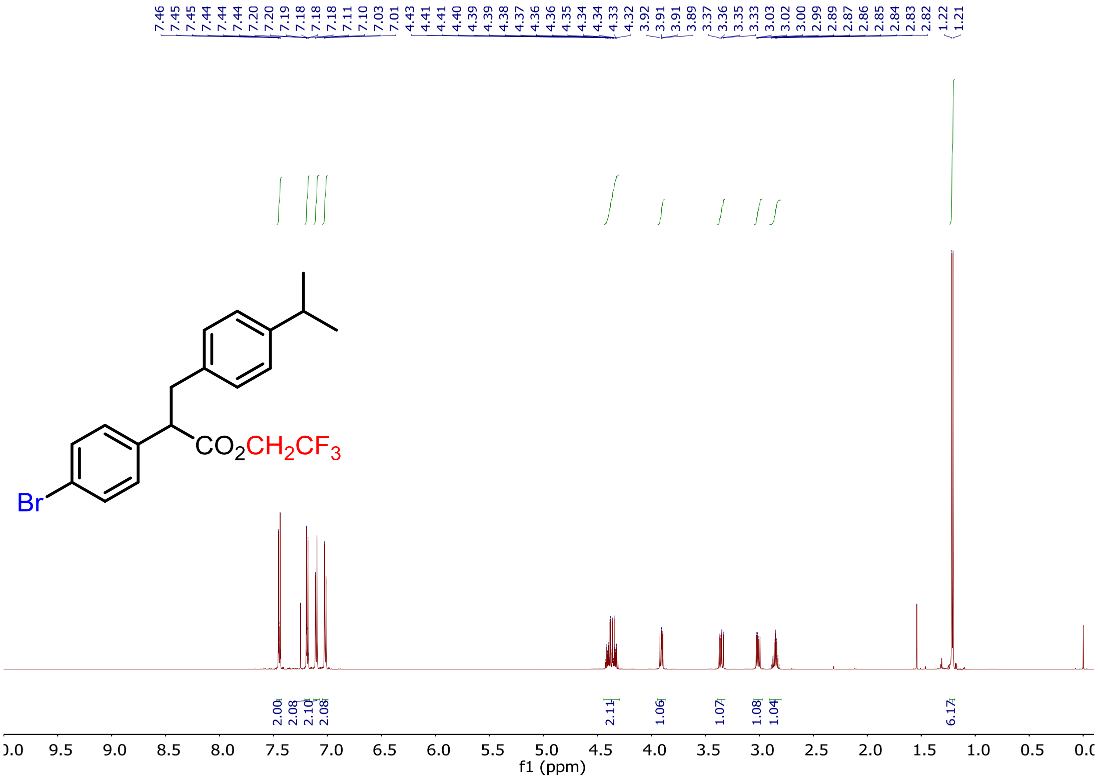
7.46 7.45 7.45 7.44 7.44 7.44 7.20 7.20 7.19 7.18 7.18 7.18 7.11 7.10 7.03 7.01 4.43 4.41 4.41 4.40 4.39 4.39 4.38 4.37 4.36 4.36 4.35 4.34 4.34 4.33 4.32 3.92 3.91 3.91 3.89 3.37 3.36 3.35 3.33 3.03 3.02 3.00 2.99 2.89 2.87 2.86 2.85 2.84 2.83 2.82 1.22 1.21
CO2CH2CF3
Br
2.00 2.08 2.10 2.08 2.11 1.06 1.07 1.08 1.04 6.17
0.0 9.5 9.0 8.5 8.0 7.5 7.0 6.5 6.0 5.5 5.0 4.5 4.0 3.5 3.0 2.5 2.0 1.5 1.0 0.5 0.0
f1 (ppm)

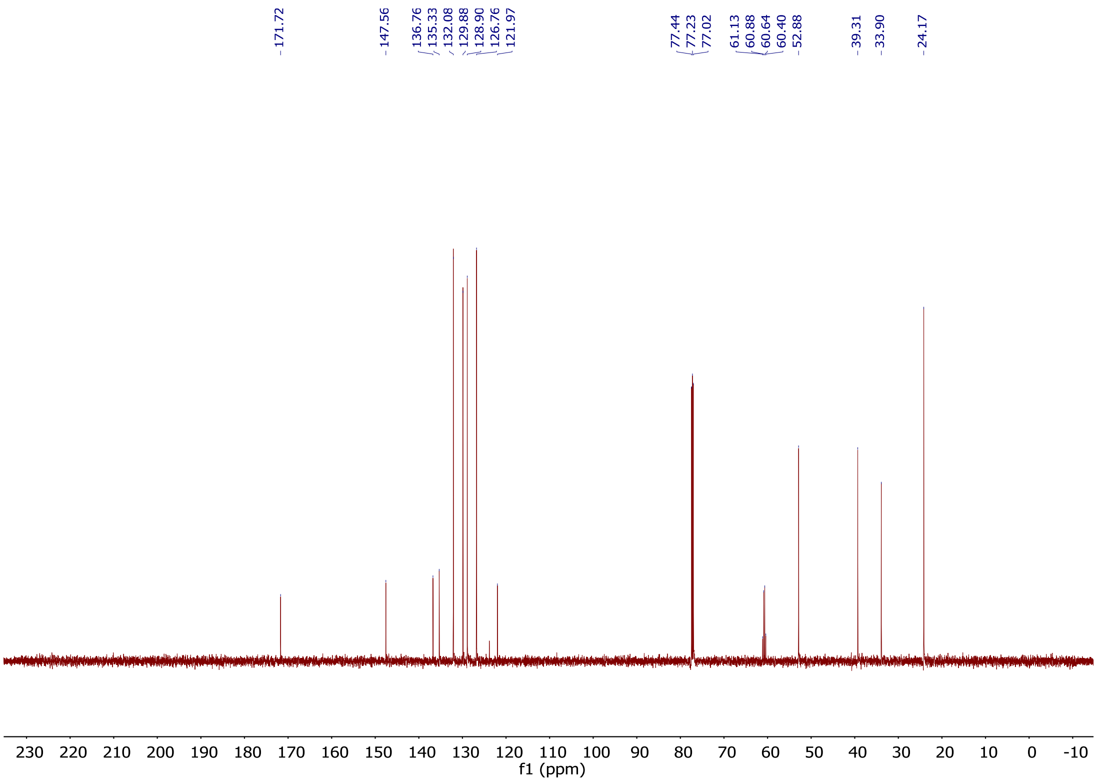
171.72 147.56 136.76 135.33 132.08 129.88 128.90 126.76 121.97 77.44 77.23 77.02 61.13 60.88 60.64 60.40 52.88 39.31 33.90 24.17
230 220 210 200 190 180 170 160 150 140 130 120 110 100 90 80 70 60 50 40 30 20 10 0 -10
f1 (ppm)

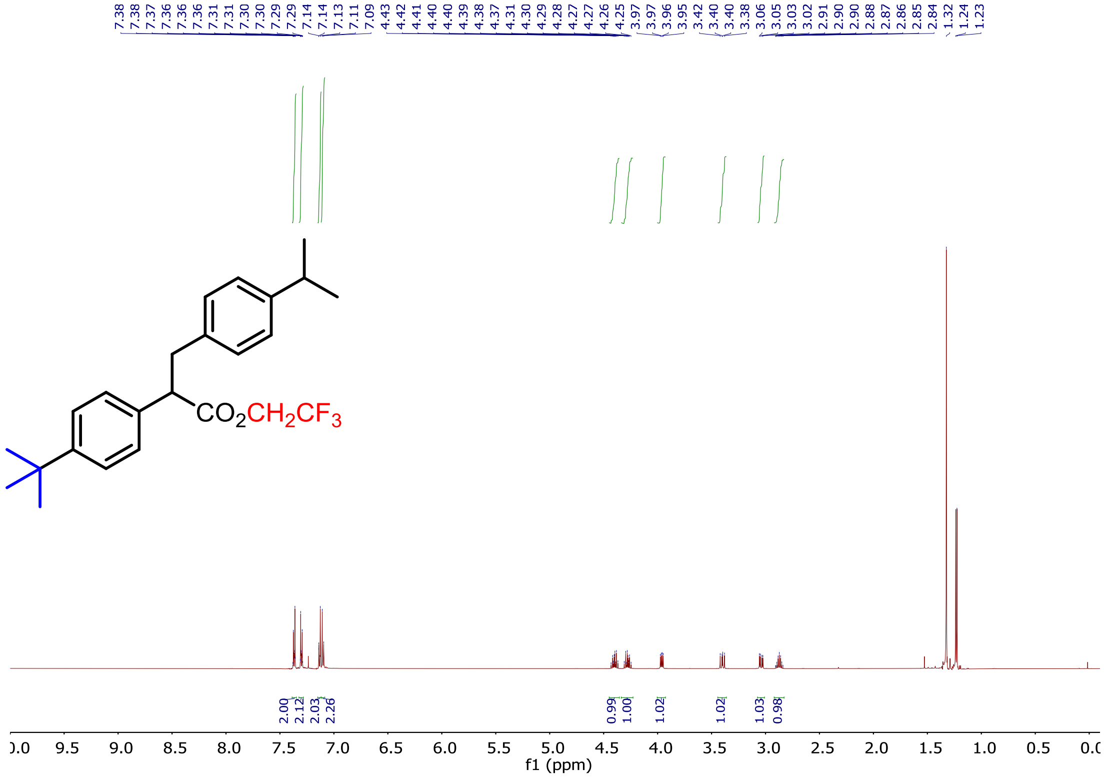

7.38 7.38 7.37 7.36 7.36 7.36 7.31 7.31 7.30 7.30 7.29 7.29 7.14 7.14 7.13 7.11 7.09 4.43 4.42 4.41 4.40 4.40 4.39 4.38 4.37 4.31 4.30 4.29 4.28 4.27 4.27 4.26 4.25 3.97 3.97 3.96 3.95 3.42 3.40 3.40 3.38 3.06 3.05 3.03 3.02 2.91 2.90 2.90 2.88 2.87 2.86 2.85 2.84 1.32 1.24 1.23
$CO_2CH_2CF_3$
2.00 2.12 2.03 2.26 0.99 1.00 1.02 1.02 1.03 0.98
f1 (ppm)

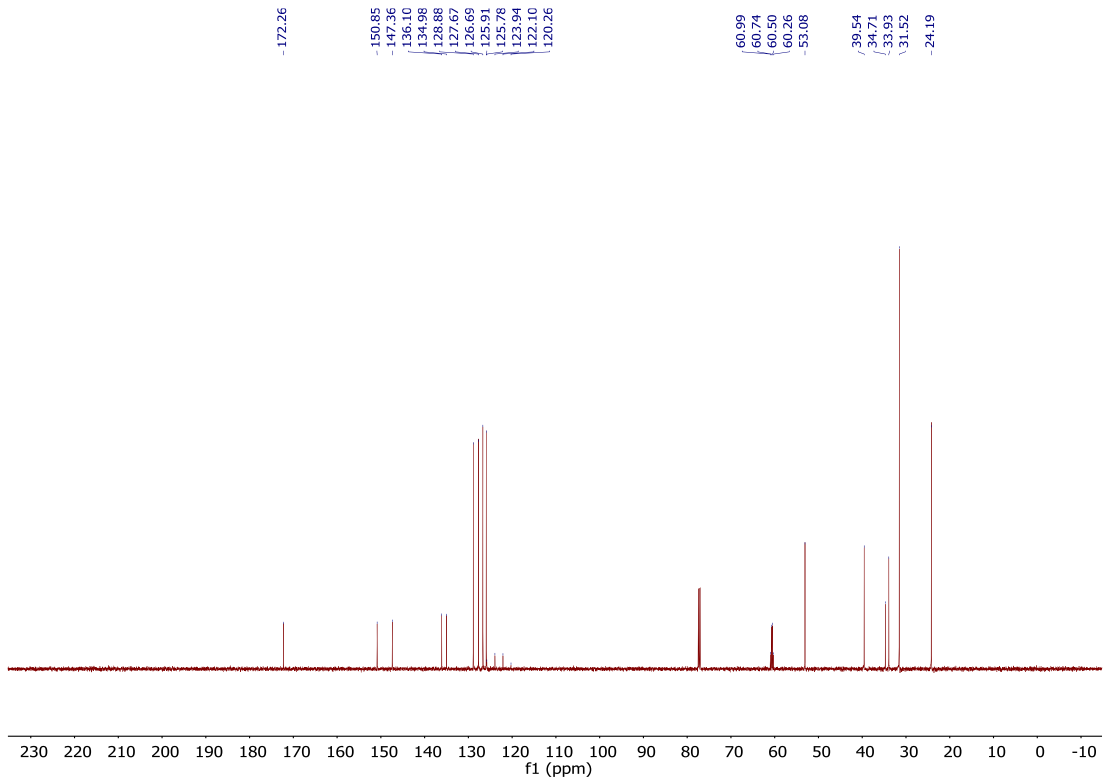

172.26 150.85 147.36 136.10 134.98 128.88 127.67 126.69 125.91 125.78 123.94 122.10 120.26 60.99 60.74 60.50 60.26 53.08 39.54 34.71 33.93 31.52 24.19
f1 (ppm)

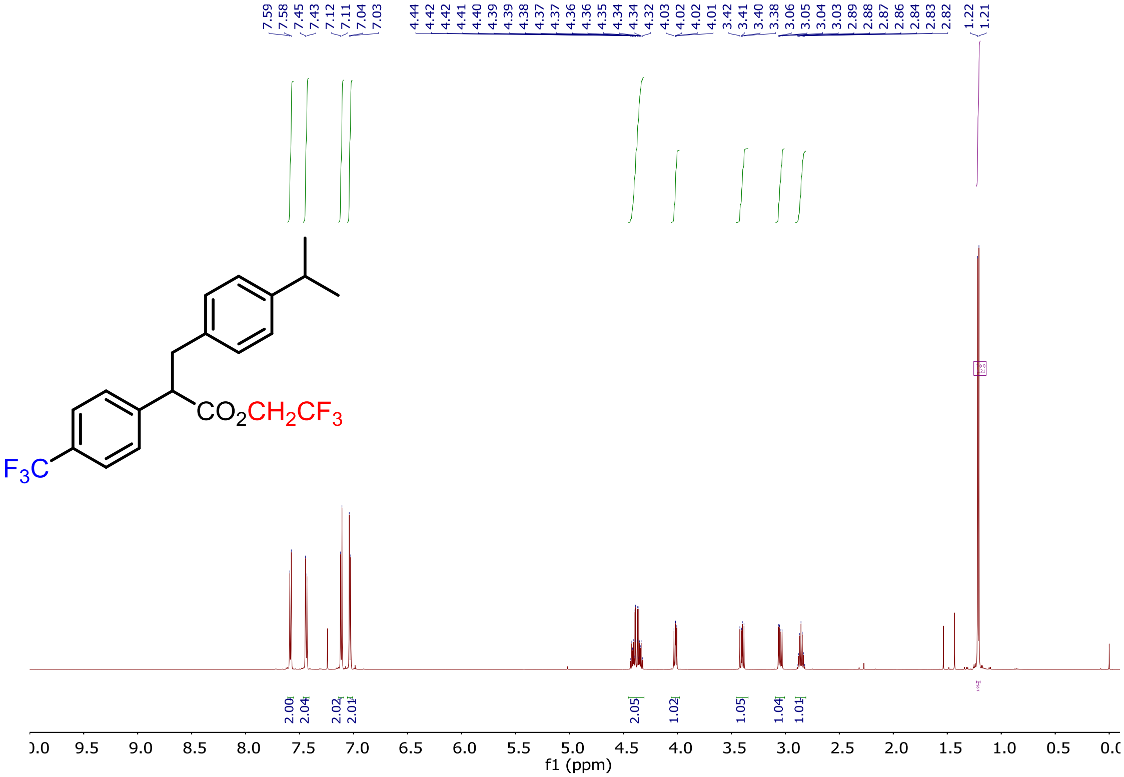
7.59
7.58
7.45
7.43
7.12
7.11
7.04
7.03
4.44
4.42
4.42
4.41
4.40
4.39
4.39
4.38
4.37
4.36
4.36
4.35
4.34
4.34
4.32
4.03
4.02
4.02
4.01
3.42
3.41
3.40
3.38
3.06
3.05
3.04
3.03
2.89
2.88
2.87
2.86
2.84
2.83
2.82
1.22
1.21
$CO_2CH_2CF_3$
F_3C
2.00
2.04
2.02
2.01
2.05
1.02
1.05
1.04
1.01
f1 (ppm)

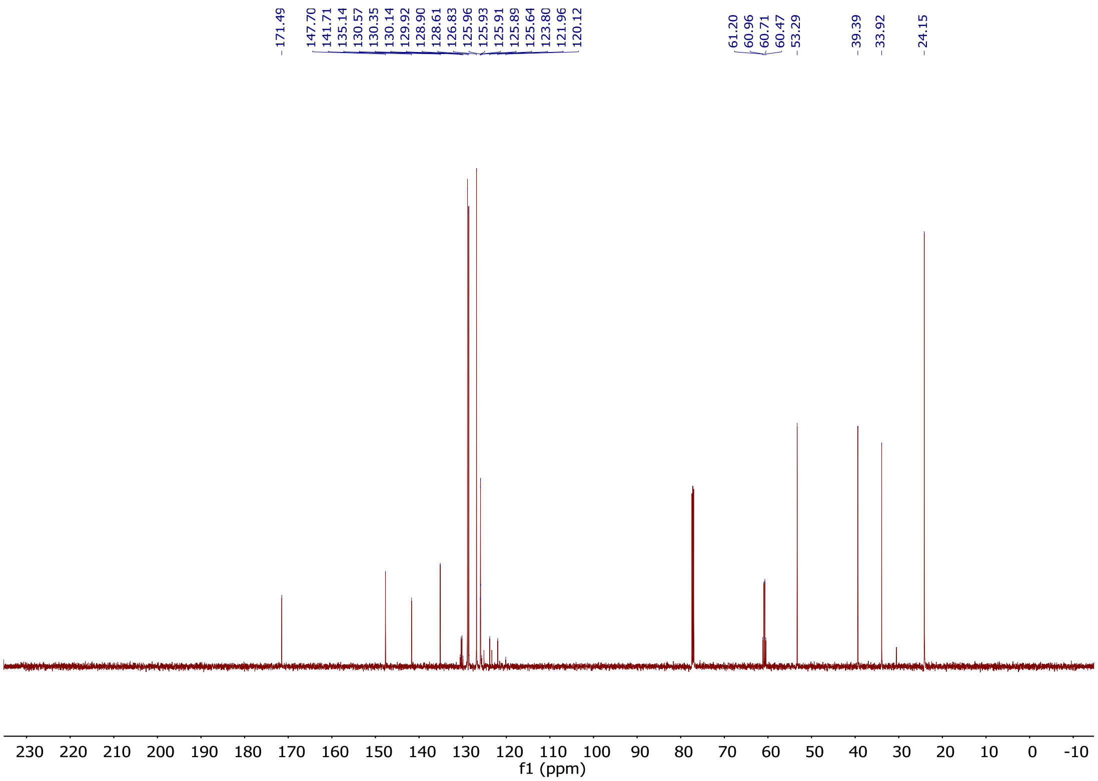
171.49
147.70
141.71
135.14
130.57
130.35
130.14
129.92
128.90
128.61
126.83
125.96
125.93
125.91
125.89
125.64
123.80
121.96
120.12
61.20
60.96
60.71
60.47
53.29
39.39
33.92
24.15
f1 (ppm)

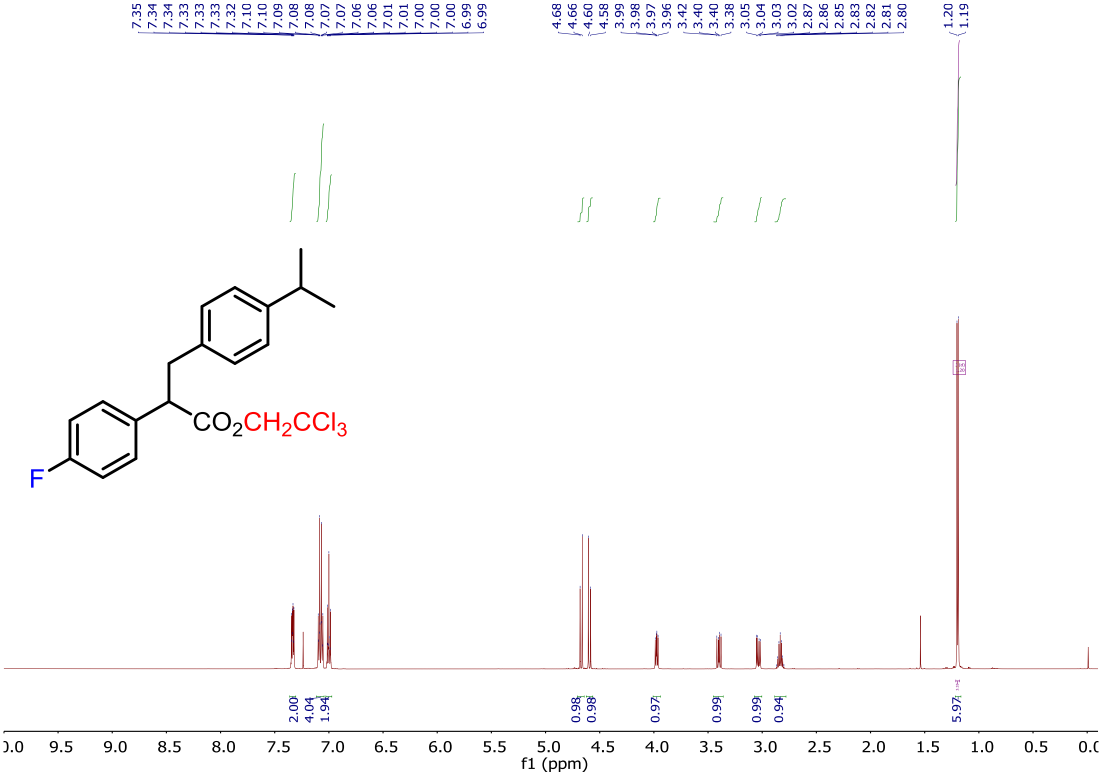
7.35 7.34 7.34 7.33 7.33 7.33 7.32 7.10 7.10 7.09 7.08 7.08 7.07 7.07 7.06 7.06 7.01 7.01 7.00 7.00 6.99 6.99
4.68 4.66 4.60 4.58 3.99 3.98 3.97 3.96 3.42 3.40 3.40 3.38 3.05 3.04 3.03 3.02 2.87 2.86 2.85 2.83 2.82 2.81 2.80
1.20 1.19
F
$CO_2CH_2CCl_3$
2.00 4.04 1.94 0.98 0.98 0.97 0.99 0.99 0.94 5.97
f1 (ppm)

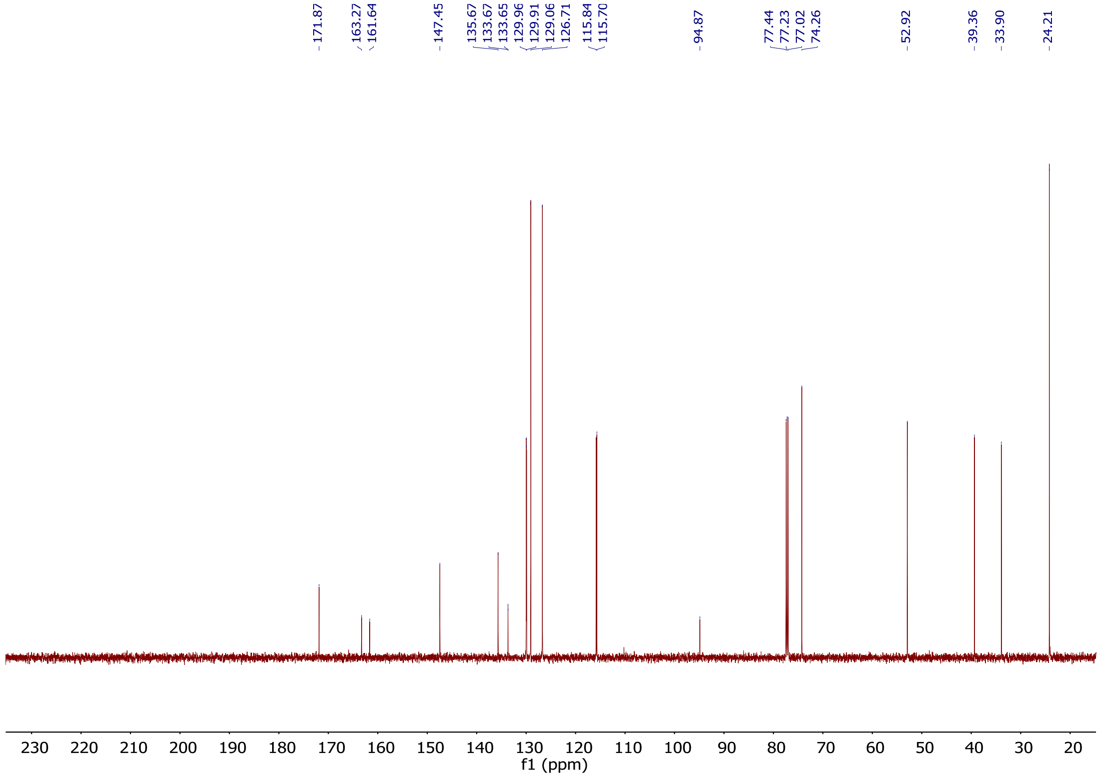
171.87 163.27 161.64 147.45 135.67 133.67 133.65 129.96 129.91 129.06 126.71 115.84 115.70 94.87 77.44 77.23 77.02 74.26 52.92 39.36 33.90 24.21
f1 (ppm)
